# Hydrosilylation of a Molecular Molybdenum Nitride Provides Mechanistic Insights into Photodriven Ammonia Synthesis from N_2_ and H_2_

**DOI:** 10.1021/jacs.5c22220

**Published:** 2026-02-10

**Authors:** Junho Kim, Nidhi Kaul, Matthew V. Pecoraro, Paul J. Chirik

**Affiliations:** Department of Chemistry, 6740 Princeton University, Princeton, New Jersey 08544, United States

## Abstract

Addition of Ph_2_SiH_2_ to [(depe)_2_Mo­(N)]­[BAr^F^_4_] (depe = 1,2-bis­(diethylphosphino)­ethane, BAr^F^_4_ = B­(3,5-(CF_3_)_2_C_6_H_3_)_4_) at 60 °C generated the silyl imido molybdenum hydride complex, *trans*-[(depe)_2_Mo­(NSiHPh_2_)­H]­[BAr^F^_4_], a surrogate for a proposed intermediate complex in the photodriven hydrogenation to free ammonia. Irradiation of a THF solution of *trans*-[(depe)_2_Mo­(NSiHPh_2_)­H]­[BAr^F^_4_] with blue light under H_2_ produced free amine along with [(depe)_2_MoH_5_]­[BAr^F^_4_] in 76% yield. This transformation occurred in the absence of a precious metal photocatalyst, suggesting that it was needed only for the initial addition of H_2_ to the molybdenum nitride during the first N–H bond-forming step in the photodriven hydrogenation. Deuterium labeling and crossover studies support concerted Si–H bond addition across the Mo≡N bond, enabled by the nucleophilicity of the nitride. Subsequent hydrogenation involves an intramolecular H migration from Mo to the imido ligand, as supported by electronic absorption spectroscopy, transient absorption spectroscopy, initial rate measurements, and deuterium kinetic isotope effect measurements. These findings provide insights into the photodriven hydrogenation of [(depe)_2_Mo­(N)]­[BAr^F^_4_] to ammonia and the role of the photocatalyst in this transformation.

## Graphical abstract

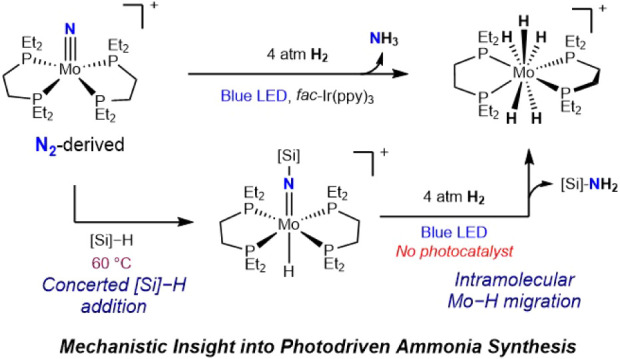

## Introduction

The direct synthesis of ammonia from N_2_ and H_2_ is atom-economical and eliminates waste and chemical overpotential;^1–4^ however, promoting this transformation with molecular transition metal compounds remains a long-standing challenge. Although the cleavage of N_2_ by reduced metal complexes is well established,^5,6^ subsequent hydrogenation of the resulting metal-nitrides is less precedented, with only a few reported examples and typically low yields of NH_3_.^7–13^ This lack of reactivity stems from the intrinsic thermodynamics of the hydrogenation reaction, as cleavage of the strong N≡N bond generates a stable metal-nitride that forms comparatively weak N–H bonds upon addition of hydrogen. In many cases, the N–H bonds are sufficiently weak and fall below the energetic requirements for productive N–H bond formation.^10,14^

The formation of N–H bonds from N_2_-derived metal nitride and dinitrogen complexes from reaction with H_2_ has been observed in a host of examples, including first-row, early transition metal, and actinide complexes.^15^ However, few of these examples result in continued hydrogenation to ammonia, leaving the key intermediates and mechanisms of productive N–H bond formation and release of NH_3_ from the coordination sphere of the metal as poorly understood. The zirconocene hydrido diazenido complex was identified as an intermediate following the first H_2_ addition to the corresponding side-on bound zirconocene dinitrogen compound (Scheme 1).^7^ Walter and coworkers have reported iron bridging imido complexes arising from initial H_2_ addition to a bridging iron nitride, although additional studies are needed to clarify their role in N–H bond-forming chemistry.^8^ With other metal complexes, higher yields of NH_3_ have been achieved through the use of stoichiometric additives, resulting in stoichiometric waste and chemical overpotential.^16–18^ In one notable example, Hidai and coworkers reported a tungsten hydrazido compound as an intermediate surrogate en route to NH_3_ synthesis under acidic conditions.^16^

**Scheme 1:**
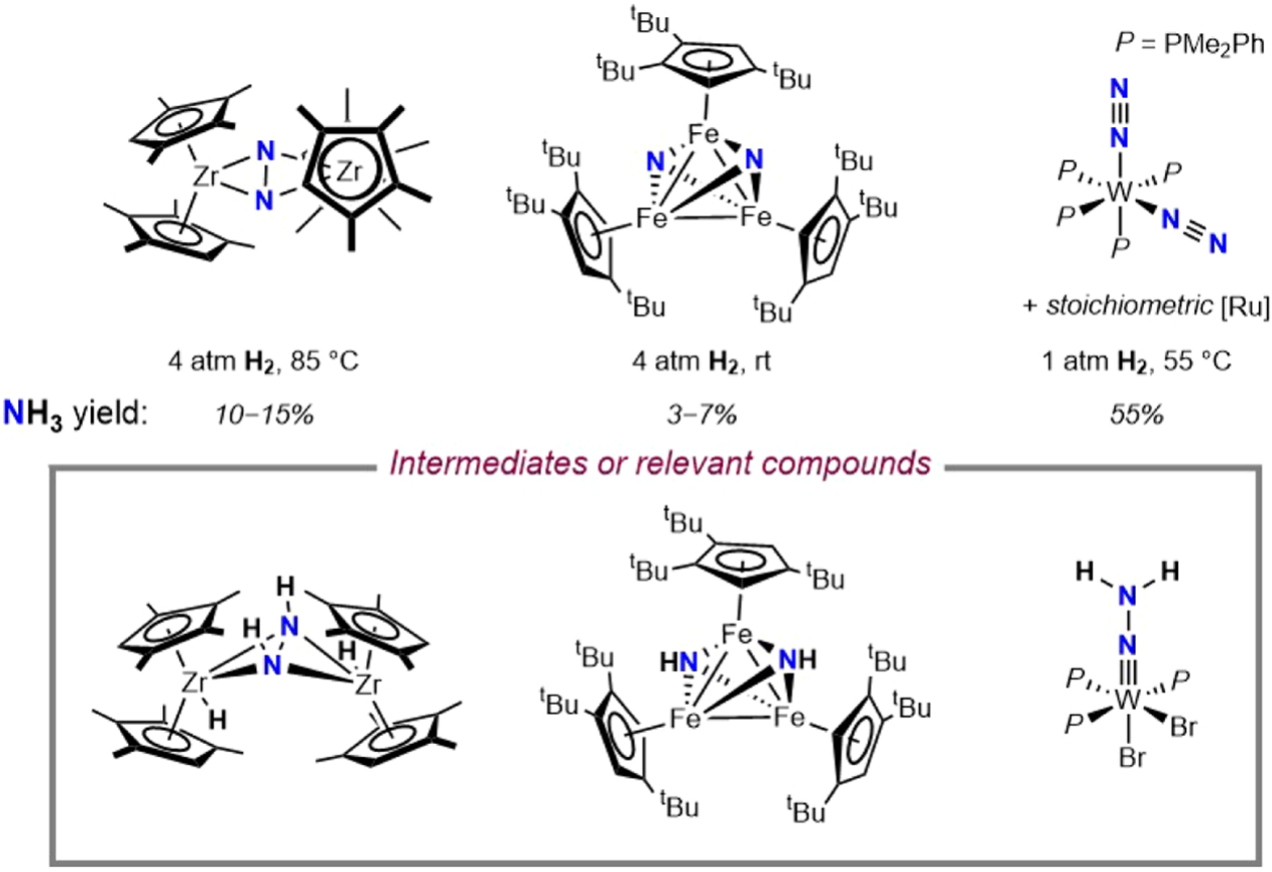
Selected, Previously Reported Examples of the Hydrogenation of N_2_-Derived Transition Metal Compounds and Proposed Intermediates or Relevant Compounds

Our group has recently reported the photodriven hydrogenation of the N_2_-derived molecular molybdenum nitride, [(depe)_2_MoN]­[BAr^F^_4_] (**Mo1**; **depe** = 1,2-bis­(diethylphosphino)­ethane)^19^ to yield free ammonia using piano-stool iridium hydrides^10^ and iridium tris­(phenyl)­pyridine (Ir­(ppy)_3_) photocatalysts.^20^ With Ir­(ppy)_3_, ammonia formation was accompanied by clean generation of the molybdenum pentahydride product, [(depe)_2_MoH_5_]­[BAr^F^_4_] (**Mo2**), that was recycled to the starting metal nitride and enabled superstoichiometric NH_3_ synthesis (Scheme 2a).^20^ While these reports demonstrate the feasibility of ammonia synthesis from the hydrogenation of N_2_-derived molecular nitrides, the role of visible light and the nature of the intermediates in the multistep transformation remain elusive.

**Scheme 2:**
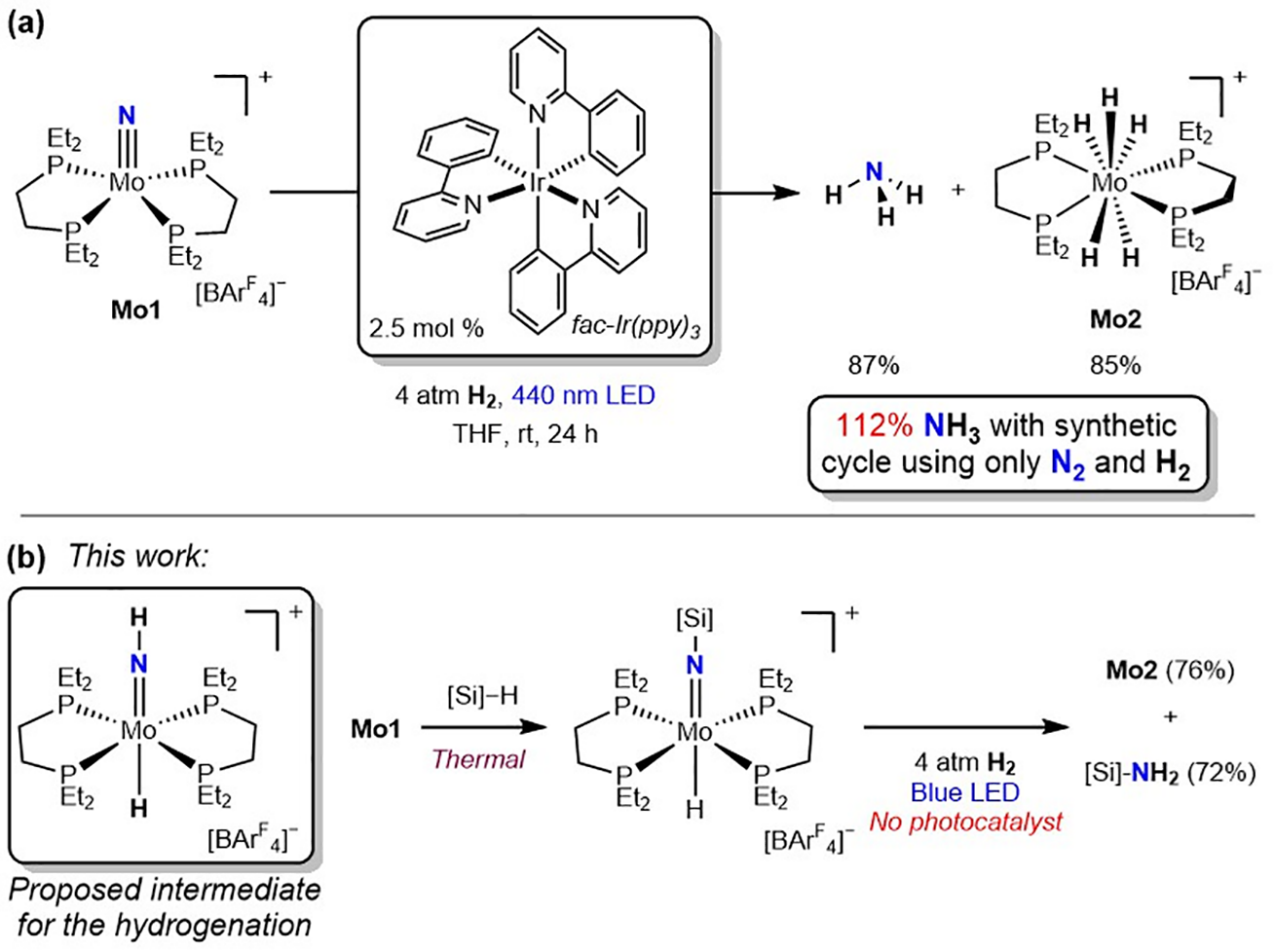
(a) Photodriven Hydrogenation of **Mo1** Using an Iridium Photocatalyst. (b) This Work: Stepwise N_2_ Reduction to the Free Amine Following Initial Thermal Addition of Silane

The addition of Si–H bonds of organosilanes to transition metal nitrides has been extensively studied as a surrogate for H_2_ addition.^21–27^ Despite this interest, the products of silane addition to metal nitrides have not been explored as intermediates in hydrogenation using molecular H_2_ to free amines. The initial hydrosilylation of **Mo1** was envisioned as a route to molybdenum silylimido hydride complexes that resemble the first step in the photodriven hydrogenation of **Mo1**. Here we describe the successful demonstration of this approach for the preparation of a series of molybdenum silyl imido hydride complexes under thermal conditions. Subsequent hydrogenation with irradiation using blue light in the absence of a precious metal photocatalyst resulted in the liberation of the free amine along with **Mo2** (Scheme 2b).

## Results and Discussion

### Hydrosilylation of Mo1

Our studies commenced with the hydrosilylation of **Mo1** using diphenylsilane (Ph_2_SiH_2_) (Figure 1a). While no conversion was observed at ambient temperature for 18 h, heating a 9 mM THF-*d*_*8*_ solution of **Mo1** and 10 equiv of Ph_2_SiH_2_ to 60 °C for 18 h produced a color change from red to violet. A singlet was observed at 65.8 ppm in the THF-*d*_8_^31^P NMR spectrum along with a diagnostic quintet at −6.88 ppm in the ^1^H NMR spectrum, signaling the formation of a Mo–H coupled to four equivalent phosphines. On a preparative scale, the hydrosilylation procedure was optimized in diethyl ether solution to avoid competing THF polymerization. The silylimido molybdenum hydride, **Mo4**, was isolated in 97% yield following recrystallization from a diethyl ether-pentane solution. The solid-state structure of **Mo4** was determined by X-ray diffraction (Figure 1a). An overall idealized octahedral geometry was observed for molybdenum with *trans* imido and hydride ligands. The bond lengths of the Mo=N and Mo–H bonds were determined as 1.793(10) and 1.87(8) Å, respectively, which are consistent with those of previously reported molybdenum imido hydride compounds,^23,27^ confirming the presence of imido and hydrido ligands.

**Figure 1:**
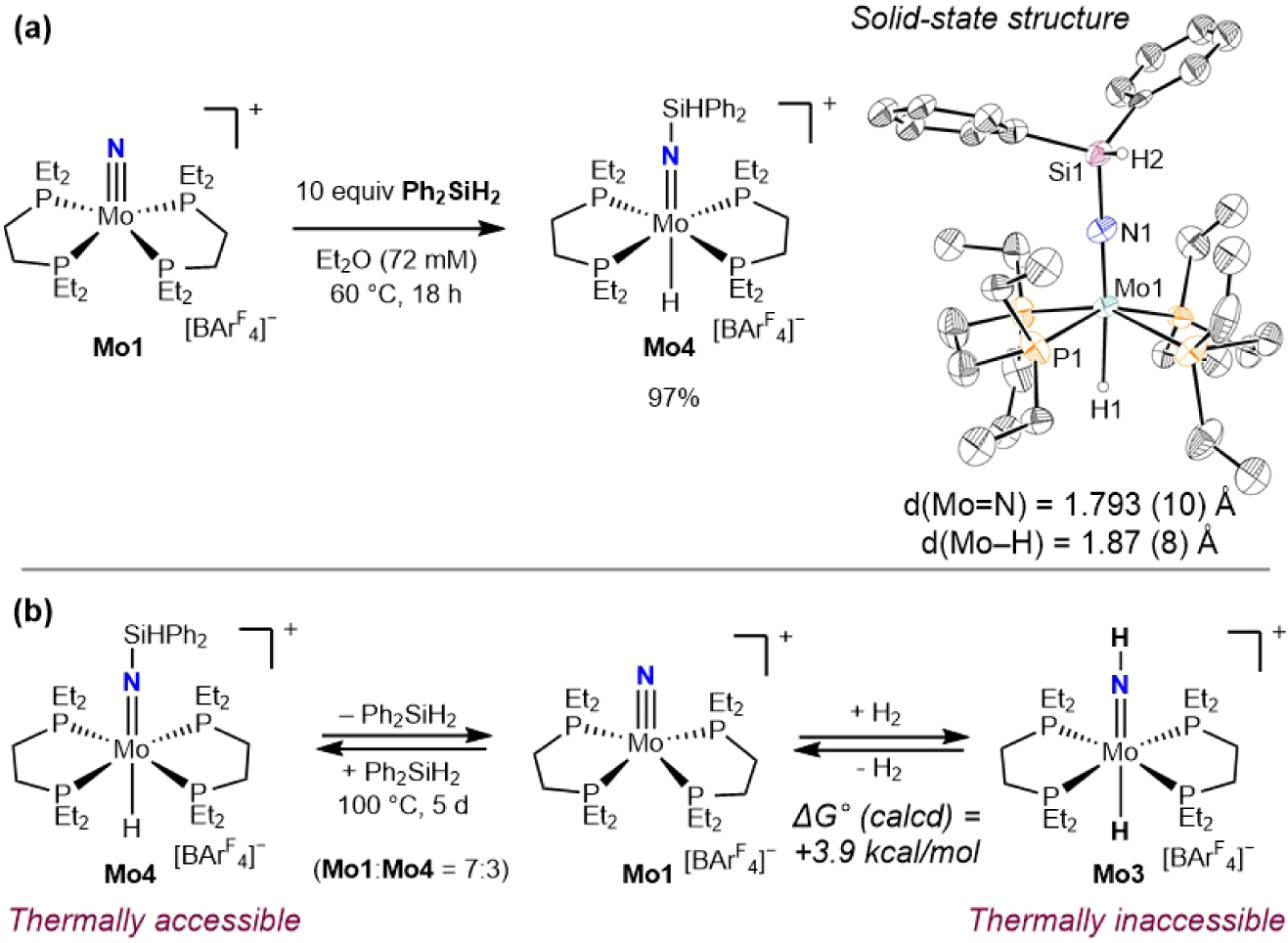
(a) Synthesis of **Mo4** and a representation of the solid-state structure at 30% probability of ellipsoids. [BAr^F^_4_]^−^ and hydrogen atoms except H1 and H2 are omitted for clarity. (b) Comparison of reaction thermodynamics for the addition of H_2_ and Ph_2_SiH_2_ to **Mo1**.

The observed product, where an N–Si and Mo–H bond are formed, is consistent with a nucleophilic nitride ligand and is expected for a *d* ^*2*^ Mo­(IV) complex in an idealized square pyramidal ligand field with an occupied nonbonding orbital centered on nitrogen and empty π* orbitals of the metal-nitride bond that have a larger contribution from Mo-centered *d*-orbitals.^19,28–30^ These findings are corroborated by previous reports, including NH_3_ coordination to the Mo center instead of reaction with the nitride ligand of **Mo1**.^20^ Relatedly, the addition of excess HCl to **Mo1** generated the previously reported imido chloride compound [(depe)_2_Mo­(NH)­Cl]­[Cl],^31^ further supporting the assignment of **Mo1** as a nucleophilic nitride.

Heating a THF-*d*_8_ solution of **Mo4** to 60 °C for 24 h produced no change (Figure 1b). Additional heating to 100 °C in a sealed J. Young NMR tube over the course of 5 days resulted in regeneration of **Mo1** and liberation of the free silane. Under these conditions, an equilibrium was established, generating a 7:3 mixture of **Mo1** and **Mo4**, indicating that **Mo4** is a thermally accessible intermediate surrogate for the *trans*-molybdenum parent imido hydride, **Mo3**, using Ph_2_SiH_2_ instead of H_2_ (Figure S23). In contrast, the calculated standard Gibbs free energy (Δ*G*°) for H_2_ addition to **Mo1** was found to be +3.9 kcal/mol, establishing an endergonic process for the formation of **Mo3**. This result is consistent with the experimental observation that the addition of H_2_ to **Mo1** does not occur under thermal conditions under 1–4 atm of pressure.

### Scope and Mechanistic Investigations for the Hydrosilylation of Mo1

The hydrosilylation of **Mo1** with different silanes was investigated (Scheme 3). Addition of 10 equiv of phenylsilane (PhSiH_3_) to a THF-*d*_*8*_ solution of **Mo1** at ambient temperature for 18 h resulted in hydrosilylation and furnished **Mo5** in near-quantitative yield, establishing that the barrier for addition of the primary silane was lower than that for Ph_2_SiH_2_. Treatment of **Mo1** with hindered, tertiary silanes such as triphenylsilane (Ph_3_SiH), triethylsilane (Et_3_SiH), and triethoxysilane ((EtO)_3_SiH), produced no reaction even upon heating to 100 °C for 18 h.

**Scheme 3:**
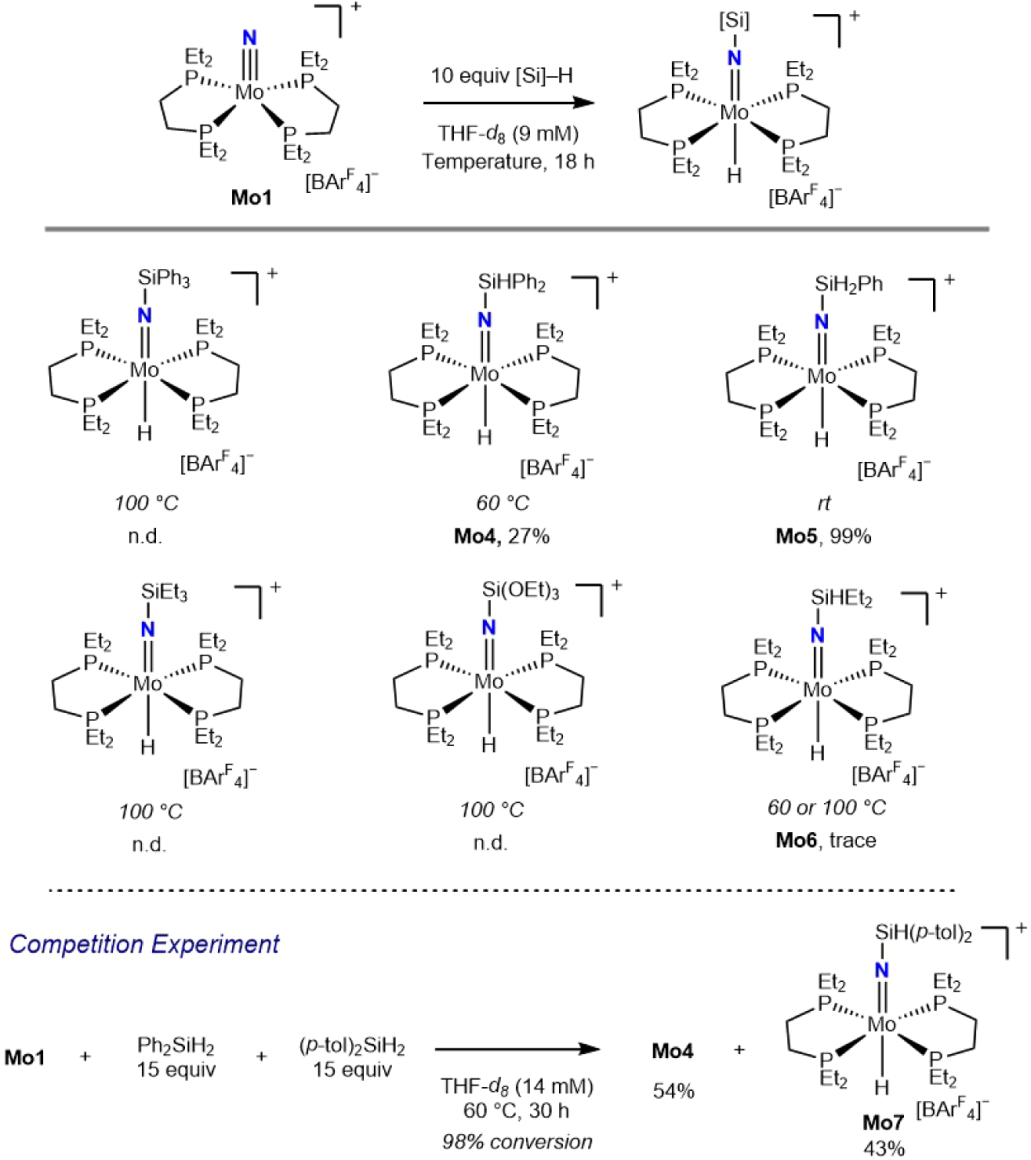
Scope of the Hydrosilylation of **Mo1** with Various Silanes

Addition of the more electron-rich but sterically accessible silane, Et_2_SiH_2_, to a THF-*d*_8_ solution of **Mo1** and heating to 100 °C for 18 h produced only trace conversion to **Mo6** with the recovery of the starting materials, establishing a slower hydrosilylation reaction. To further probe this effect, a competition experiment was conducted by using a mixture of Ph_2_SiH_2_ and di-*p*-tolylsilane ((*p*-tol)_2_SiH_2_). Heating a 14 mM solution of **Mo1** in THF-*d*_*8*_ with 15 equiv each of Ph_2_SiH_2_ and (*p*-tol)_2_SiH_2_ to 60 °C for 30 h generated a mixture of **Mo4** and a hydrosilylated product from (*p*-tol)_2_SiH_2_, **Mo7**, in 54% and 43% yield, respectively, further supporting that hydrosilylation occurs more slowly with more electron-rich silanes (Figure S1).

To gain additional insight into the mechanism of hydrosilylation, a crossover experiment was conducted with **Mo1**, 15 equiv of diphenylsilane-*d*_2_ (Ph_2_SiD_2_) and 15 equiv of ((*p*-tol)_2_SiH_2_) (Scheme 4a). In the case of a four-membered, concerted addition of the Si–H bond at a single metal center, two molybdenum products, **Mo4-** *d*_**2**_ and **Mo7**, are expected. If the reaction proceeds through two molybdenum centers^32^ or involves a stepwise mechanism with Si–H bond cleavage followed by rebound,^22^ four products—**Mo4-** *d*_**2**_, **Mo7**, **Mo4-(Si-** *d* **)**, and **Mo7-** *d*—are expected. In a control experiment, heating the mixture in a 14 mM THF-*d*_8_ solution for 18 h, Ph_2_SiHD and (*p*-tol)_2_SiHD, along with all four molybdenum products, were detected by ^1^H NMR spectroscopy, signaling the precedented H/D exchange reaction between silanes and metal hydrides,^33^ including those with previously reported molybdenum imido hydrido complexes.^34^ However, H/D exchange between Si–H­(D) and Mo–D­(H) bonds was not significant at an earlier time point of 75 min, where ^31^P NMR spectroscopy confirmed generation of only two products, 6% **Mo4-** *d*_**2**_ and 8% **Mo7**, supporting a concerted mechanism involving a four-membered transition state for Si–H bond cleavage. The absence of **Mo4-(Si-** *d* **)** was also confirmed by ^1^H NMR spectroscopy in the absence of the corresponding Mo–H signal.

**Scheme 4:**
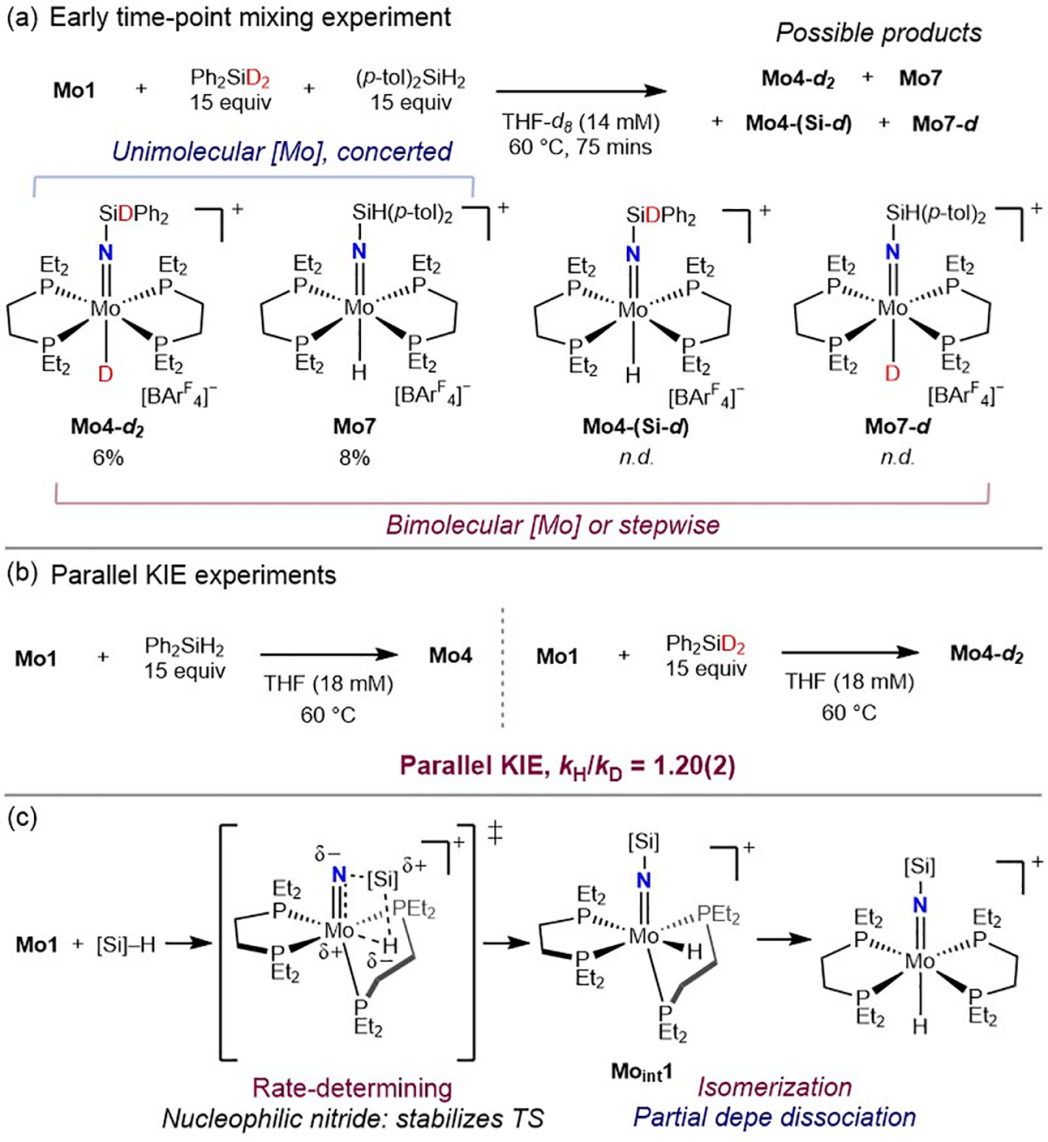
(a) Mixing Experiment with **Mo1**, Ph_2_SiD_2_, and (*p*-tol)_2_SiH_2_. (b) Parallel Deuterium KIE Experiments. (c) Proposed Mechanism

Parallel reactions of **Mo1** with Ph_2_SiH_2_ or Ph_2_SiD_2_ were conducted, and a deuterium kinetic isotope effect (KIE) of 1.20(2) was measured at 60 °C (Scheme 4b, Figure S2). The modest but primary KIE further supports an asynchronous, four-membered early transition state where Si–H bond cleavage is involved but not significant.^35–37^ From the slightly faster reaction with more electron-deficient silanes, it is likely that the nucleophilicity of the nitride plays a role in stabilizing the transition state,^22^ enabled by the polarized Si–H bond with respect to the Mo–N bond along an asynchronous Si–H activation pathway. Subsequent reduction of the imido to an amido ligand was not observed with either Ph_2_SiH_2_ and PhSiH_3_, even upon heating to 100 °C. This lack of reactivity is likely a result of the reduced nucleophilicity of the linear imido group^25,38^ or steric hindrance from the silane group attached to the nitrogen. Notably, the nucleophilicity of the nitride has been posited as important for the activation of H_2_.^39,40^ A proposed mechanism for silane addition is presented in Scheme 4c and involves the isomerization from **Mo**_**int**_ **1** to the product, likely relying on dissociation of one of the phosphines.^20^ Consistent with this proposal, the rate of the reaction exhibited a first-order dependence on silane concentration, supporting a rate-determining step that involves one equivalent of silane and one equivalent of the molybdenum nitride (Figure S3).

To further evaluate this mechanistic proposal, the hydrosilylation of a molybdenum nitride supported by 1,2-bis­(diethylphosphino)­benzene (**depBz**), **Mo8**,^20^ was studied (Scheme 5). The reaction of **Mo8** with PhSiH_3_ was demonstrably slower than the corresponding hydrosilylation of **Mo1**, with no detectable conversion at room temperature and requiring heating to 60 °C for the generation of **Mo9**. The secondary silane, Ph_2_SiH_2_ provided only trace conversion at 60 °C. The slower reactivity of **Mo8** is attributed to a higher barrier for the isomerization from the *cis*-imido molybdenum hydrido intermediate that resembles **Mo**_**int**_ **1** to the *trans*-product, a result of the more rigid **depBz** ligand as proposed in the previous study on photodriven hydrogenation.^20^ A less nucleophilic nitride with less electron-donating ability from the ligand that destabilizes the transition state for Si–H bond addition is also possible and cannot be ruled out. Interestingly, the slower hydrosilylation of **Mo8** compared to **Mo1** correlates with the sluggish reactivity of **Mo8** toward photochemical hydrogenation compared to **Mo1**,^20^ implying similar reactivity between hydrosilylation and the initial H_2_ addition during the hydrogenation.

**Scheme 5:**
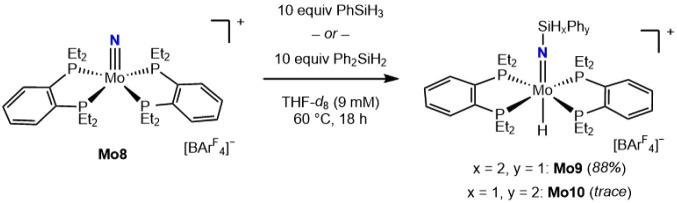
Hydrosilylation of **Mo8** with PhSiH_3_

### Photochemical Hydrogenation of Imido Molybdenum Hydride Complexes

The photochemical hydrogenation of the silyl imido molybdenum hydride compounds was examined to explore their relevance to the synthesis of free amines (Scheme 6).^20^ Irradiation of a 20 mM solution of **Mo4** in THF under 4 atm H_2_ with blue LEDs (broad band, λ_max_ = 430, 460 nm)^41^ in the absence of a photocatalyst resulted in the formation of **Mo2** with a 76% NMR yield as judged by single-scan ^31^P NMR spectroscopy relative to the PPh_3_ as an internal standard and >95% conversion of **Mo4**. This observation establishes the reactivity of the silylimido ligand in **Mo4** toward H_2_ and its intermediacy in the photochemical hydrogenation to free amine. Successful hydrogenation in the absence of a photocatalyst supports the hypothesis that the added photocatalyst in the hydrogenation of **Mo1** is needed to promote the initial addition of H_2_ to overcome the endergonic first step of the overall process.

**Scheme 6:**
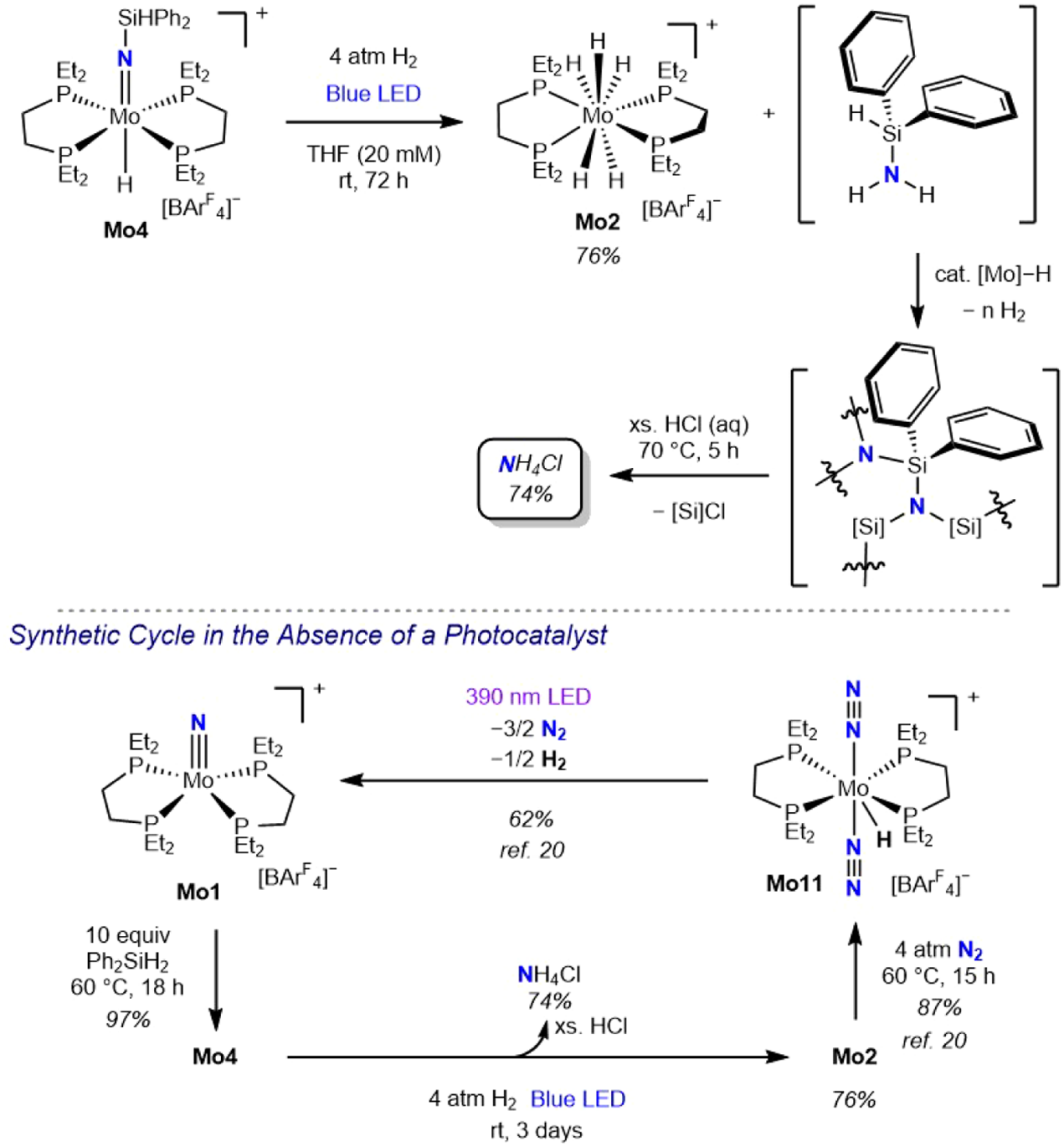
Photochemical Hydrogenation of **Mo4** and Synthetic Cycle for Ammonia Synthesis in the Absence of Precious Metal Photocatalysts

Direct spectroscopic characterization of the nitrogen-containing product following the hydrogenation of **Mo1** proved challenging, likely a result of the known dehydrogenative polymerization of silylamines promoted by transition metals.^42,43^ Analysis by ^1^H NMR and FT-IR spectroscopy did not provide evidence of Si–H and N–H bond formation. Repeating the procedure with 4 atm D_2_ furnished **Mo2-D** in 74% yield, and analysis by ^2^H NMR spectroscopy of the unpurified mixture again provided no direct evidence for the formation of Si–D and N–D bonds.

Treatment of the pentane extract of the reaction mixture with HCl following photochemical hydrogenation proved to be more successful. The volatile components of the reaction were separated from the metal complexes by vacuum transfer, and the resulting crude residue was extracted using pentane to exclude any transition metal products, and aqueous HCl was added, followed by heating to 70 °C for 18 h. This procedure yielded 74% of NH_4_Cl and resulted in hydrolysis of the N–Si bond.^44^ The yield of the ammonium chloride is consistent with the value observed for **Mo2**, and the overall transformation is a rare example of ammonia surrogate synthesis from the hydrogenation of N_2_-derived transition metal complexes without the need for external catalysts or additives.^7–9^ Performing the hydrogenation in the absence of blue light irradiation at both room temperature and 60 °C produced only trace conversion of **Mo4** with no evidence for **Mo2** or NH_4_Cl following treatment with HCl. Irradiation with blue light in the absence of H_2_ also produced a trace conversion of **Mo4**, supporting the role of H_2_ as an essential reductant. This allowed the demonstration of the ammonia synthetic cycle without the use of a photocatalyst, with steps involving the previously reported recovery of **Mo2** to **Mo1** through [(depe)_2_Mo­(N_2_)_2_H]­[BAr^F^_4_] (**Mo11**).^20^

To further explore the role of the imido and the hydride ligands in the photodriven hydrogenation to amines, a series of cationic molybdenum parent imido complexes was prepared where the X-type ligand *trans* to the Mo=NH was systematically varied. Examples include fluoride (**Mo12**),^20^ chloride (**Mo13**),^31^ and methoxide (**Mo14**)^45,46^ derivatives along with the silyl imido molybdenum chloride (**Mo15**) (Scheme 7). Each complex was subjected to standard photochemical hydrogenation conditions (blue light irradiation, 20 mM solution in THF, 4 atm H_2_, 72 h at 23 °C), and no detectable formation of **Mo2** was observed. These observations support the role of molybdenum hydride in photodriven ammonia synthesis. Likewise, **Mo15** was also unreactive under these conditions, demonstrating that the presence of the silylimido alone is insufficient to promote subsequent hydrogenation.

**Scheme 7:**
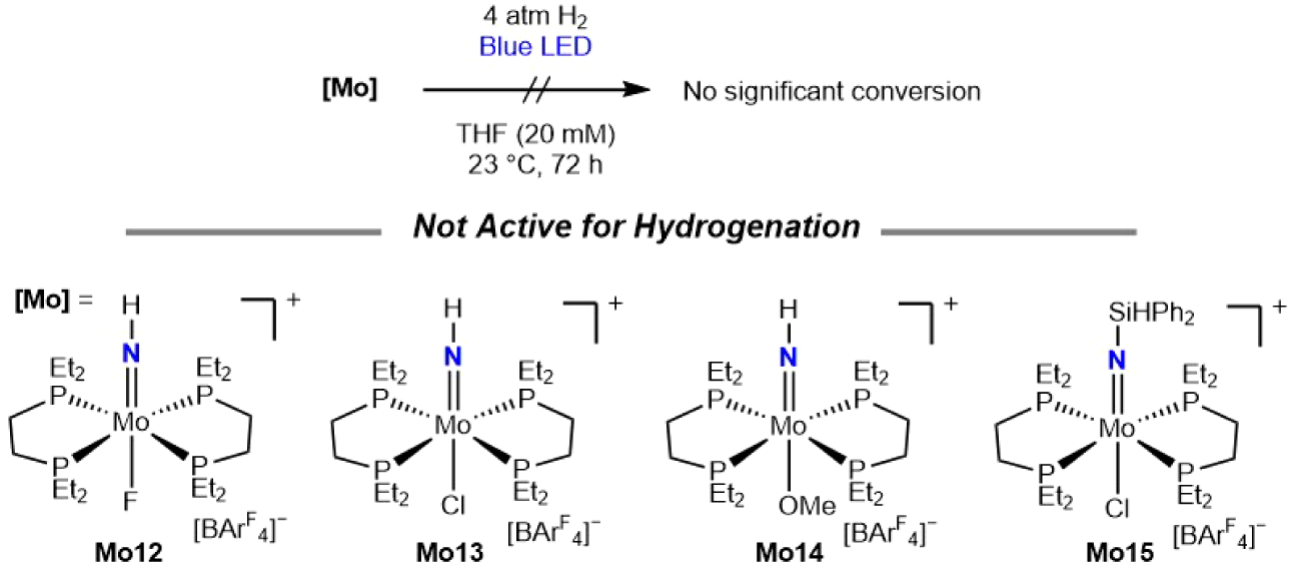
Photochemical Hydrogenation of **Mo12**–**Mo15**

Changing the phosphine ligand from **depe** to **depBz** slowed both the hydrosilylation and the hydrogenation of the molybdenum nitride complexes.^20^ To determine whether this ligand effect extends to the subsequent hydrogenation step, the **depBz**-supported molybdenum silylimido hydride, **Mo9**, was subjected to photochemical hydrogenation (Scheme 8). Notably, **Mo16** was obtained in 84% yield, along with 86% NH_4_Cl following protonolysis, indicating that once the molybdenum imido hydride is formed, the identity of the bis­(phosphine) ligand (**depe** vs **depBz**) does not significantly influence the efficiency of the hydrogenation step.

**Scheme 8:**
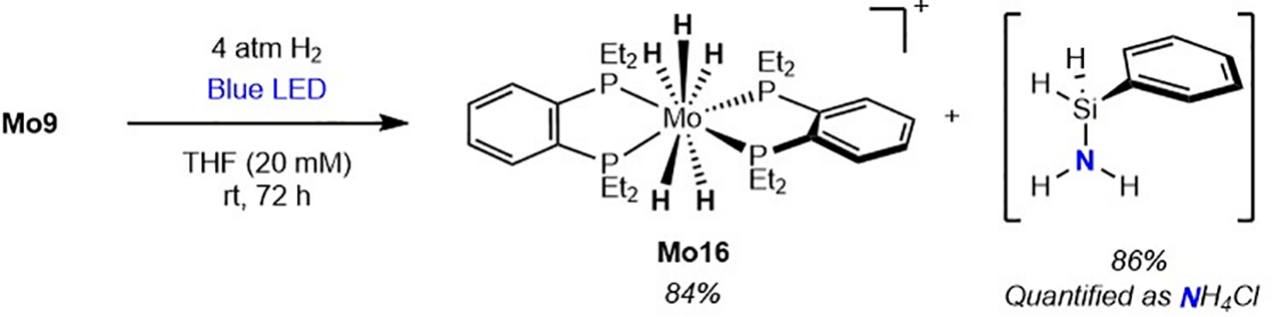
Photochemical Hydrogenation of **Mo9**

### Additional Mechanistic Investigations for the Photochemical Hydrogenation

The observation of ammonia formation following the photodriven hydrogenation of **Mo4** motivated additional mechanistic investigations. The electronic absorption spectrum of **Mo4** in THF exhibited two major bands at λ_max_ = 496 nm (ε_496_ ∼ 205 L mol^–1^ cm^–1^) and λ_max_ = 329 nm (ε_496_ ∼ 2942 L mol^–1^ cm^–1^). Time-dependent density functional theory (TD-DFT) calculations aided the assignment of these transitions from a metal-centered nonbonding orbital to a delocalized π* orbital of the molybdenum imido and from a delocalized σ orbital of Mo–H to the same delocalized π* orbital of the Mo=N bond, respectively. These assignments suggest that irradiation with blue light weakens the Mo=N bond rather than promotes Mo–H bond homolysis. Therefore, a pathway involving the intermediate, **Mo**_**int**_ **2**, followed by radical rebound to the imido nitrogen to generate **Mo**_**int**_ **3** is less likely (Scheme 9a). Interestingly, irradiation of a THF-*d*_8_ solution of **Mo4** with 390 nm LED light, in both the presence and absence of H_2_, generated **Mo1** in near-quantitative yield over 18 h with unidentified silicon products. These results support an alternative pathway that is operative under higher-energy light. This result is consistent with the unproductive photochemical hydrogenation of **Mo1** in the presence of 2.5 mol % of *fac*-Ir­(ppy)_3_ with 390 nm light, where only trace conversion was observed as the imido molybdenum hydride intermediate, **Mo3**, likely reverts to **Mo1**.

**Scheme 9:**
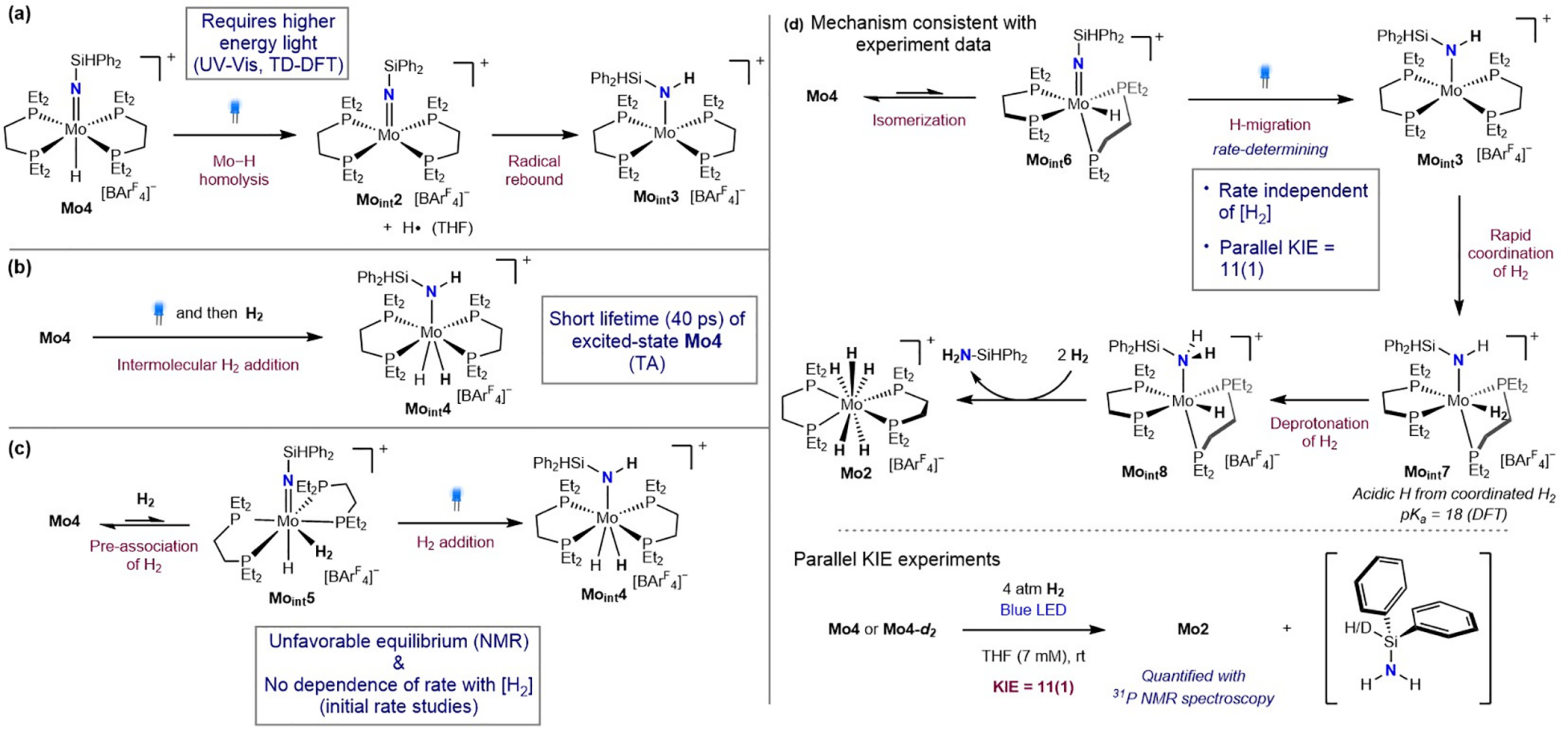
Proposed Mechanisms for the Photochemical Hydrogenation of **Mo4** and Mechanistic Investigations. (a) Mechanism Involving Mo–H Bond Homolysis. (b) Mechanism Involving Photoactivation of **Mo4** Followed by Intermolecular H_2_ Addition. (c) Mechanism Involving Preassociation of H_2_ Followed by Addition to Mo=N Bond. (d) Mechanism Involving Intramolecular H-Migration

Transient absorption spectroscopy (TA) of **Mo4** with λ_pump_ = 490 nm established a relatively short excited-state lifetime (τ) of 40 ps at room temperature, supporting a unimolecular transformation of excited-state **Mo4**.^47^ This observation suggests that transformations involving bimolecular interactions of photoexcited **Mo4**, such as reaction with H_2_ to form an intermediate (**Mo**_**int**_ **4**) or with other molybdenum compounds, are likely inoperative (Scheme 9b). No H/D exchange at the Mo–H bond of **Mo4** was observed upon addition of 4 atm of D_2_ in the presence and absence of light. Therefore, the formation of a molybdenum dihydrogen complex (**Mo**_**int**_ **5**) prior to excitation also appears unfavorable (Scheme 9c), where the association of D_2_ would be expected to promote H/D exchange.^48–50^ No change in chemical shift or peak broadening of the H_2_ signal was observed in the^ 1^H NMR spectra upon addition of **Mo4**, nor were changes in the ^31^P NMR signal of **Mo4** observed upon addition of H_2_, indicating a nonexisting or unfavorable equilibrium for the H_2_ association, providing additional support for this hypothesis.

The influence of the H_2_ pressure on the rate of the photodriven hydrogenation of **Mo4** was also investigated. Four J. Young NMR tubes containing 5.7 mM solutions of **Mo4** in THF-*d*_*8*_ were charged with 0.5, 1, 2, or 4 atm of H_2_ and were irradiated with blue light. Each reaction was monitored by ^1^H NMR spectroscopy, and the diagnostic Mo–H resonances for **Mo2** were integrated versus an internal mesitylene standard. The effective concentration of H_2_ at each pressure was also determined independently by quantitative ^1^H NMR spectroscopy and yielded values of 2.0, 4.2, 5.6, and 12.5 mM, respectively. Despite the significant differences in [H_2_], no clear variance in the initial hydrogenation rates was observed (Figure S4). Minor rate fluctuations were attributed to experimental variations arising from slight differences in the LED proximity or glassware transmittance. These results demonstrate that the rate of photochemical hydrogenation of **Mo4** is independent of H_2_ concentration, providing additional evidence against a pathway involving preassociation of dihydrogen.

Based on these observations, an alternative pathway involving intramolecular H-migration from Mo to N following isomerization to the intermediate with *cis*-geometry (**Mo**_**int**_ **6**) is proposed (Scheme 9d). This mechanism involves the formation of **Mo**_**int**_ **3** as an intermediate, and the subsequent intramolecular H-migration is likely the rate-determining step. This conclusion is supported by the absence of the rate dependence on [H_2_] and the failure to detect molybdenum-containing intermediates by either ^31^P or ^1^H NMR spectroscopy. To further support this hypothesis, parallel deuterium KIE experiments were conducted for the photochemical hydrogenation of **Mo4** and **Mo4-** *d*_**2**_ and a KIE value of 11(1) was measured, supporting Mo–H bond cleavage in the rate-determining step (Figure S5). Large KIE values over 10 have precedent, including intramolecular H-migration from osmium to nitrile nitrogen, where the magnitude has been attributed to tunneling effects.^51^ Similarly high KIEs have also been reported for intermolecular protonolysis and for both intra- and intermolecular C–H bond activations, where the observed values arise from compound effects involving multiple reaction steps, contributions from several vibrational or rotational modes, or the presence of a geometrically or energetically symmetric transition state.^52^ The relatively high energy barrier associated with this transformation without light irradiation showed no hydrogenation from the ground state, which is attributed to the polarity inversion of H from Mo–H to N–H, a process that is disfavored due to the modest nucleophilicity of the imido nitrogen.^25,38–40^ The role of visible light in this context is to facilitate the reaction by inducing bond weakening of the imido ligand through population of the π* orbital of the Mo = N bond, thereby enabling the system to overcome the energetic barrier.

The intermediate, **Mo**_**int**_ **3**, is a five-coordinate, 14-electron molybdenum complex bearing an amido ligand with a weaker *trans*-effect compared to nitride or imido ligands.^53^ This likely enables **Mo**_**int**_ **3** to associate with H_2_. Attempts to trap **Mo**_**int**_ **3** with L-type ligands such as *tert*-butyl cyanide (^t^BuCN) have been unsuccessful, as reversion to the *trans*-^t^BuCN-substituted molybdenum nitride complex was observed upon irradiation with blue light in the absence of H_2_. It is proposed that one of the protons from the coordinated H_2_ in **Mo**_**int**_ **7** is sufficiently acidic to protonate the amido ligand, consistent with previous reports with ruthenium dihydrogen complexes.^16^ Subsequent ligand exchange from the coordinated amine to H_2_ completes the transformation, yielding **Mo2** and a free amine as the final products. Based on this proposal, the previously noted stage-specific ligand dependency in the hydrosilylation and the following hydrogenation step using the **depBz** ligand likely originates either from the comparatively reduced influence of ligand rigidity on ligand reorganization or from the diminished role of imido nitrogen nucleophilicity in the 1,2-H migration step during the photochemical hydrogenation of **Mo9**.

The proposed pathway contrasts the hydrogenation of **Mo1**, where the reaction under reduced H_2_ pressure required an extended period to reach completion,^20^ and only trace products were observed when the reaction was conducted in a J. Young NMR tube in the absence of vigorous stirring. This observation implies a positive dependence of the reaction rate on [H_2_]. These results suggest that in the hydrogenation of **Mo1**, the initial H_2_ addition is likely an intermolecular transformation involving the excited-state **Mo1** with H_2_ and is rate-limiting. Therefore, the photocatalyst likely promotes the generation of the triplet-excited state of **Mo1** (^**T**^ **Mo1**) through energy transfer^20,54^ and exploiting the longer lifetime.^55^

## Conclusion

The synthesis of bis­(phosphine) silyl­(imido) molybdenum hydrides has been accomplished from the addition of free silanes to the corresponding N_2_-derived molybdenum nitride. These compounds are surrogates for the parent imido molybdenum hydrides that are likely formed during the photodriven hydrogenation of N_2_ to ammonia. Notably, the subsequent hydrogenation of silyl­(imido) molybdenum hydrides was accomplished using blue light irradiation in the presence of 4 atm of H_2_ with no precious metal photocatalyst. Deuterium labeling and relative rate studies of the initial thermal hydrosilylation step support a concerted, four-membered transition state for Si–H bond addition, where the nucleophilicity of the nitride stabilizes the transition state. Additional mechanistic studies on the photodriven hydrogenation to the corresponding molybdenum hydride and free amine support a pathway involving rate-determining migration of the Mo–H to the imido nitrogen. Changing the phosphine from **depe** to **depBz** slowed the thermal hydrosilylation of the molybdenum nitride and net photochemical hydrogenation to yield free ammonia. In contrast, there was a negligible effect on the subsequent photochemical hydrogenation of the silylimido molybdenum hydride, suggesting minimal reorganization or phosphine dissociation en route to cleaving the metal–nitrogen bond.

## Supplementary Material



## Notes

General considerations; preparation of molybdenum complexes; mechanistic investigations for the hydrosilylation; photodriven hydrogenations; spectroscopic data; computational data; X-ray crystallographic data; and references (PDF)
