# Supplementary material for: Hydrosilylation of a Molecular Molybdenum Nitride Provides Mechanistic Insights into Photodriven Ammonia Synthesis from N2 and H2
Source: J Am Chem Soc. 2026 Feb 10;148(7):7761–71. doi: 10.1021/jacs.5c22220 (PMC12951431; doi:10.1021/jacs.5c22220)
Supplement: Supplementary file 1 [file ja5c22220_si_001.pdf]

Supporting Information

Hydrosilylation of a Molecular Molybdenum Nitride Provides Mechanistic Insights into Photodriven Ammonia Synthesis from N_2 and H_2

*Junho Kim, Nidhi Kaul, Matthew V. Pecoraro and Paul J. Chirik**

Department of Chemistry, Frick Laboratory
Princeton University, Princeton, NJ 08544, USA

pchirik@princeton.edu

Table of Contents

I. General Considerations S2

II. Preparation of Molybdenum Complexes S7

III. Mechanistic Investigations for the Hydrosilylation S11

IV. Photodriven Hydrogenation Reactions S15

V. Spectroscopic Data S20

VI. Computational Data S73

VI. X-Ray Crystallographic Data S79

VII. References S85

I. General Considerations

All air- and moisture-sensitive manipulations were carried out using vacuum line, Schlenk and cannula techniques or in an MBraun inert atmosphere nitrogen dry box unless otherwise noted. All glassware was stored in a pre-heated oven (≥ 150° C) prior to use. The solvents used for air- and moisture-sensitive manipulations were dried and deoxygenated using literature procedures.[1] Benzene-d_6 used for NMR spectroscopy was distilled from sodium metal and stored over 4 Å molecular sieves. THF-d_8 used for NMR spectroscopy was dried using sodium-benzophenone ketyl[2] and directly vacuum transferred to the reaction mixtures prior to use. Celite, alumina, and silica were dried at 180 ºC under vacuum for 3 days prior to use in the glovebox.

All solid and liquid-phase chemicals were purchased from Sigma Aldrich. Solid reagents were dried under vacuum overnight and stored under dinitrogen prior to use. Liquid reagents were dried over 4 Å molecular sieves or CaH_2 and degassed by three freeze-pump-thaw cycles. The following compounds were prepared according to literature procedures: **Mo1**,[3] **Mo8,**[4] **Mo12**[4], **(depe)$_2$Mo(N)(Cl)**[5] and (*p*-tol)$_2$SiH$_2$.[6]

Glass vessels were silylated for use in photocatalytic reactions according to the following procedure: The glass vessel was acid-leached in HNO_3 for 24 h, rinsed with 3 times with water distilled in glass and dried overnight at 60 °C. A 5 wt % solution of dichlorodimethylsilane in dry toluene was added to the glass vessel and the vessel was rinsed with dry toluene following with dry methanol. The glassware was dried in 180 °C oven to complete the silylation. Kessil® LED lamps are used for light irradiation. Specific models include H150-Blue Kessil® lamps for blue LED, PR160-440 nm Kessil® lamps for 440 nm LED and PR160-390 nm Kessil® lamps for 390 nm LED.

H_2 and N_2 gas were purchased from Airgas®, and passed through a column of alternating layers of 4 Å molecular sieves and MnO/Vermiculite before being introduced to glassware. Anhydrous ammonia was prepared via ammonia condensation onto sodium metal, forming an amber electrolyte and was subjected to three freeze pump-thaw cycles before use. When using

the sodium electrolyte method to dry ammonia, use small quantities with **extreme caution** to avoid violent H_2(g) evolution from the reaction of sodium metal with residual water in ammonia. When storing the ammonia electrolyte in a closed vessel, regularly expose the mixture to freeze pump thaw cycles to avoid pressure buildup over time.

^{1}H NMR spectra were recorded on either Bruker AVANCE 300, 400 or 500 spectrophotometers operating at 300.13 MHz, 399.8 MHz and 500.46 MHz, respectively. ^{13}C NMR spectra were recorded on either Bruker Avance 300, 400 or 500 spectrometers operating at 75.48 MHz, 100.54 MHz and 125.85 MHz, respectively. All ^{1}H and ^{13}C NMR chemical shifts are reported in ppm relative to $SiMe_4$ using the ^{1}H and ^{13}C chemical shifts of the solvent[7] as a standard. ^{1}H NMR data for diamagnetic compounds are reported as follows: chemical shift, multiplicity (s = singlet, d = doublet, t = triplet, q = quartet, p = pentet, br = broad, m = multiplet, app = apparent, obsc = obscured), coupling constants (Hz), integration, assignment. ^{13}C NMR data for diamagnetic compounds are reported as follows: chemical shift, number of protons attached to carbon (e.g. CH_2), assignment. ^{2}H NMR spectra were recorded on Bruker Avance 400 or 500 spectrometers operating at 61.42 MHz and 76.88 MHz, respectively and referenced to TMS-d_{12} as an external standard. ^{19}F NMR spectra were recorded on Bruker Avance 400 or 500 spectrometers operating at 376.19 MHz and 470.96 MHZ, respectively, and referenced to $CFCl_3$ as an external standard. ^{31}P NMR spectra were recorded on either Bruker Avance 400 or 500 spectrometers operating at 161.84 MHz and 202.00 MHz, respectively, and were referenced to 85% H_3PO_4 as an external standard.

Infrared spectroscopy was conducted on a Thermo-Nicolet iS10 FT-IR spectrometer calibrated with a polystyrene standard. Absorption spectra were recorded on a Cary 60 UV-Vis spectrometer (Agilent Technologies, Santa Clara, California). Excitation spectra were recorded on a QuantaMaster 400 (HORIBA Scientific, NJ). Measurements were done using a 1 cm cuvette fitted with a J. Young valve and sealed with a Teflon cap prepared in an N_2-filling glove box. For transient absorption measurements within time window of 0-7 ns (referred as Helios

measurement), a 1 kHz regeneratively amplified Ti:Sapphire laser (Coherent Libra, Santa Clara, California) with a commercial optical parametric amplifier (OPerA Solo, Vilnius, Lithuania) and commercial transient absorption spectrometer (Ultrafast Systems Helios, Sarasota, Florida) were used with a detailed setup description reported previously[8]. In brief, the output pulse of the Ti:Sapphire laser Coherent Libra, centered at 800 nm with a duration of ca. 45 fs and pulse power of 4 W, is split with a 90-10 (r-t) beamsplitter to generate the pump and probe. The reflected portion of the output 800 nm is directed into the commercial optical parametric amplifier OperA Solo to generate light at the desired pump wavelength. Both the output of the OperA Solo and transmitted portion of the output 800 nm are directed into a commercial transient absorption spectrometer Helios. The pump pulse is chopped at 500 Hz with its polarization controlled by a broadband λ/2 waveplate and then focused into the sample position. The remaining 800 nm is focused into a 2 mm thick Al_2O_3 crystal to generate a white light continuum from 430 nm to 800 nm or a translating CaF_2 crystal for white light between 300 nm to 700 nm with its intensity and polarization controlled by a combination of λ/2 waveplate and polarizer. After filtering the remaining fundamental light, the continuum is tuned to overlap with pump pulse and focused into the sample position and eventually enters a CCD camera for detection. All experiments on this setup were done at magic angle[9] to avoid reorientation effects of the samples on nanosecond timescale. For transient absorption measurements within time window of 0.001-500 μs (referred as EOS measurement), a 1 kHz regeneratively amplified Ti:Sapphire laser (Coherent Libra, Santa Clara, California) with a commercial optical parametric amplifier (OPerA Solo, Vilnius, Lithuania) and commercial transient absorption spectrometer (Ultrafast Systems Helios, Sarasota, Florida) were used. In brief, the output pulse of the Ti:Sapphire laser Coherent Libra, centered at 800 nm with a duration of ca. 45 fs and pulse power of 4 W, is split with a 90-10 (r-t) beamsplitter to generate an unused pump and a used probe source. For these experiments the pump is generated by an Ekspla PT403. Both the output of the Ekspla PT403 and transmitted portion of the output 800 nm are directed into a commercial transient absorption spectrometer Helios. The

pump pulse is focused into the sample position. The remaining 800 nm is focused into a 2 mm thick Al_2O_3 crystal to generate a white light continuum from 430 nm to 800 nm or a translating CaF_2 crystal for white light between 300 nm to 700 nm with its intensity and polarization controlled by a combination of λ/2 waveplate and polarizer. After filtering the remaining fundamental light, the continuum is tuned to overlap with pump pulse and focused into the sample position and eventually enters a CCD camera for detection.

Single crystals suitable for X-ray diffraction were coated with polyisobutylene oil in the drybox, transferred to a nylon loop and then quickly transferred to either the goniometer head of a diffractometer equipped with a Bruker PHOTON III detector and Cu X-Ray tube (λ = 1.54178 Å) or a Rigaku XtaLAB Synergy-i equipped with a Mo X-ray tube (λ = 0.71073 Å) and a Cu X-ray tube (λ = 1.54178 Å). Preliminary data revealed the crystal system. The data collection strategy was optimized for completeness and redundancy using either the Bruker APEXII software suite or Rigaku CrysAlis^{Pro} software suite. The space group was identified, and the data were processed and corrected for absorption. The structures were solved using intrinsic phasing (SHELXT) and completed by subsequent Fourier synthesis and refined by full-matrix least-squares procedures in Olex2. Unless otherwise specified, hydrogen atoms were modelled as riding atoms.

All DFT calculations were performed with the ORCA 5.0.3 program package.[10] Geometry optimizations and single-point calculations were carried out at the M06L level of DFT.[11] Time-dependent DFT (TD-DFT) calculations were carried out at the TPSSh level of DFT.[12] Alrichs' all-electron Gaussian basis sets were employed for all calculations,[13,14,15] wherein the triple-ζ basis set ZORA-def2-TZVP, which includes one set of polarization functions, was used to describe all atoms except molybdenum.[15] The triple-ζ basis set SARC-ZORA-TZVP, which includes one set of polarization functions, was used to describe molybdenum atom.[16] SARC/J was chosen as an auxiliary basis sets to match the orbital basis.[17,18] The RIJCOSX approximation was used to accelerate the calculations.[19,20,21] Solvation calculations were carried out at the same level of

theory as the single point calculations by employing conductor-like PCM (cPCM) model with the dielectric constants of ε = 7.43 for THF.[22,23]

II. Preparation of Molybdenum Complexes.

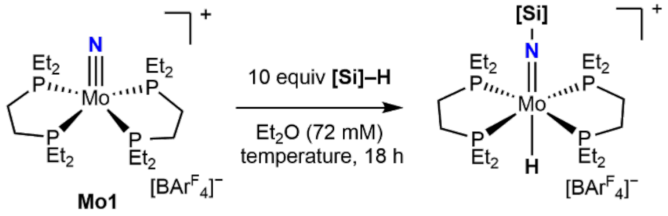

General Procedure for the Synthesis of Molybdenum Silyl Imido Hydrides (Mo4, Mo5, Mo9). In a nitrogen-filled glovebox, a J. Young NMR tube was charged with 50 mg (0.036 mmol, 1.0 equiv) of **Mo1**, 10.0 equiv of silane and approximately 0.5 mL of Et_2O. The NMR tube was sealed and removed from the glovebox. The mixture was heated at 60 ºC for 18 hours and a color change from red to a dark violet was observed. The progress of the reaction was monitored using ^{31}P NMR spectroscopy. After cooling to room temperature, the reaction was brought into the glovebox and filtered through a pad of Celite on a glass frit. The solvent was removed *in vacuo* and the resulting viscous liquid was triturated with pentane (3 x 5 mL). The resulting violet solid was transferred to a 20 mL scintillation vial using ~2 mL of Et_2O. Approximately 15 mL of pentane was slowly layered on the top and recrystallization at room temperature overnight followed by washing with pentane (3 x 5 mL) and drying *in vacuo* yielded the desired product as a violet solid.

Mo4: 60 °C, 97% isolated yield. **^{1}H NMR** (400 MHz, THF-d_8) δ 7.78 (s, 8H, BAr^F_4), 7.57 (s, 4H, BAr^F_4), 7.55 – 7.51 (m, 4H, Si–*α*,*β*-C_6*H_5*), 7.46 – 7.39 (m, 4H, Si–*α*,*β*-C_6*H_5*), 7.36 (tt, *J* = 7.8, 6.3 Hz, 2H, Si–*γ*-C_6*H_5*), 5.12 (s, 1H, Si–*H*), 2.17 – 2.02 (m, 4H, depe), 2.00 – 1.84 (m, 4H, depe), 2.17 – 2.02 (m, 16H, depe), 1.57 – 1.49 (m, 4H, depe), 1.11 (t, *J* = 6.5 Hz, 12H, depe), 1.03 (t, *J* = 6.5 Hz, 12H, depe), –6.88 (p, *J* = 40.1 Hz, 1H, Mo–*H*). **^{13}C{^{1}H} NMR** (125.85 MHz, THF-d_8) δ 135.6 (BAr^F_4), 135.5 (br, Si-*C_6*H_5), 134.9 (s, BAr^F_4), 131.27 (Si-*C_6*H_5), 128.9 (Si-*C_6*H_5), 118.1 (BAr^F_4), 24.2 (m, P(*C*H_2)$_2$P), 23.9 (m, P*C*$H_2$$CH_3$), 9.5 (P$CH_2$*C*$H_3$), 8.2 (P$CH_2$*C*$H_3$). **^{19}F NMR** (376.19 MHz, THF-d_8): δ –63.4, **^{31}P{^{1}H} NMR** (161.84 MHz, THF-d_8): δ 65.8.

Mo5: room temperature, 95% isolated yield. **^{1}H NMR** (400 MHz, THF-d_8) δ 7.87 – 7.75 (m, 8H, BAr^F_4), 7.57 (s, 4H, BAr^F_4), 7.50 – 7.46 (m, 2H, Si–*α*,*β*-C_6*H_5*), 7.44 – 7.40 (m, 1H, Si–*γ* -C_6*H_5*),

7.38 – 7.33 (m, 2H, Si– *α,β*-C_6H_5), 4.66 (s, 2H, Si–*H*), 2.19 – 2.06 (m, 4H, depe), 2.06 – 1.83 (m, 16H, depe), 1.67 – 1.52 (m, 4H, depe), 1.12 (t, *J* = 6.7 Hz, 12H, depe), 1.05 (t, *J* = 6.5 Hz, 12H, depe), –6.80 (p, *J* = 39.2 Hz, 1H, Mo–*H*). **$^{13}C\{^1H\}$ NMR** (125.85 MHz, THF-d_8) δ 163.0 (dd, *J* = 99.6, 49.8 Hz, BAr^F_4), 236.1 (Si-C_6H_5), 135.8 (BAr^F_4), 131.7 (BAr^F_4), 130.5 – 129.5 (Si-C_6H_5), 129.3 (BAr^F_4), 124.4 (Si-C_6H_5), 118.6 – 118.1 (Si-C_6H_5), 26.1 – 25.5 (m, $P(CH_2)_2P$), 24.7 – 24.2 (m, PCH_2CH_3), 9.6 (PCH_2CH_3), 8.3 (PCH_2CH_3). **^{19}F NMR** (376.19 MHz, THF-d_8): δ –63.4.
$^{31}P\{^1H\}$ NMR (161.84 MHz, THF-d_8): δ 66.8.

Mo9: 60 °C, 93% isolated yield. **^1H NMR** (400 MHz, THF-d_8) δ 8.12 (dd, *J* = 6.4, 3.2 Hz, 4H, BAr^F_4), 7.82 (d, *J* = 3.2 Hz, 8H, BAr^F_4), 7.79 (d, *J* = 3.7 Hz, 4H, PC_6H_4P), 7.60 (br, 4H, PC_6H_4P), 7.35 (tt, *J* = 6.7, 2.2 Hz, 1H, Si– *γ*-C_6H_5), 7.25 – 7.15 (m, 4H, Si–*α,β*-C_6H_5) 4.59 (s, 2H, Si–*H*), 2.67 – 2.53 (m, 4H, depe), 2.46 (tt, *J* = 7.4, 3.7 Hz, 4H, depe), 2.37 – 2.27 (m, 4H, depe), 2.03 (dq, *J* = 14.9, 7.6 Hz, 4H, depe), 1.41 – 1.32 (m, *4*H, depe), 1.12 (t, *J* = 6.6 Hz, 12H, depe), 0.70 (t, *J* = 6.7 Hz, 12H, depe), –6.57 (p, *J* = 41.0 Hz, 1H, Mo–*H*). **^{19}F NMR** (376.19 MHz, THF-d_8): δ –63.5. **$^{31}P\{^1H\}$ NMR** (161.84 MHz, THF-d_8): δ 71.7.

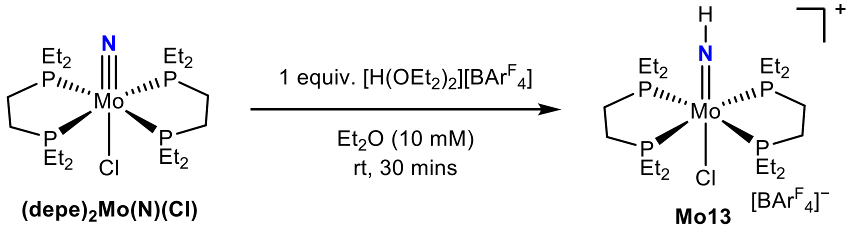

Preparation of Mo13. In a nitrogen-filled glovebox, a 20 mL scintillation vial equipped with a magnetic stir bar was charged with 11 mg (0.02 mmol, 1.0 equiv) of **(depe)$_2$Mo(N)(Cl)** and approximately 1 mL of Et_2O. Another 20 mL scintillation vial was charged with 20 mg (0. 02 mmol, 1.0 equiv) of $[H(OEt_2)_2][BAr^F_4]$ and approximately 1 mL of Et_2O. The solution was added to the stirring solution of **(depe)$_2$Mo(N)(Cl)** dropwise and a color change from pale yellow to red was observed. After stirring for 30 min, the crude mixture was filtered through a pad of Celite on a glass frit. The solvent was removed *in vacuo* and the resulting red solid was transferred to a 20

mL scintillation vial using ~2 mL of Et_2O. Approximately 15 mL of pentane was slowly layered on the top and recrystallization at room temperature overnight followed by washing with pentane (3 x 5 mL) and drying *in vacuo* yielded the desired product as a red solid (96% yield). The product was characterized with ^{1}H, ^{31}P NMR spectroscopy and matches with the previously reported literature data.[5]

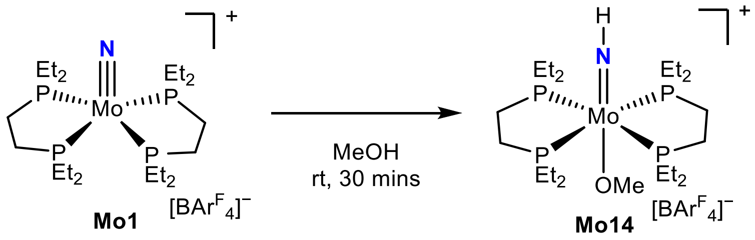

Preparation of Mo14. In a nitrogen-filled glovebox, a 100 mL Schlenk flask equipped with a magnetic stir bar was charged with 28 mg (0. 02 mmol) of **Mo1**. The Schlenk flask was sealed and brought out from the glovebox and attached to a Schlenk line. Under the flow of N_2, approximately 10 mL of MeOH was added and the reaction mixture was stirred for 30 min, and a color change from red to yellow was observed. After stirring for 30 mins, the solvent was removed in vacuo and the reaction vessel was brought into the glovebox. The resulting yellow solid was transferred to a 20 mL scintillation vial using ~2 mL of Et_2O. Approximately 15 mL of pentane was slowly layered and recrystallization at room temperature overnight followed by washing with pentane (3 x 5 mL) and drying *in vacuo* yielded the desired product as a yellow solid (94% yield). **^{1}H NMR** (400 MHz, THF-d_8) δ 7.78 (dd, *J* = 6.3, 3.3 Hz, 4H, $BAr^F{}_4$), 7.57 (d, *J* = 3.1 Hz, 8H, $BAr^F{}_4$), 3.03 (s, 3H, OCH_3), 2.12 – 1.74 (m, 24H, depe), 1.19 (dd, *J* = 9.9, 4.9 Hz, 24H, depe). **^{19}F NMR** (376.19 MHz, THF-d_8): δ –63.4. **^{31}P{^{1}H} NMR** (161.84 MHz, THF-d_8): δ 45.2.

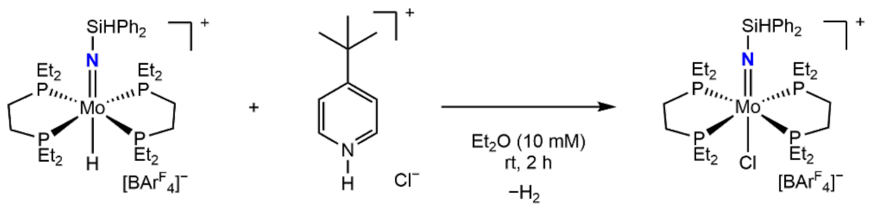

Preparation of Mo15. In a nitrogen-filled glovebox, a 20 mL scintillation vial equipped with a magnetic stir bar was charged with 30 mg (0. 02 mmol, 1.0 equiv) of **Mo4** and approximately 1 mL of Et_2O. Another 20 mL scintillation vial was charged with 3.3 mg (0. 02 mmol, 1.0 equiv) of *p*-^{t}Bupyridinium chloride and approximately 1 mL of Et_2O. The solution was added to the stirring solution of **Mo4** dropwise over a course of 15 min and a color change from violate to a pale purple was observed. After stirring for 2 hours, the crude mixture was filtered through a pad of Celite on a glass frit. The solvent was removed *in vacuo* and the resulting pale purple solid was transferred to a 20 mL scintillation vial using ~2 mL of Et_2O. Approximately 15 mL of pentane was slowly layered on the top and recrystallization at room temperature overnight followed by washing with pentane (3 x 5 mL) and drying *in vacuo* yielded the desired product as a pale purple solid (98% yield). **^{1}H NMR** (400 MHz, THF-d_8) δ 7.84 – 7.75 (m, 8H, $BAr^F{}_4$), 7.65 – 7.59 (m, 4H, Si–*α*,*β*-C_6H_5), 7.57 (s, 4H, $BAr^F{}_4$), 7.53 – 7.48 (m, 2H, Si– *γ*-C_6H_5), 7.46 – 7.42 (m, 4H, Si–*α*,*β*-C_6H_5), 7.25 – 7.15 (m, 4H, Si–*α*,*β*-C_6H_5) 4.96 (s, 1H, Si–*H*), 2.17 – 1.97 (m, 8H, depe), 1.99 – 1.78 (m, 16H, depe), 1.21 – 1.15 (m, 24H, depe). **^{19}F NMR** (376.19 MHz, THF-d_8): δ –63.4. **^{31}P{^{1}H} NMR** (161.84 MHz, THF-d_8): δ 44.8.

III. Mechanistic Investigations for the Hydrosilylation Reaction.

Competition experiment with Mo1, Ph_2SiH_2 and (*p*-tol)$_2$SiH$_2$. In a nitrogen-filled glovebox, a J. Young NMR tube was charged with 10 mg (0.0072 mmol, 1.0 equiv) of **Mo1**, 20.1 µL (10 equiv) of Ph_2SiH_2 and 23.0 µL (10.0 equiv) of (*p*-tol)$_2$SiH$_2$ and 0.55 mL of THF-d_8. The NMR tube was sealed and removed from the glovebox. The mixture was heated at 60 ºC and two products, **Mo4** and **Mo7**, were identified using ^{1}H and ^{31}P NMR spectroscopy (Table S1).

Table S1. Reaction monitoring of the competition experiment.

time (h)	**Mo4** yield (%)	**Mo7** yield (%)	Total yield (%)
0	0	0	0
1	6	4	10
2	12	10	22
3	17	13	30
4	20	17	37
5	25	19	45
6	29	23	51
7	31	25	56
8	34	27	61
9	36	29	65
10	38	31	69
11	40	33	72
12	42	34	76
14	43	37	80
16	47	38	85
20	49	40	90
24	52	42	94
30	54	43	97

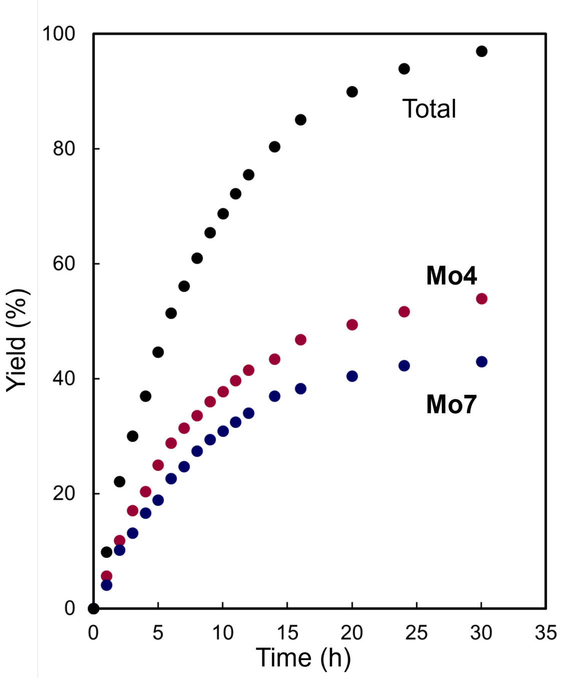

Figure S1. Reaction monitoring for a competition experiment of **Mo1** with Ph_2SiH_2 and (p-tol)$_2SiH_2$.

Measurement of Parallel Kinetic Isotope Effect. In a nitrogen-filled glovebox, a 1.0 mL volumetric flask was charged with 25 mg (0.018 mmol) of **Mo1** and THF was added to make the total volume of the solution as 1.0 mL. 0.4 mL of the stock solution was transferred into two separate J. Young NMR tubes, each charged with 15 equiv of Ph_2SiH_2 or Ph_2SiD_2 and a glass capillary charged with benzene-d_6 solution of PPh_3 as an external standard. The reaction was monitored at 60 ºC and the product was quantified using ^{31}P NMR spectroscopy using PPh_3 as an external standard (Table S2).

Table S2. Duplicated parallel KIE experiments with **Mo1** and $Ph_2SiH_2(D_2)$.

Run 1			Run 2		
time (mins)	H Yield (%)	D yield (%)	time (mins)	H Yield (%)	D yield (%)
0	0	0	0	0	0
5			5		
15			15		
20	4.8		20	4.5	
30	6.6	5.1	30	9.0	6.1

40	10.0	8.0	40	11.1	8.4
50	11.8	9.3	50	13.7	11.0
60	14.0	10.9	60	15.5	12.2
70	16.0	13.3	70	17.9	15.0
80	18.0	14.5	80	20.0	16.7
90		17.1	90		19.4
100		19.0	100		
KIE = 1.2			KIE = 1.2		

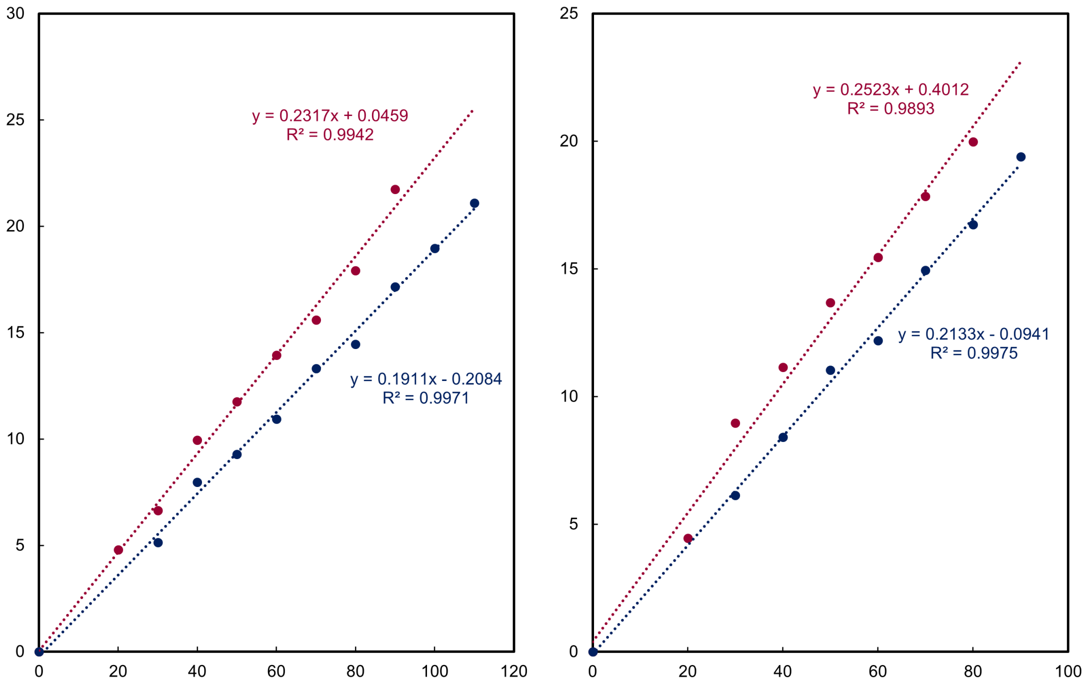

Figure S2. Reaction monitoring of the parallel KIE experiment with **Mo1** and $Ph_2SiH_2(D_2)$. Left: 1st run, right: 2nd run. X-axis denotes time (mins) and y-axis denotes yield (%). Maroon denotes the reaction with Ph_2SiH_2 and dark blue denotes the reaction with Ph_2SiD_2.

Measurement of Reaction Order in Diphenylsilane. In a nitrogen-filled glovebox, a scintillation vial was charged with 30 mg (0.022 mmol) of **Mo1** and THF-d_8 was added to make the total volume of the solution 1.2 mL. 0.4 mL of the stock solution was transferred into three separate J. Young NMR tubes, charged with 4.8, 13, or 21.8 equiv of Ph_2SiH_2 and a glass capillary charged with benzene-d_6 solution of PPh_3 as an external standard. The reaction was monitored at 60 ºC

and the product was quantified using ^{31}P NMR spectroscopy using PPh_3 as an external standard (Table S3)

Table S4. Kinetics experiment for rate dependence on [Ph_2SiH_2].

**4.80 equiv**		**13.04 equiv**		**21.82 equiv**	
time (mins)	Yield (%)	time (mins)	Yield (%)	time (mins)	Yield (%)
17.4	1.5	0.0	0.9	0.0	0.0
27.4	2.5	3.0	1.1	2.8	2.1
37.4	3.7	5.8	2.5	25.4	11.8
47.4	4.7	17.6	3.6	35.3	17.7
57.3	5.4	28.5	6.3	45.4	22.7
67.4	5.5	39.5	8.0		
77.4	7.1	49.5	9.2		
87.4	7.3	60.6	12.2		
97.4	8.7	71.5	14.3		
107.3	9.3	82.6	14.9		
117.4	10.4	93.5	18.0		
127.4	10.8	104.5	18.4		
137.4	11.5	115.5	21.2		
147.3	12.6	126.5	21.9		
157.3	13.3	137.5	23.8		
167.4	13.6				
177.3	14.3				
187.3	15.4				
197.4	16.1				
207.3	16.2				
217.3	17.1				
227.4	17.5				
237.4	17.6				
Rate $\propto [Ph_2SiH_2]^{1.18} \approx [Ph_2SiH_2]^1$					

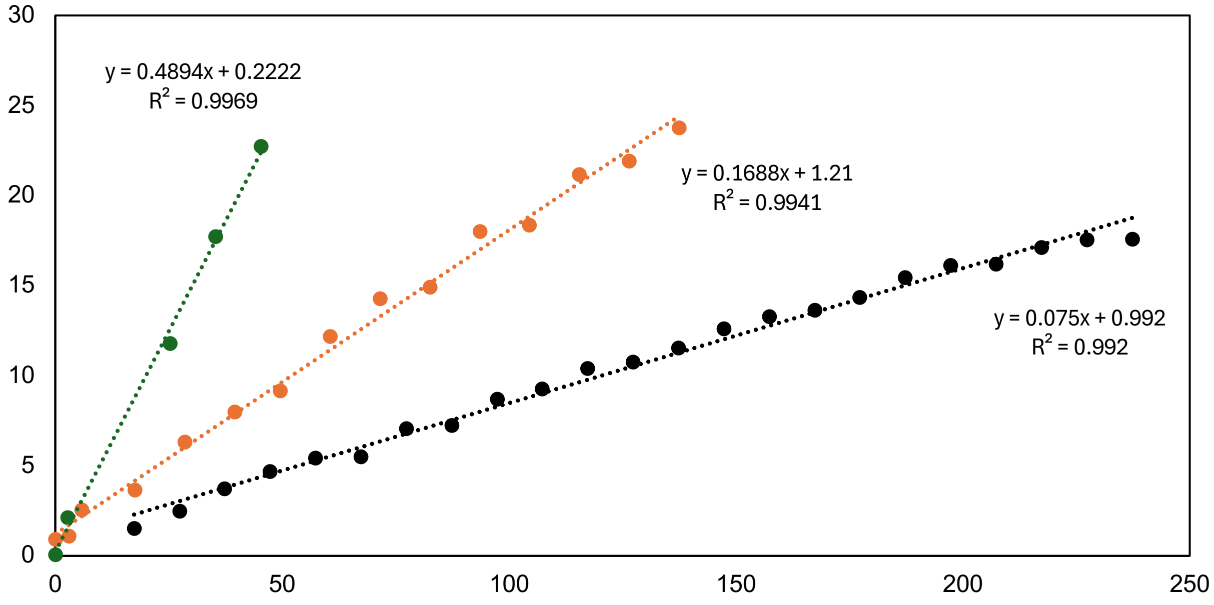

Figure S3. Reaction monitoring the reaction of **Mo1** with differing equivalents of Ph_2SiH_2. X-axis denotes time (mins) and y-axis denotes yield (%). Green denotes the reaction with 21.82 equiv Ph_2SiH_2, orange denotes the reaction with 13.04 equiv Ph_2SiH_2 and black denotes the reaction with 4.80 equiv Ph_2SiD_2.

IV. Photodriven Hydrogenation Reactions

General Procedure for the Photochemical Hydrogenation of Molybdenum Compounds. In a nitrogen-filled glovebox, a thick-walled glass vessel branched for the connection to vacuum line was charged with a magnetic stir bar and 0.01 mmol of the molybdenum compound. The contents were frozen in liquid nitrogen (–196 °C) followed by evacuation of air from the headspace *in vacuo*. 0.5 mL of THF was transferred under reduced pressure and the vessel was backfilled with H_2 gas. (*If* 4 atm of H_2 is needed: the glass vessel was submerged in liquid nitrogen and charged with 1 atm of H_2. The glass vessel was thawed under room temperature, bringing the internal pressure to approximately 4 atm.) The reaction mixture was slowly thawed and placed between two H150-Blue Kessil® lamps and cooled using air flow to maintain a consistent room temperature, confirmed with a thermometer during the reaction. The reaction was vigorously stirred for 72 h, and a color change from pink to colorless was observed.

General Procedure for Product Analysis. After completion of the reaction, the glass vessel was brought to a high vacuum line. The reaction mixture was cooled using liquid nitrogen and the headspace was removed under high vacuum. The volatiles of the crude mixture were transferred to another vessel charged with 3 mL of 2.0 M HCl solution in Et_2O which was frozen using liquid nitrogen. The frozen HCl solution was slowly thawed at room temperature and stirred for 5 min. All the volatiles were then removed in vacuo and the resulting solid was analyzed by ^{1}H NMR spectroscopy using 5.0 μL of 1,2-dichloroethane as an internal standard to confirm the generation of free ammonia. The non-volatile residue of the hydrogenation mixture was transferred into the N_2-filled glovebox. Triphenylphosphine was spiked into the crude mixture while the weight of added PPh_3 was measured by a difference of the batch of PPh_3 before and after the addition (weighing by subtraction), and it was dissolved in 0.4 mL THF-d_8 and transferred to a J. Young tube and analyzed by ^{1}H, ^{19}F and ^{31}P NMR spectroscopy. The solution was transferred to a 20 mL scintillation vial and the solvent was removed *in vacuo*. After trituration with pentane (3 x 5 mL), the pentane extract of the resulting crude mixture was filtered through a pad of celite using glass frit. The solvent was removed *in vacuo* and was dissolved in 0.4 mL THF-d_8 and transferred to a J. Young tube and analyzed by ^{1}H, ^{19}F and ^{31}P NMR, IR spectroscopy to confirm the absence of residual metal products, N–H bond or Si–H bonds. The solution was transferred to a thick-walled glass vessel, sealed, and removed from the glovebox. 3.0 mL of ether solution of HCl (2.0 M) and a few drops of distilled water was added and the mixture was heated at 70 ºC for 18 h. The mixture was slowly cooled down to the room temperature and the solvent was removed *in vacuo* and the resulting solid was analyzed by ^{1}H NMR spectroscopy using 5.0 μL of 1,2-dichloroethane as an internal standard to quantify the ammonium chloride generated from the N-containing products.

Initial Rate Studies with Different Concentration of H_2. In a nitrogen-filled glovebox, a 2.0 mL volumetric flask was charged with 18 mg (0.0115 mmol) of **Mo4**, 10 μL of mesitylene as an internal standard and THF was added to produce a total volume of solution as 2.0 mL. 0.45 mL of the stock solution was transferred into four separate J. Young NMR tubes. The NMR tubes were sealed and removed from the glovebox. The contents were frozen in liquid nitrogen (–196 ºC) followed by evacuation of air from the headspace *in vacuo*. Under reduced pressure, the vessel was filled with H_2 gas. (*If* 4 atm of H_2 is needed: the glass vessel was submerged in liquid nitrogen and charged with 1 atm of H_2; For 2 atm: the glass vessel was submerged in liquid nitrogen and charged with 0.5 atm of H_2; For 1 atm: the solution was frozen in liquid nitrogen and charged with 1 atm of H_2; For 1 atm: the solution was frozen in liquid nitrogen and charged with 0.5 atm of H_2.) The reaction mixture was slowly thawed and placed between two H150-Blue Kessil® lamps and cooled using air flow to maintain a consistent room temperature, confirmed with a thermometer during the reaction. The reaction was monitored using ^{1}H NMR spectroscopy and the generated **Mo2** was quantified using mesitylene as an internal standard (Table S5). The effective concentration of H_2 was also measured using the same method.

Table S5. Monitoring of the generation of **Mo2** during the photochemical hydrogenation of **Mo4** under different pressure of H_2.

Time (h)	Yield (%), 0.5 atm	Yield (%), 1 atm	Yield (%), 2 atm	Yield (%), 4 atm
0	0	0	0	0
7.5	4.3	5.1	5.3	4.5
9.83	6.9	7.1	7.5	5.8
14.5	9.2	9.8	11.0	11.0
17.5	11.4	10.6	12.6	13.1
20.5	12.9	12.0	14.5	14.6
27	16.2	16.5	17.0	18.1
30.5	19.0	18.0	19.0	20.1

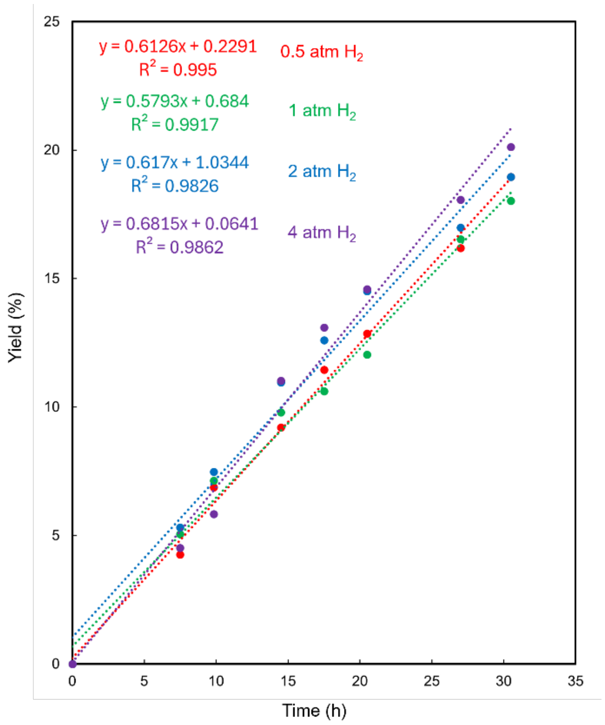

Figure S4. Reaction monitoring of the photochemical hydrogenation of **Mo4** under different pressures of H_2 (0.5, 1, 2, 4 atm).

Parallel KIE experiments for the photochemical hydrogenation with Mo4 and Mo4-D2. In a nitrogen-filled glovebox, two J. Young NMR tubes were separated charged with 5 mg (0.0032 mmol) of **Mo4** and **Mo4-D2**, respectively, a glass capillary charged with a benzene-d_6 solution of PPh_3 as an external standard and 0.45 mL of THF. The NMR tubes were sealed and removed from the glovebox. The contents were frozen in liquid nitrogen (-196 ºC) followed by evacuation of air from the headspace *in vacuo*. Under reduced pressure, the vessel was filled with H_2 gas. (*If* 4 atm of H_2 is needed: the glass vessel was submerged in liquid nitrogen and charged with 1 atm of H_2.) The reaction mixture was slowly thawed and placed between two H150-Blue Kessil® lamps and cooled using air flow to maintain a consistent room temperature, confirmed with a thermometer during the reaction. The generation of **Mo2** was monitored using ^{31}P NMR spectroscopy and quantified using PPh_3 as an external standard (Table S6).

Table S6. Monitoring of the generation of **Mo2** during the photochemical hydrogenation of **Mo4** and **Mo4-D2** in a separated NMR tube.

Run 1			Run 2			Run 3		
Time (h)	Yield (%), H	Yield (%), D	Time (h)	Yield (%), H	Yield (%), D	Time (h)	Yield (%), H	Yield (%), D
0	0	0	0	0	0	0	0	0
1	3.3	0.9	1	5.2	0.7	1	3.2	0.3
2	8.1	1.4	2	9.1	1.3	2	7.4	0.5
3	11.3	1.5	3	13.1	1.8	3	11.7	1.0
4	15.2	1.9	4	17.4	2.4	4	15.3	1.4
5	19.8	2.2	5		2.6	5		1.7
6		2.9	10		4.6	10		3.1
10.5		4.8	17		8.2	17		4.9
15		6.0						
20		8.7						
34		12.6						
43	78.7	16.6						
KIE = 10.5			KIE = 9.5			KIE = 12.1		

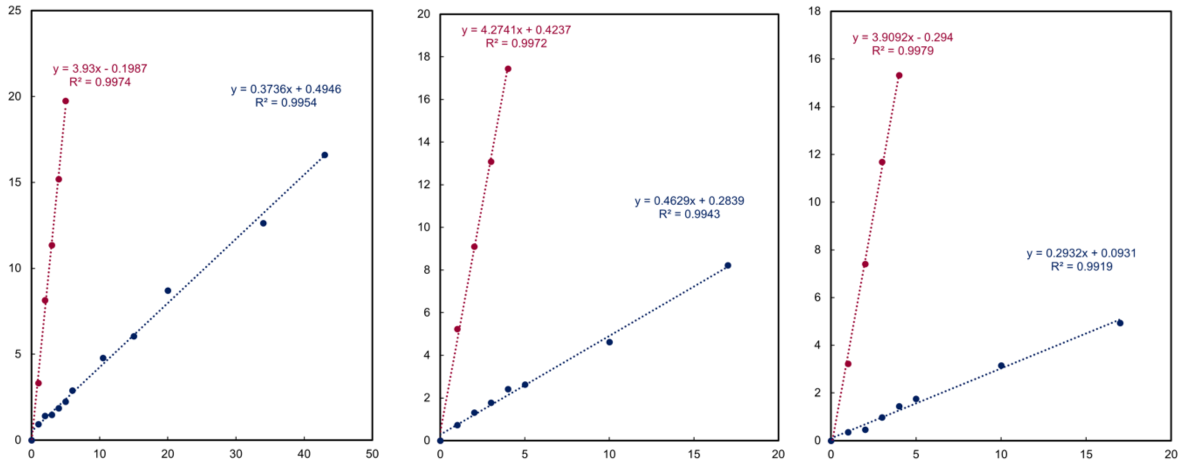

Figure S5. Reaction monitoring of the parallel KIE experiment for the photochemical hydrogenation with **Mo4** and **Mo4-D$_2$**. Left: 1st run, middle: 2nd run and right: 3rd run. X-axis denotes time (mins) and y-axis denotes yield (%). Maroon denotes the reaction with **Mo4** and dark blue denotes the reaction with **Mo4-D$_2$**.

V. Spectroscopic Data

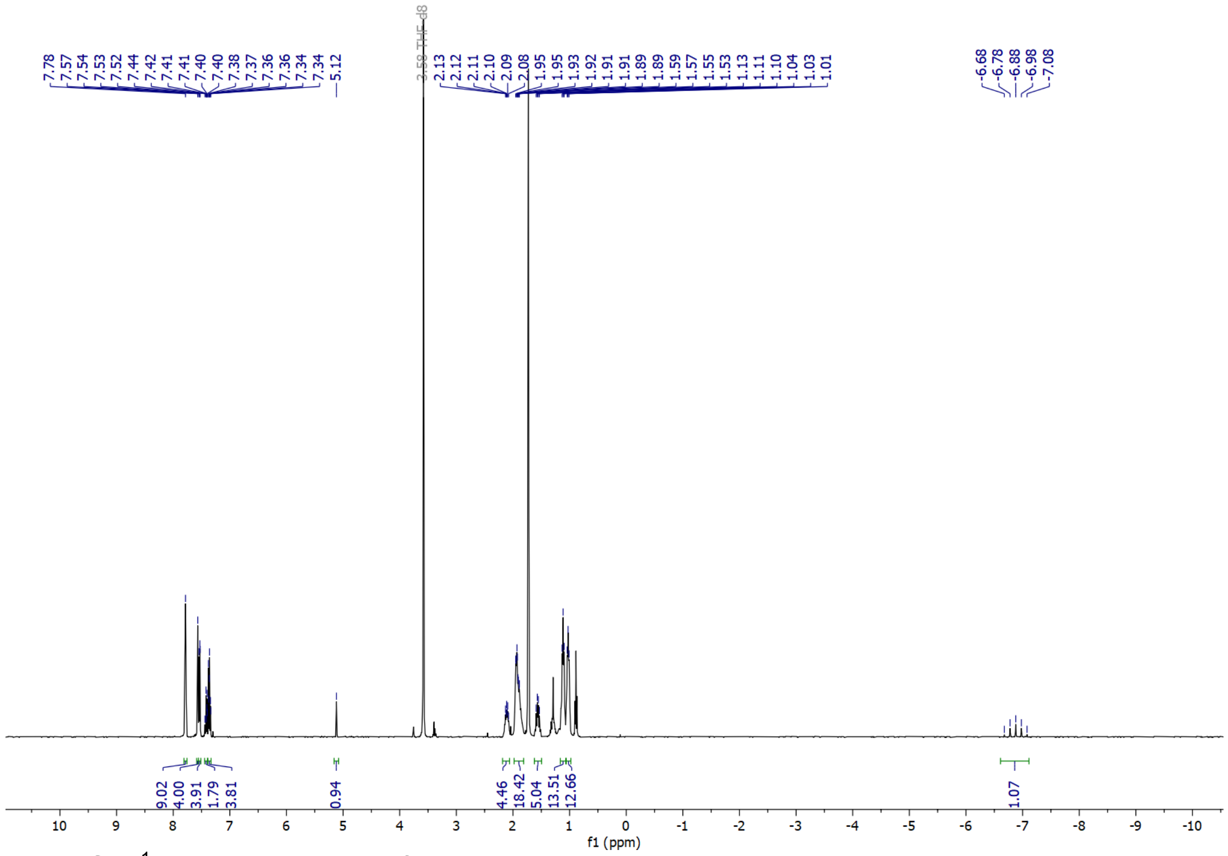

Figure S6. ^{1}H NMR spectrum of **Mo4** in THF-d_8.

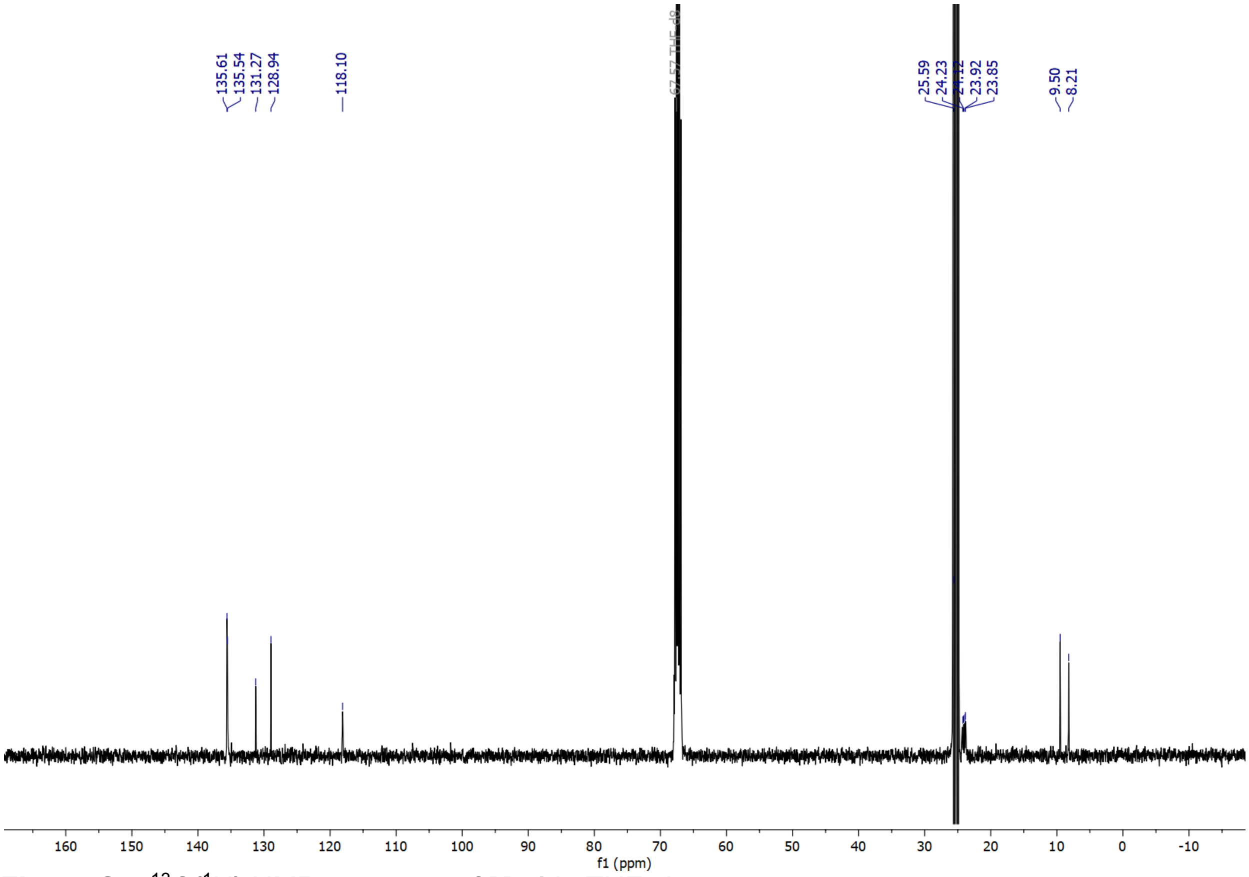

Figure S7. $^{13}C\{^{1}H\}$ NMR spectrum of **Mo4** in THF-d_8.

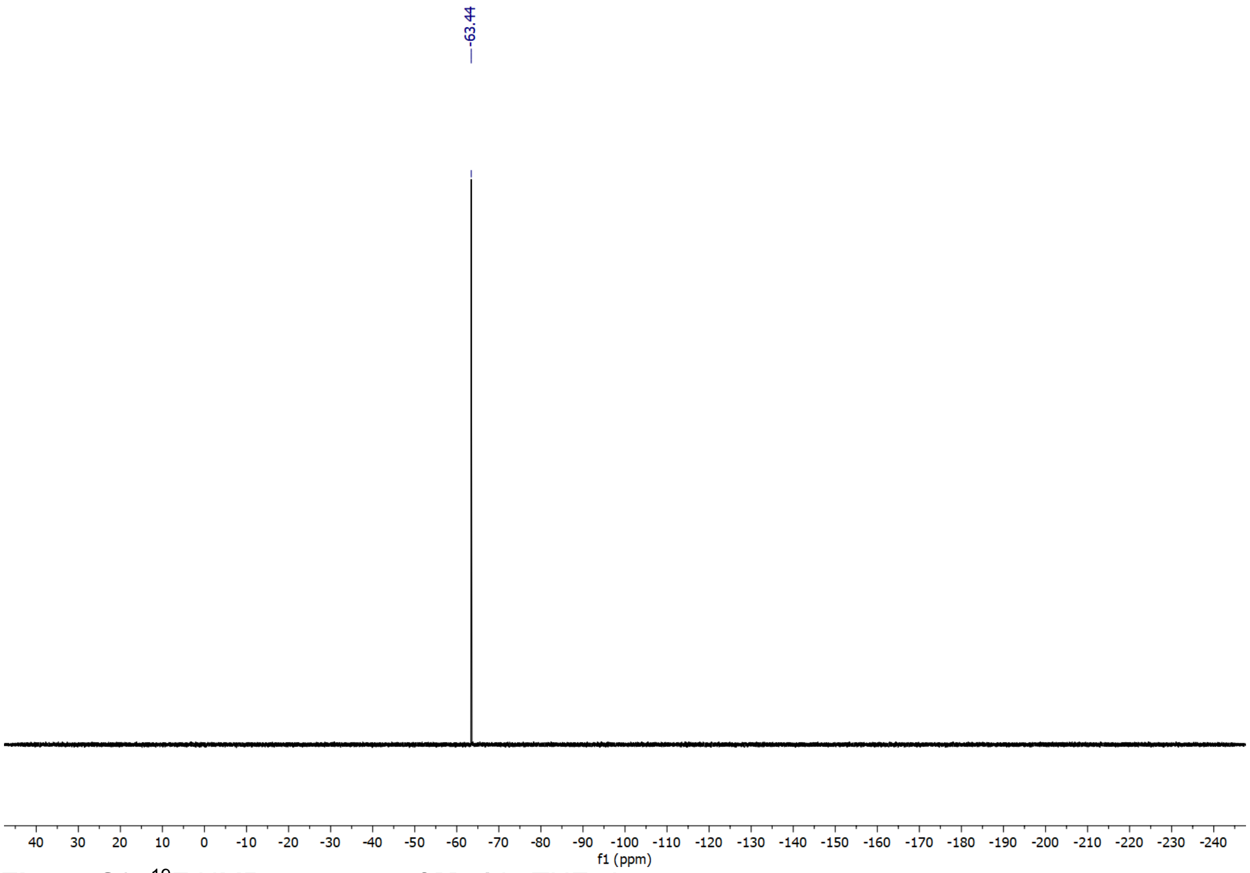

Figure S8. ^{19}F NMR spectrum of **Mo4** in THF-d_8.

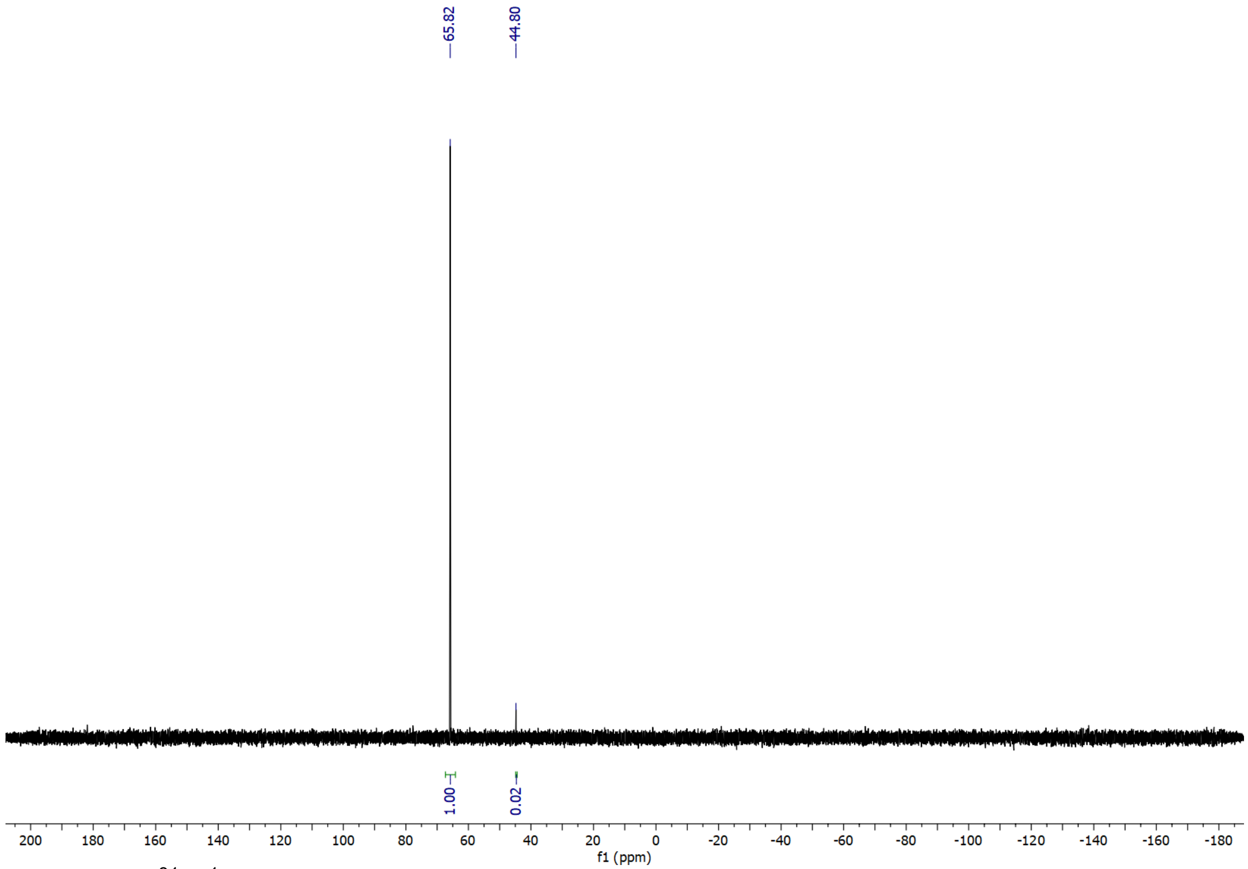

Figure S9. $^{31}P\{^1H\}$ NMR spectrum of **Mo4** in THF-d_8.

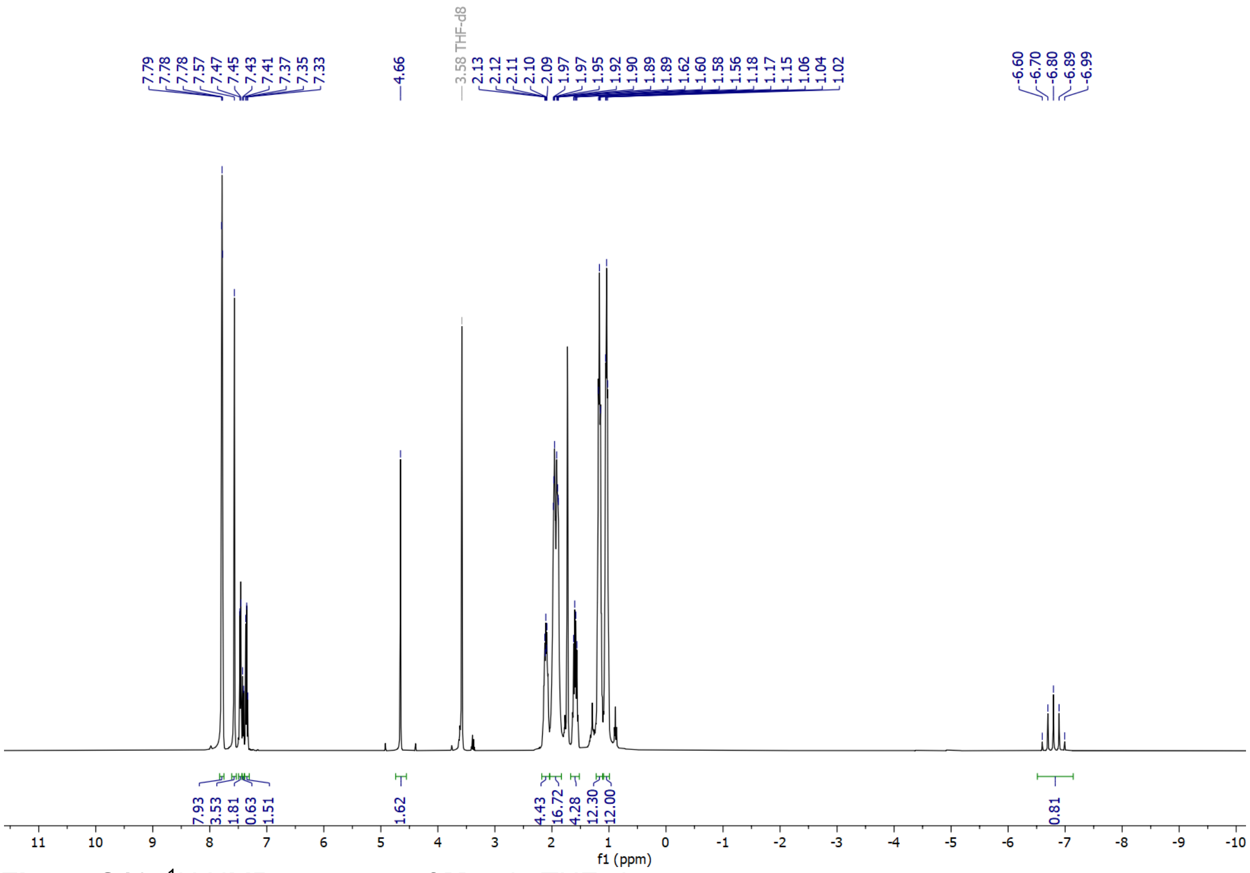

Figure S10. ^{1}H NMR spectrum of **Mo5** in THF-d_8

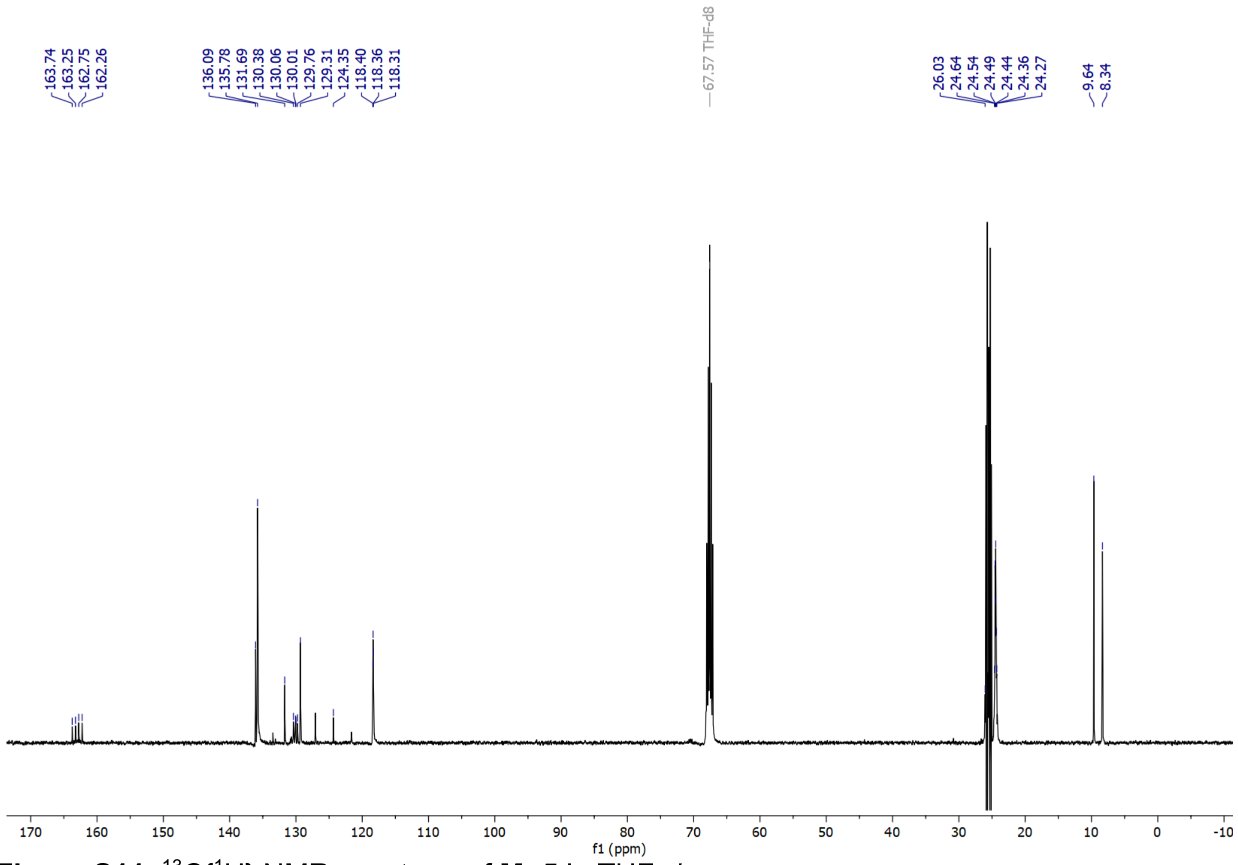

Figure S11. $^{13}C\{^{1}H\}$ NMR spectrum of **Mo5** in THF-d_8.

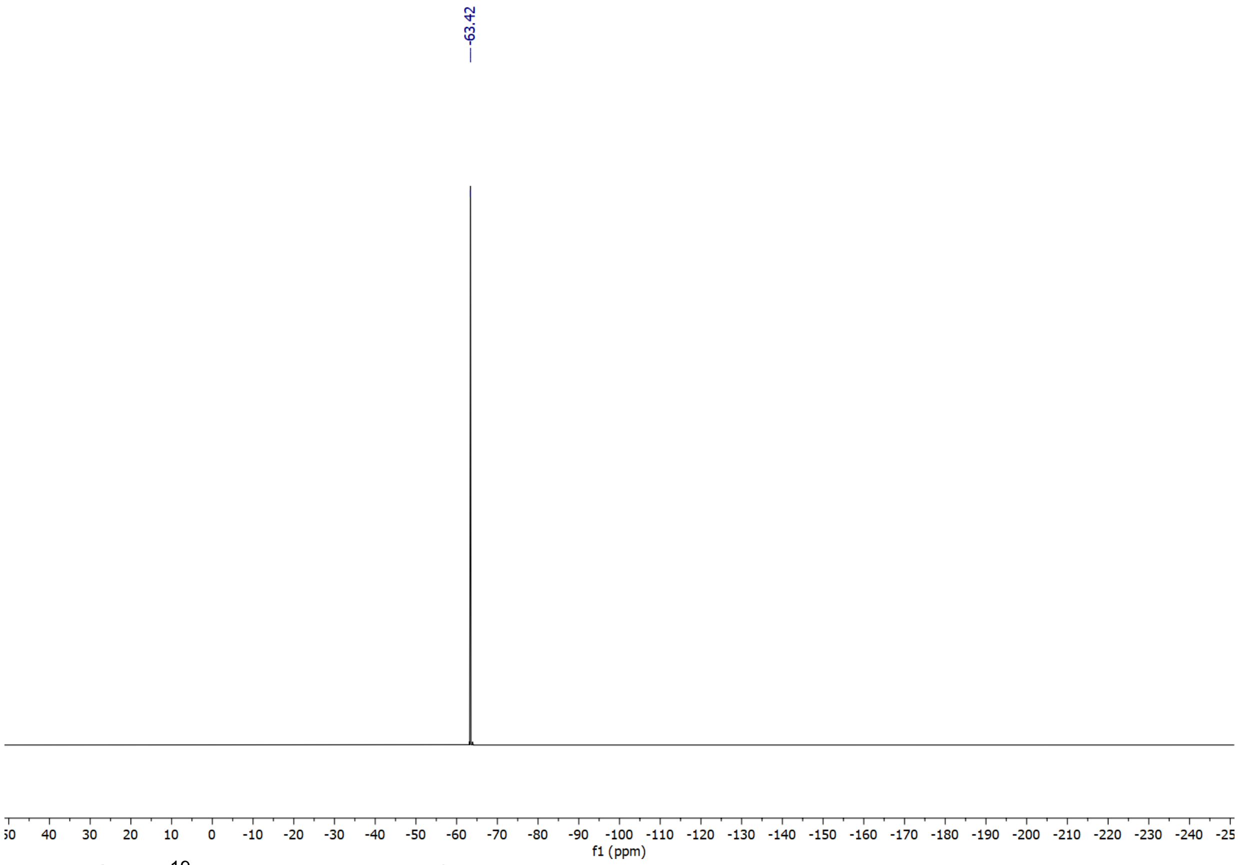

Figure S12. ^{19}F NMR spectrum of **Mo5** in THF-d_8.

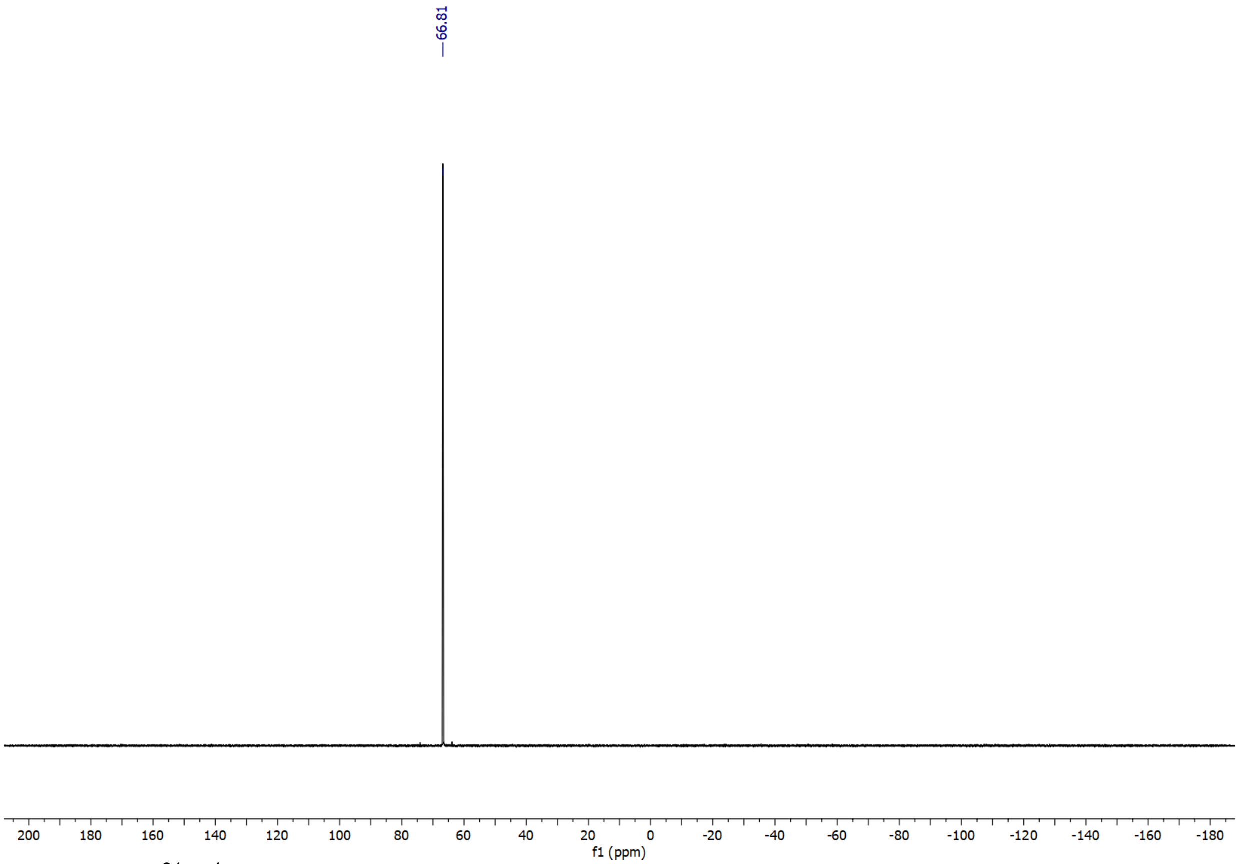

Figure S13. $^{31}P\{^{1}H\}$ NMR spectrum of **Mo5** in THF-d_8.

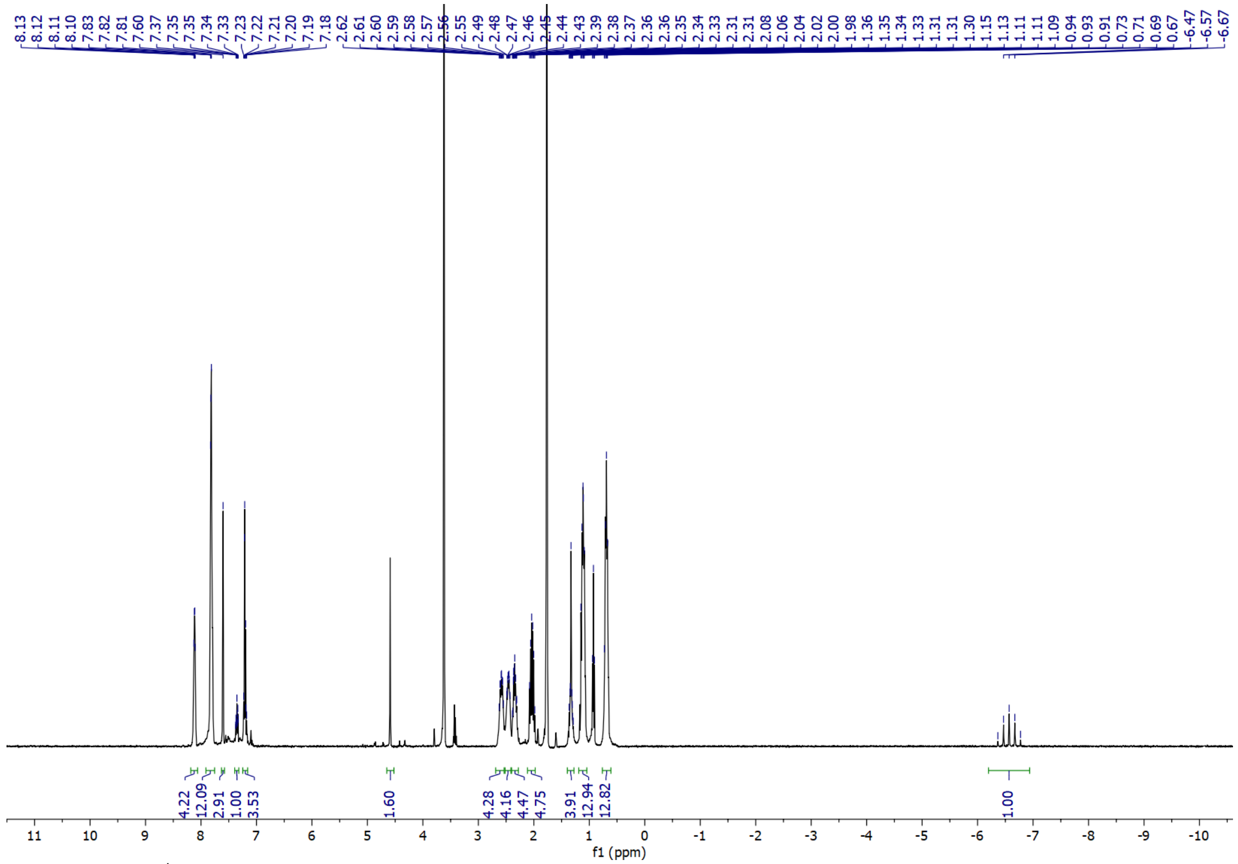

Figure S14. ^{1}H NMR spectrum of **Mo9** in THF-d_8.

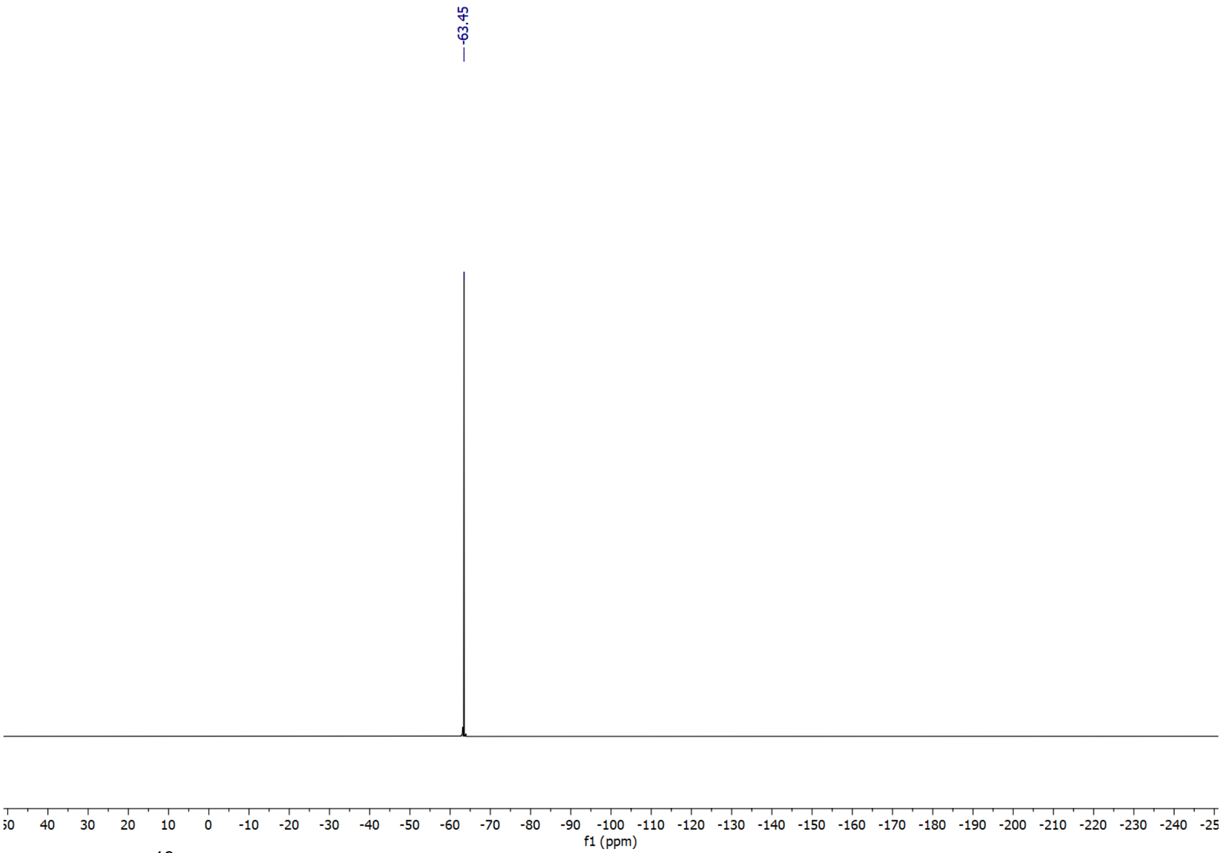

Figure S15. ^{19}F NMR spectrum of **Mo9** in THF-d_8.

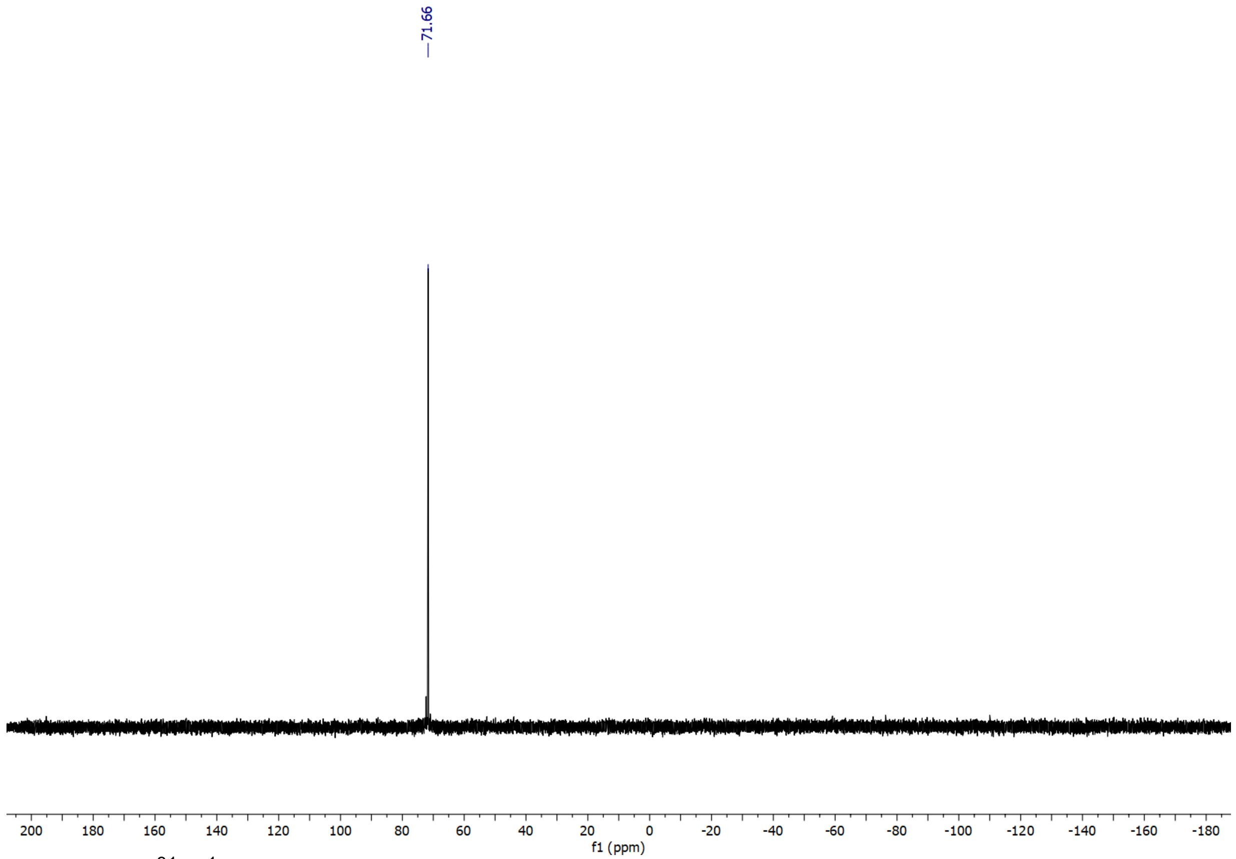

Figure S16. $^{31}P\{^1H\}$ NMR spectrum of **Mo9** in THF-d_8.

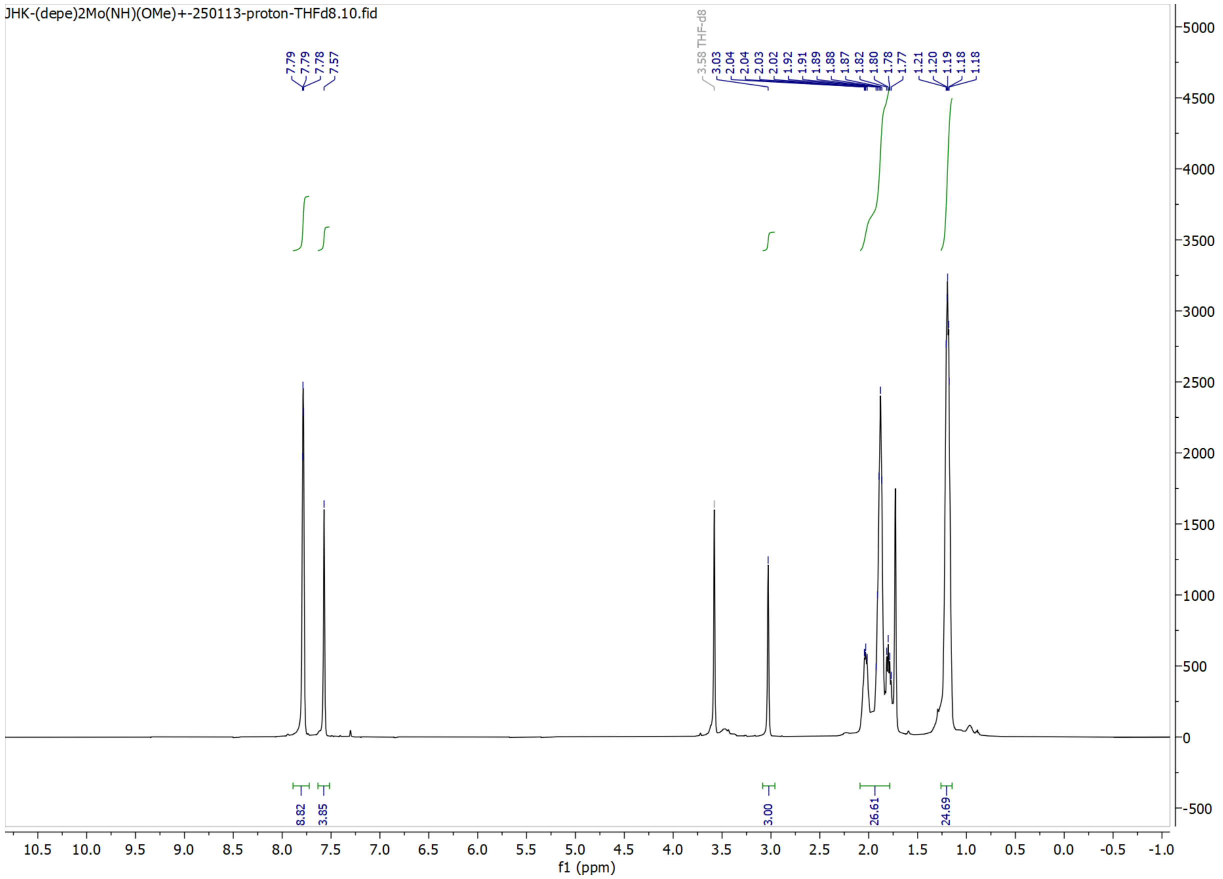

Figure S17. ^{1}H NMR spectrum of **Mo14** in THF-d_8.

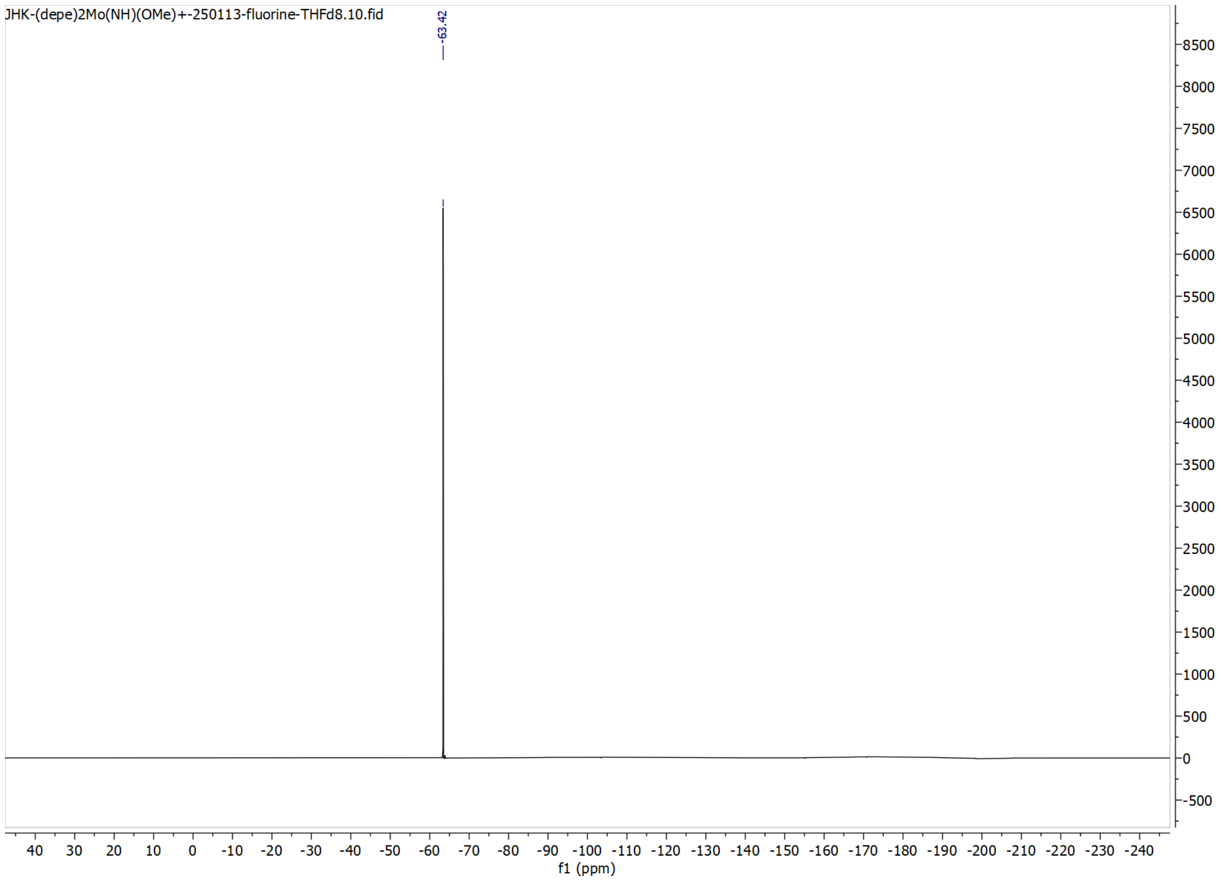

Figure S18. ^{19}F NMR spectrum of **Mo14** in THF-d_8.

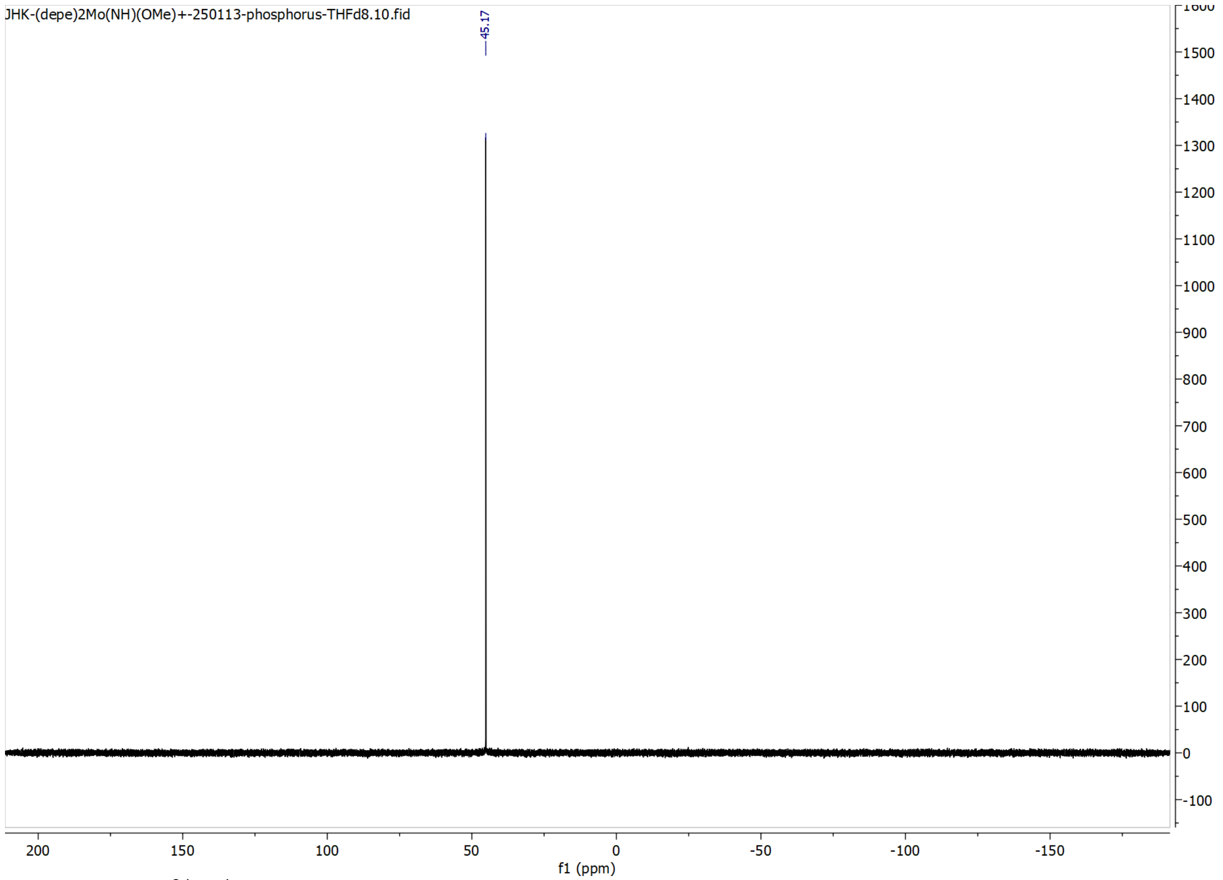

Figure S19. $^{31}P\{^{1}H\}$ NMR spectrum of **Mo14** in THF-d_8.

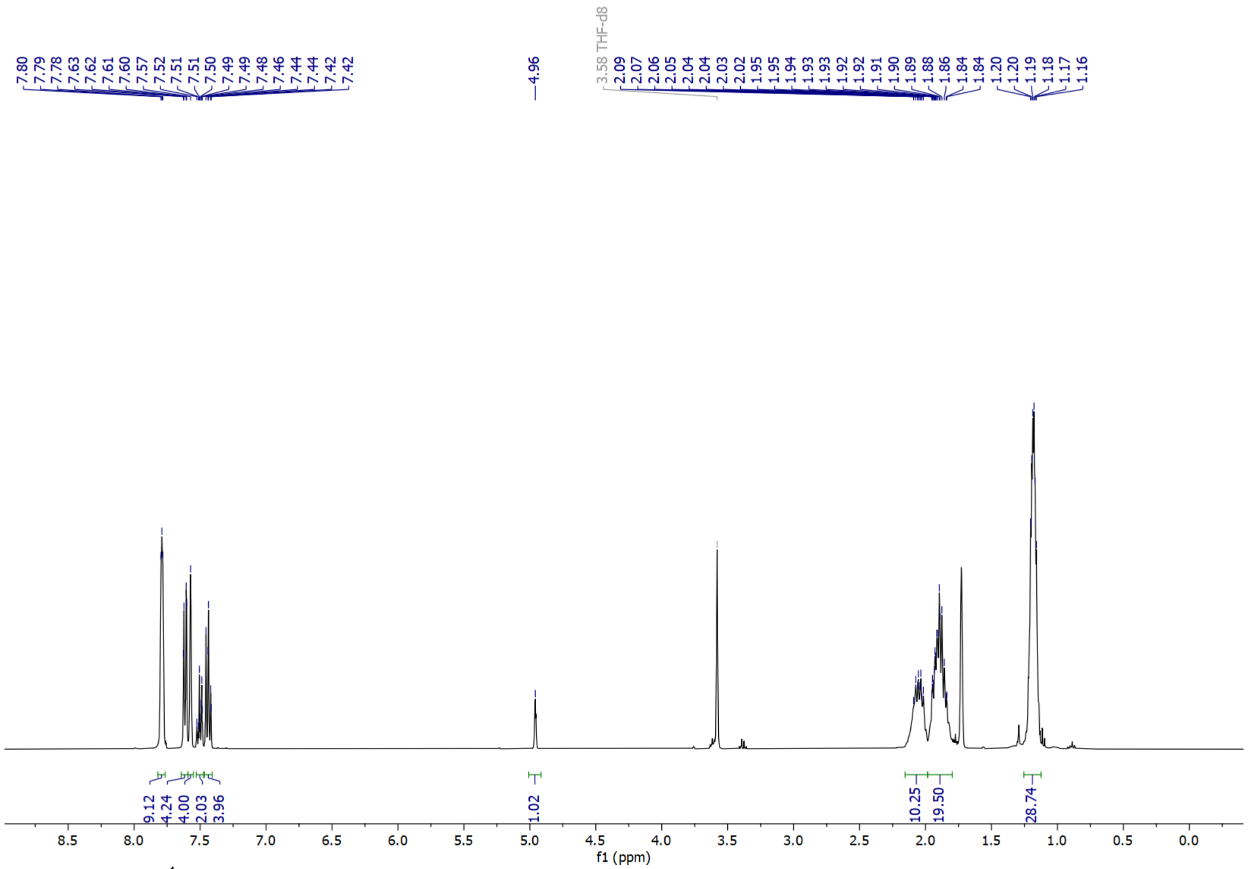

Figure S20. ^{1}H NMR spectrum of **Mo15** in THF-d_8.

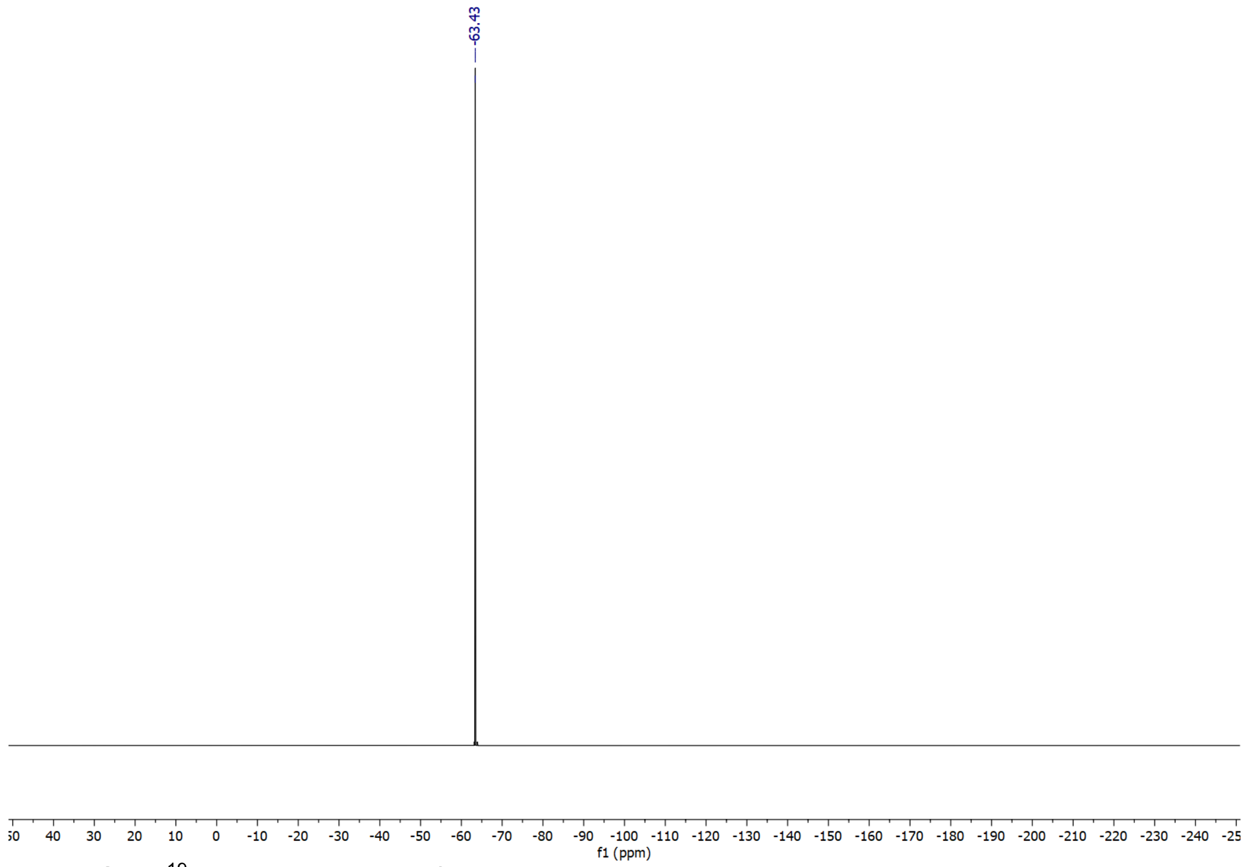

Figure S21. ^{19}F NMR spectrum of **Mo15** in THF-d_8

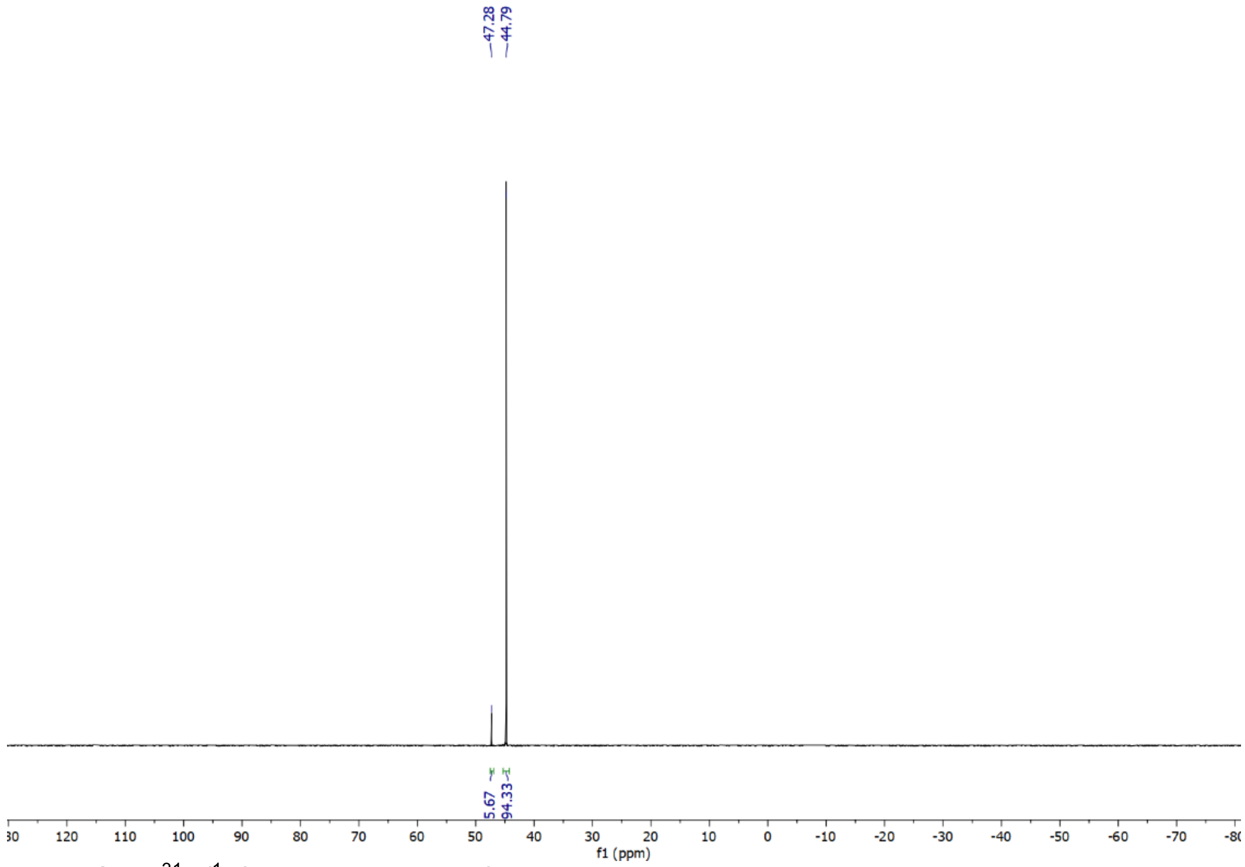

Figure S22. $^{31}P\{^1H\}$ NMR spectrum of **Mo15** in THF-d_8. A singlet at 47.3 ppm is trace generated **Mo13** as an impurity .

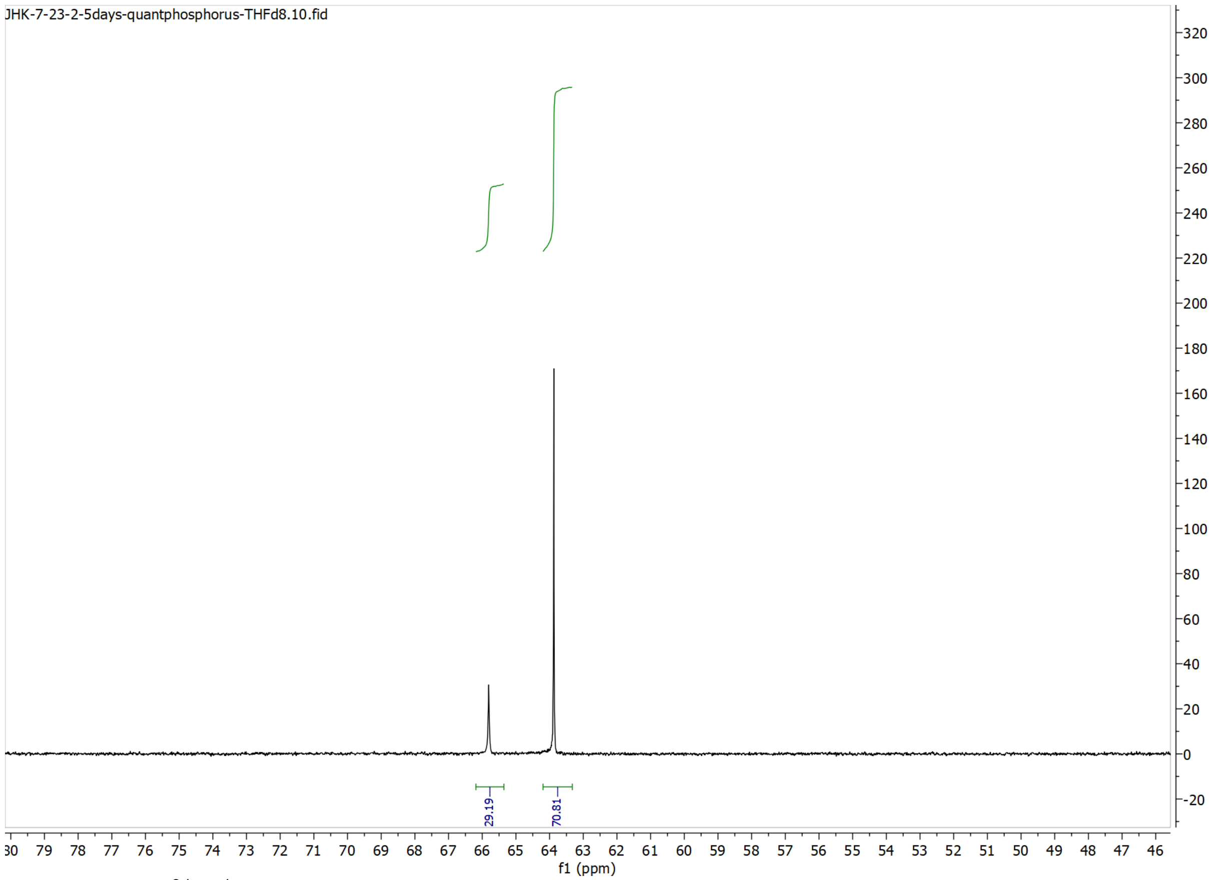

Figure S23. $^{31}P\{^{1}H\}$ NMR spectrum of **Mo4** heated to 100 °C for 5 days in THF-d_8, generating 3:7 mixture of **Mo4** and **Mo1**, respectively.

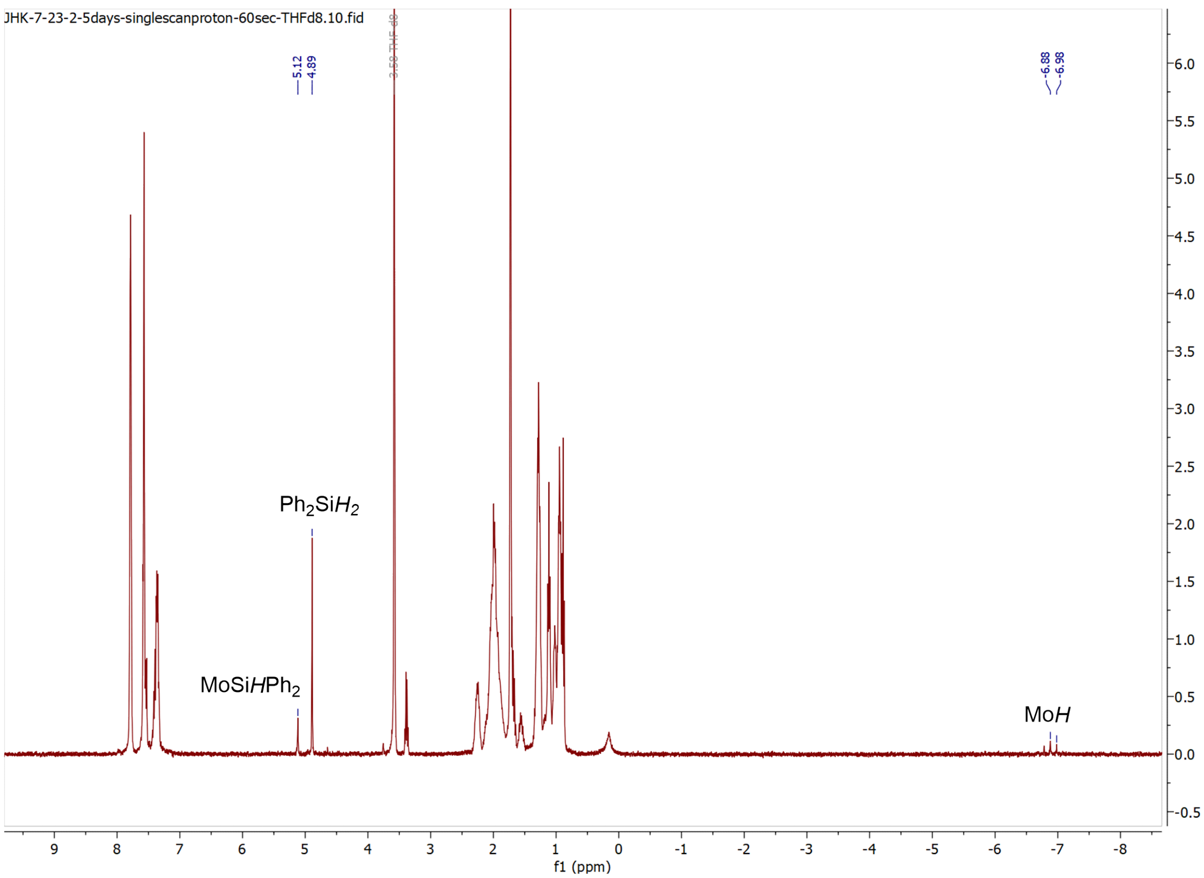

Figure S24. ^{1}H NMR spectrum of **Mo4** heated to 100 °C for 5 days in THF-d_8 supporting the generation of Ph_2SiH_2 and **Mo1**.

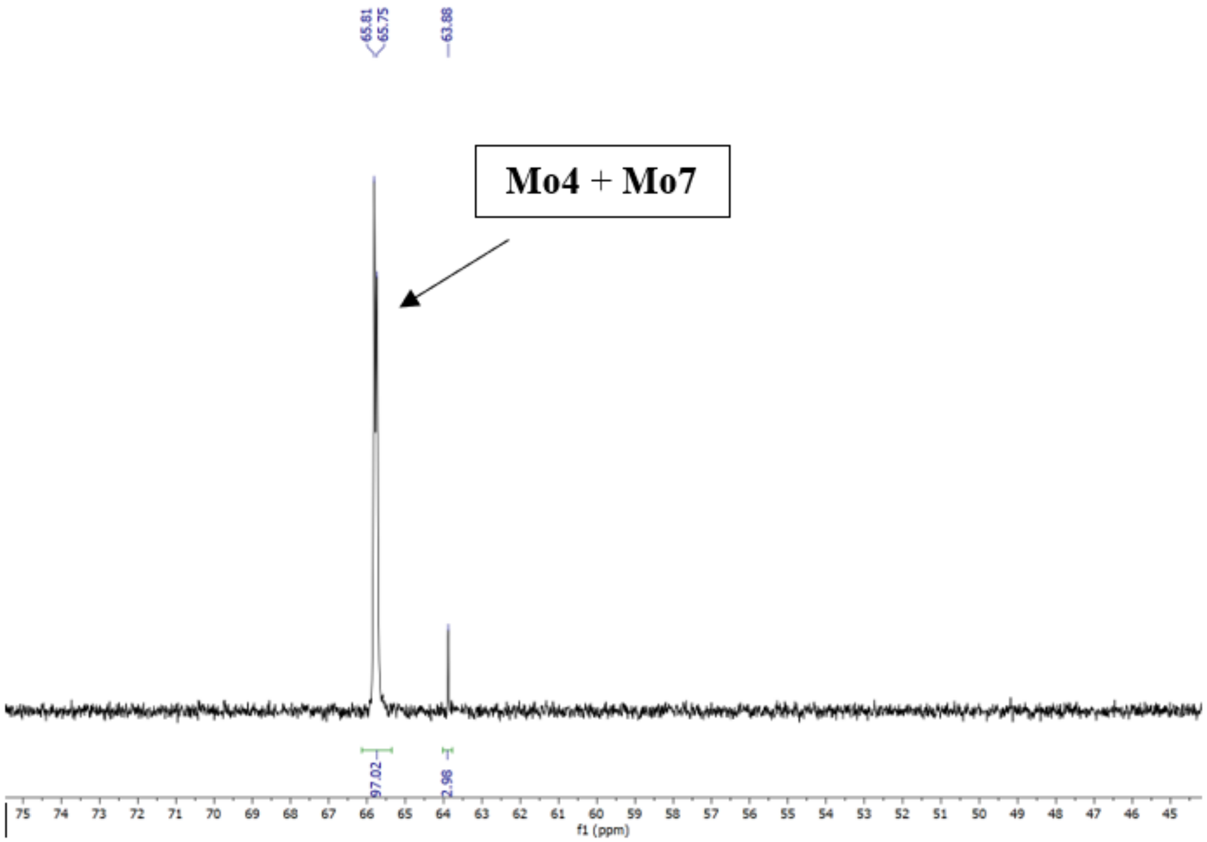

Figure S25. $^{31}P\{^{1}H\}$ NMR spectrum of the competition experiment with **Mo1**, Ph_2SiH_2, (*i*-tol)$_2SiH_2$ after 30 h in THF-d_8.

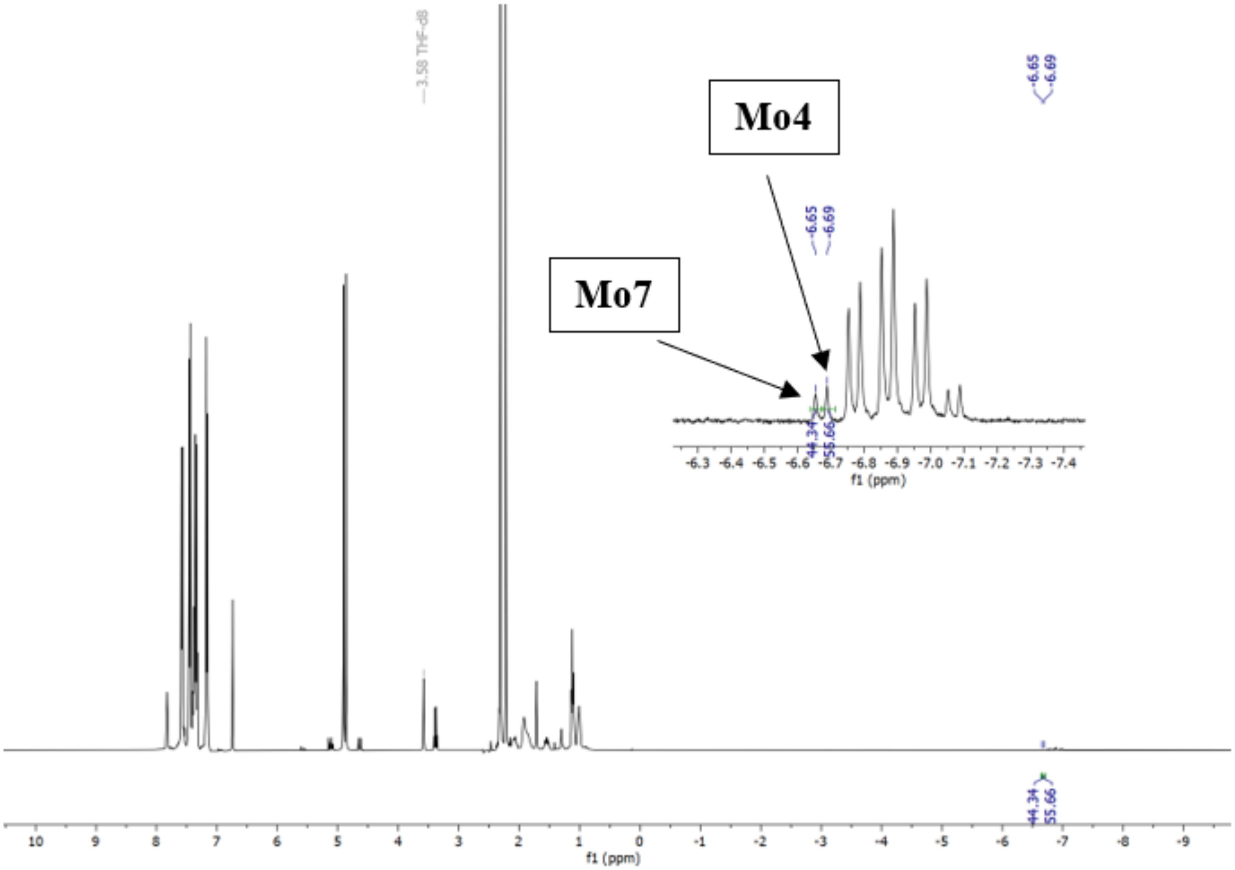

Figure S26. ^{1}H NMR spectrum of the competition experiment with **Mo1**, Ph_2SiH_2, (p-tol)$_2SiH_2$ after 30 h in THF-d_8.

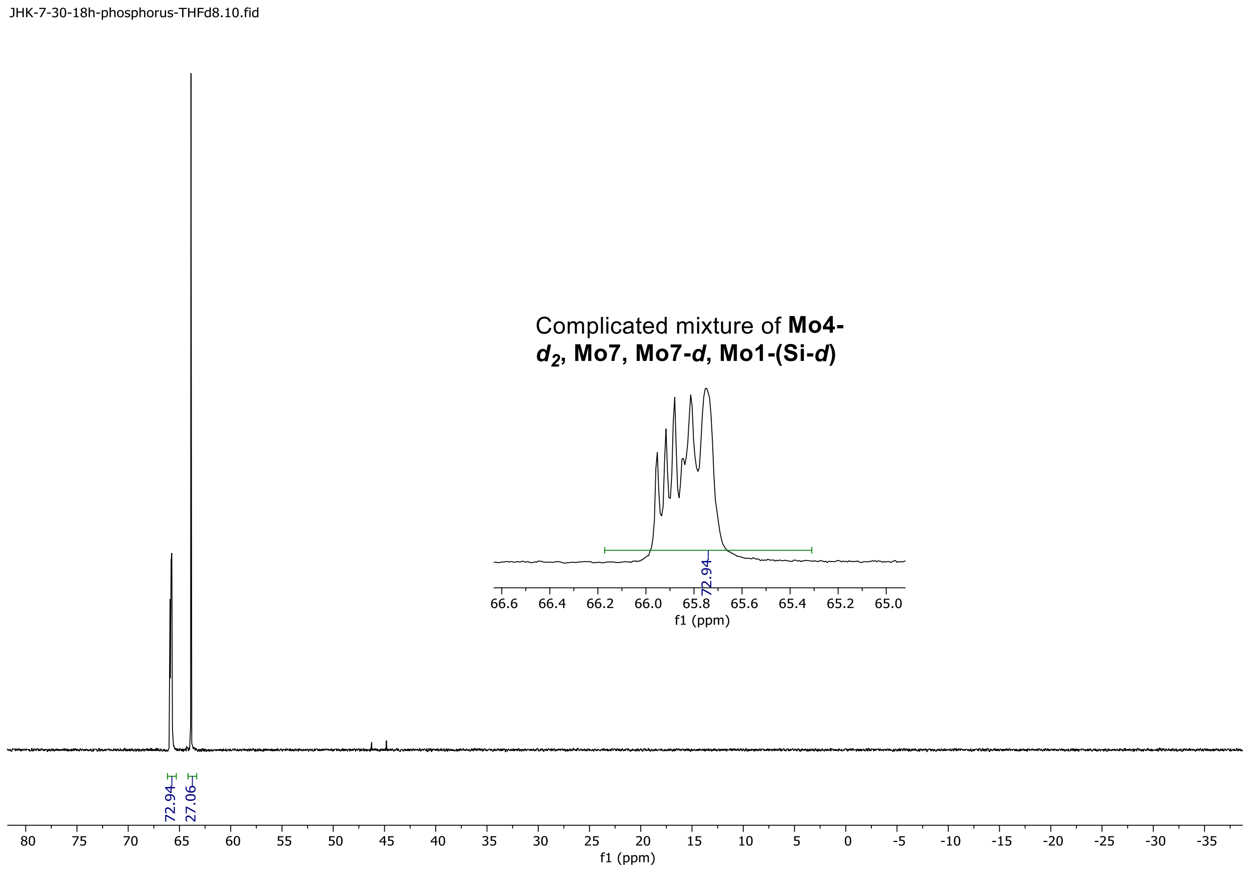

Figure S27. $^{31}P\{^1H\}$ NMR spectrum of the crossover experiment (18 h) with **Mo1** and Ph_2SiH_2/(*p*-tol)$_2SiD_2$ in THF-d_8.

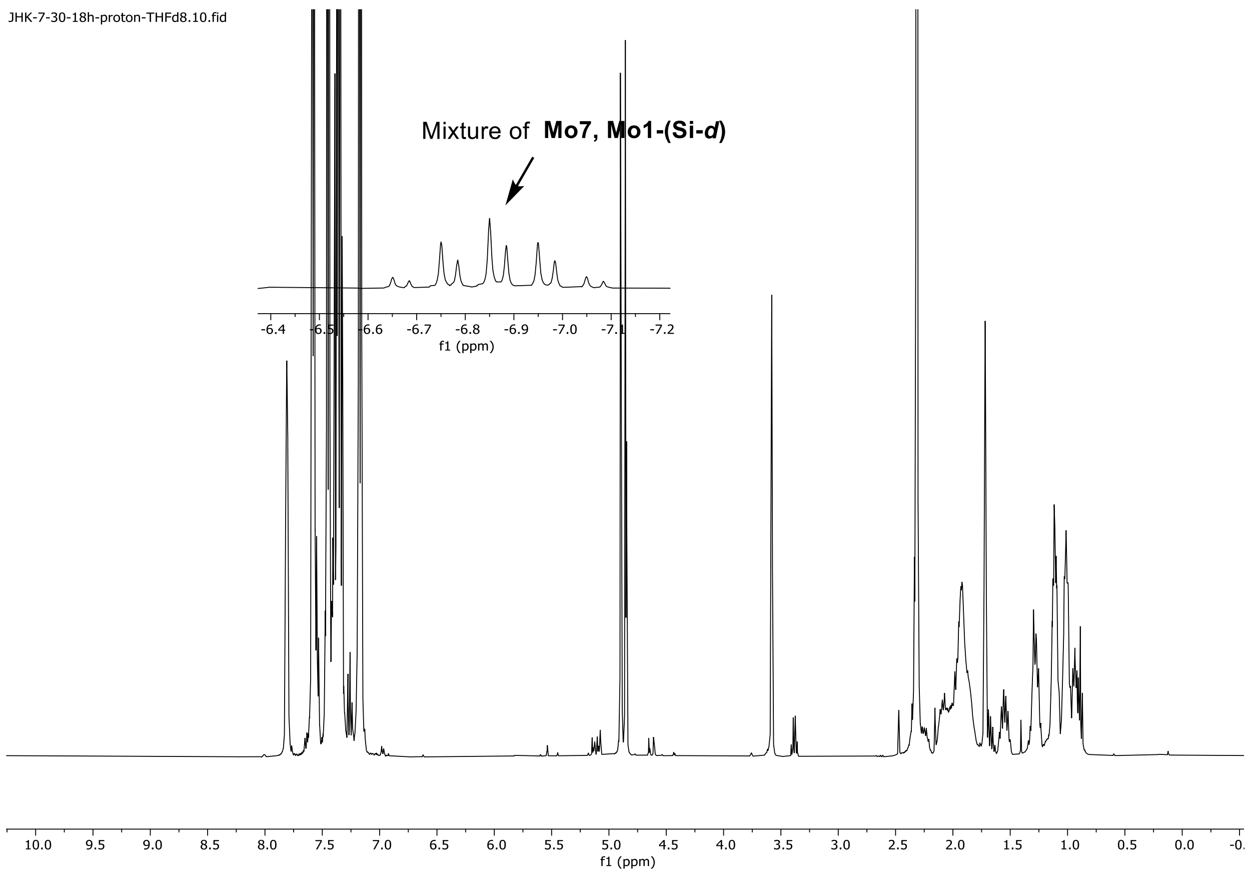

Figure S28. ^{1}H NMR spectrum of the crossover experiment (18 h) with **Mo1** and Ph_2SiH_2/(*p*-tol)$_2SiD_2$ in THF-d_8 highlighting the Mo-H region.

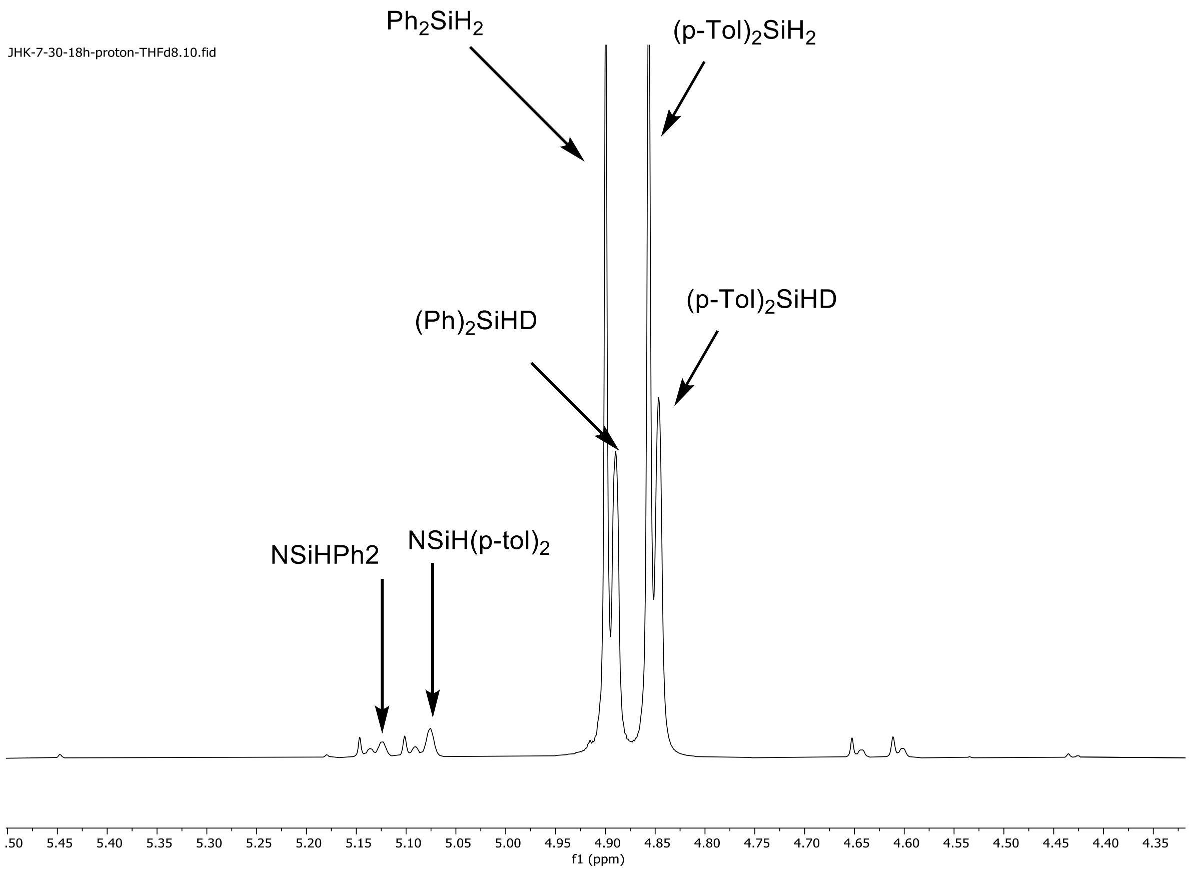

Figure S29. ^{1}H NMR spectrum of the crossover experiment (18 h) with **Mo1** and Ph_2SiH_2/(*p*-tol)$_2SiD_2$ in THF-d_8 highlighting the silane region.

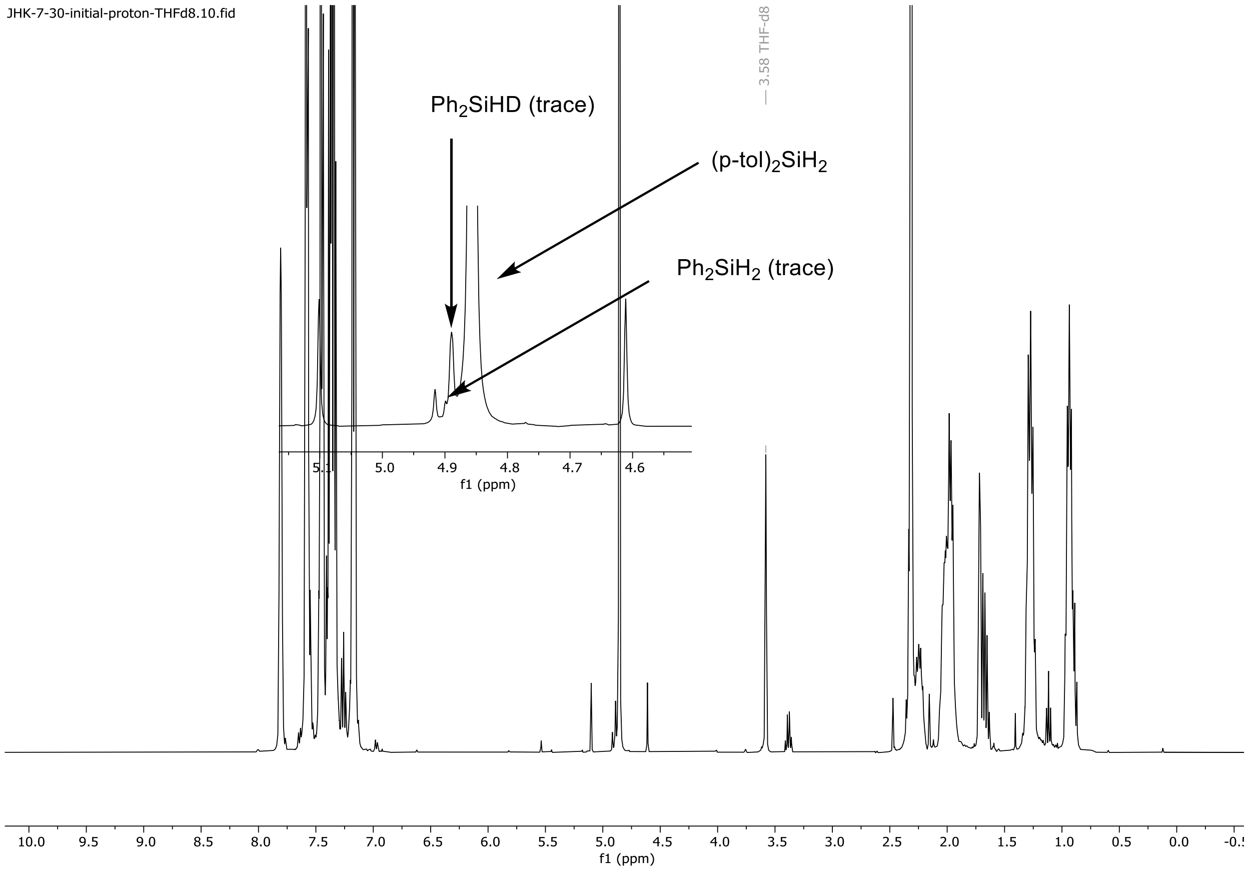

Figure S30. ^{1}H NMR spectrum of the crossover experiment (0 h) with **Mo1** and Ph_2SiH_2/(*p*-tol)$_2SiD_2$ in THF-d_8 highlighting the silane region, showing the trace presence of Ph_2SiHD.

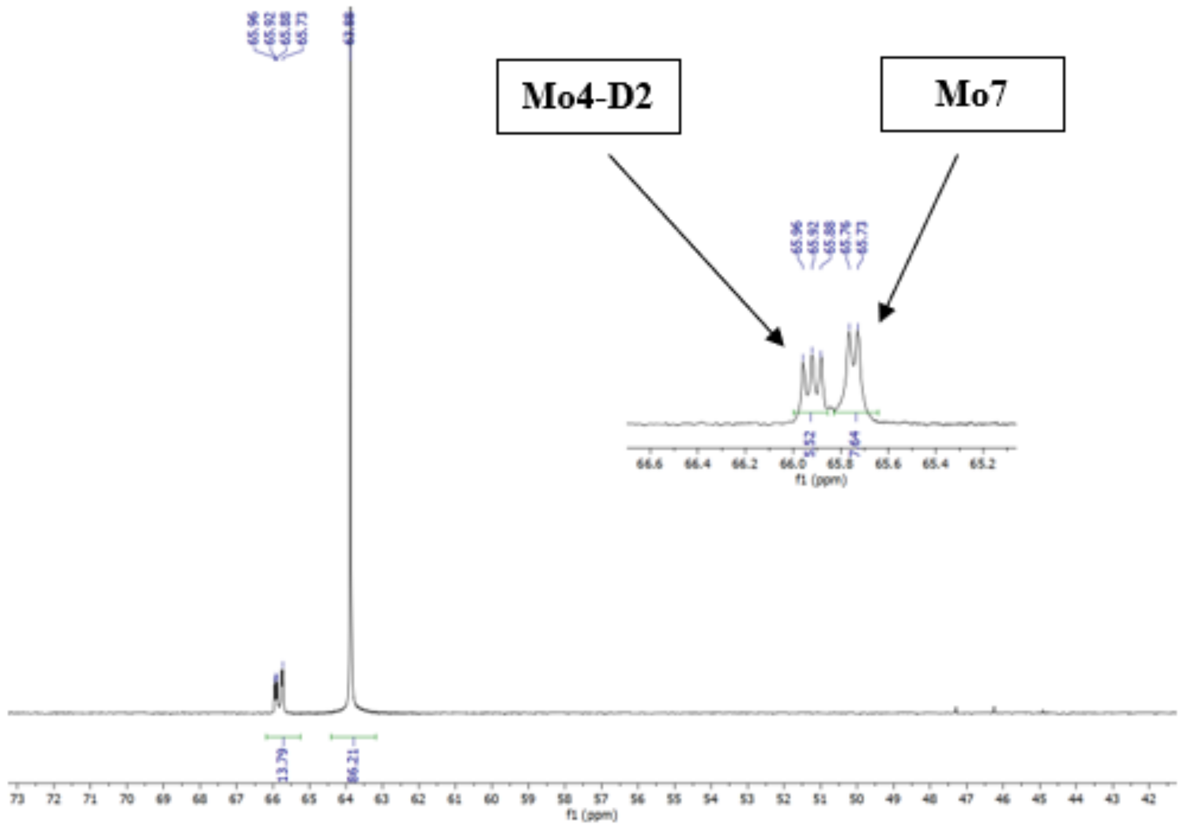

Figure S31. $^{31}P\{^1H\}$ NMR spectrum of the crossover experiment (75 mins) with **Mo1** and $Ph_2SiH_2/(p\text{-tol})_2SiD_2$ in THF-d_8.

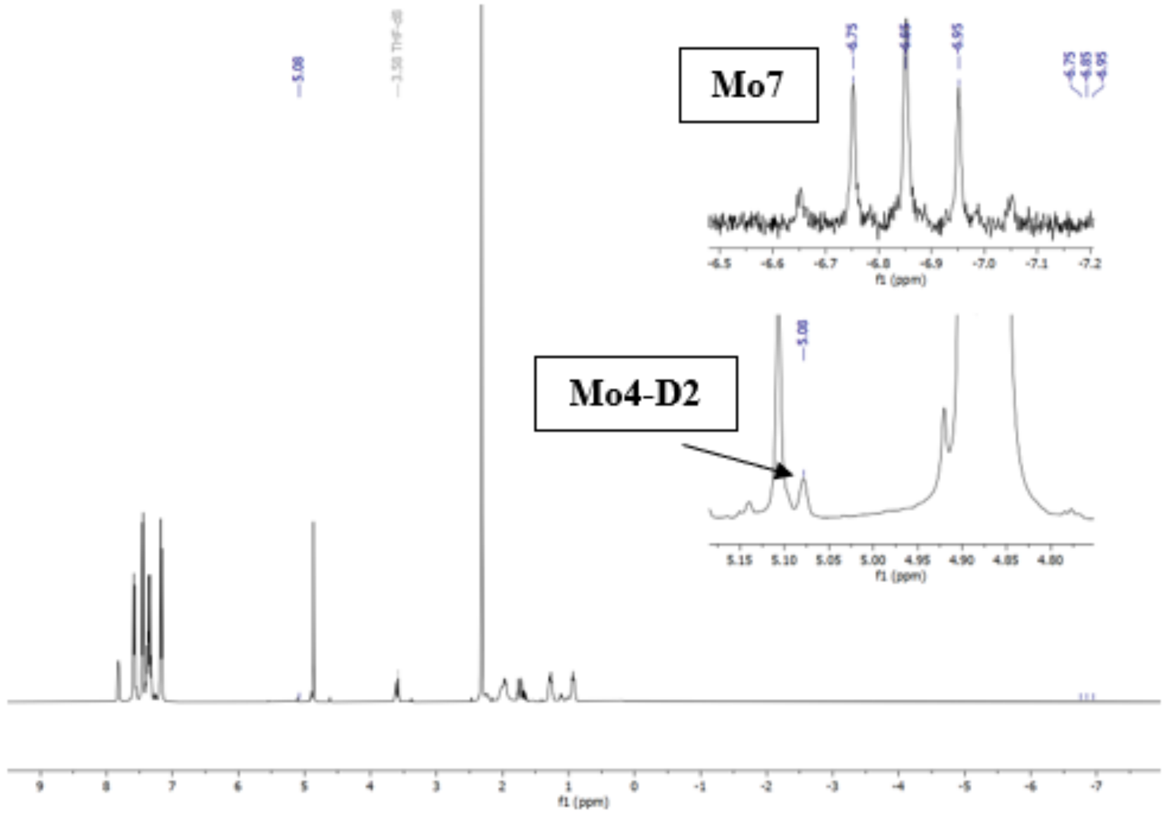

Figure S32. ^{1}H NMR spectrum of the crossover experiment (75 mins) with **Mo1** and Ph_2SiH_2/(*p*-tol)$_2SiD_2$ in THF-d_8.

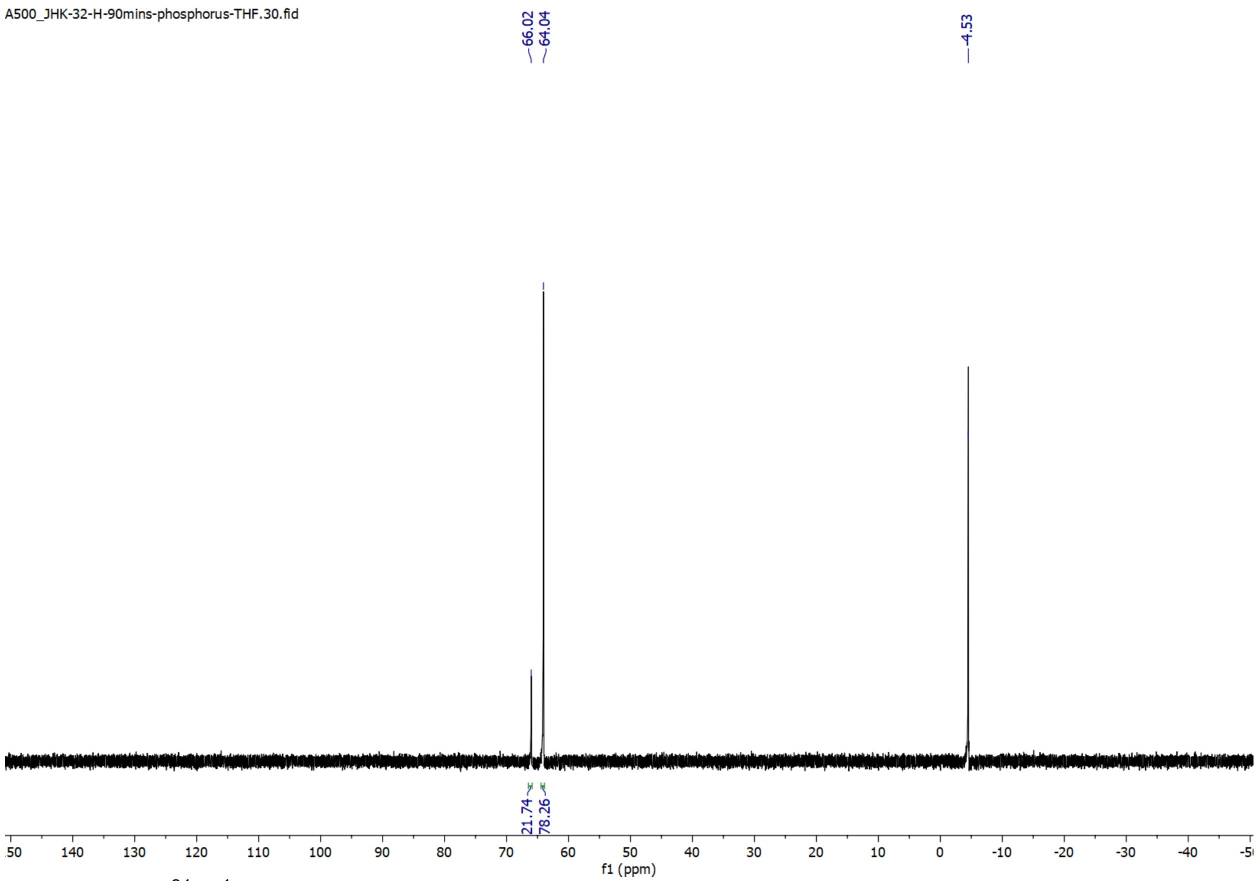

Figure S33. $^{31}P\{^{1}H\}$ NMR spectrum of a mixture of **Mo1** and 15 equiv Ph_2SiH_2 at 60 °C for 90 min (KIE experiment, run 1) in THF with a capillary filled with benzene-d_6.

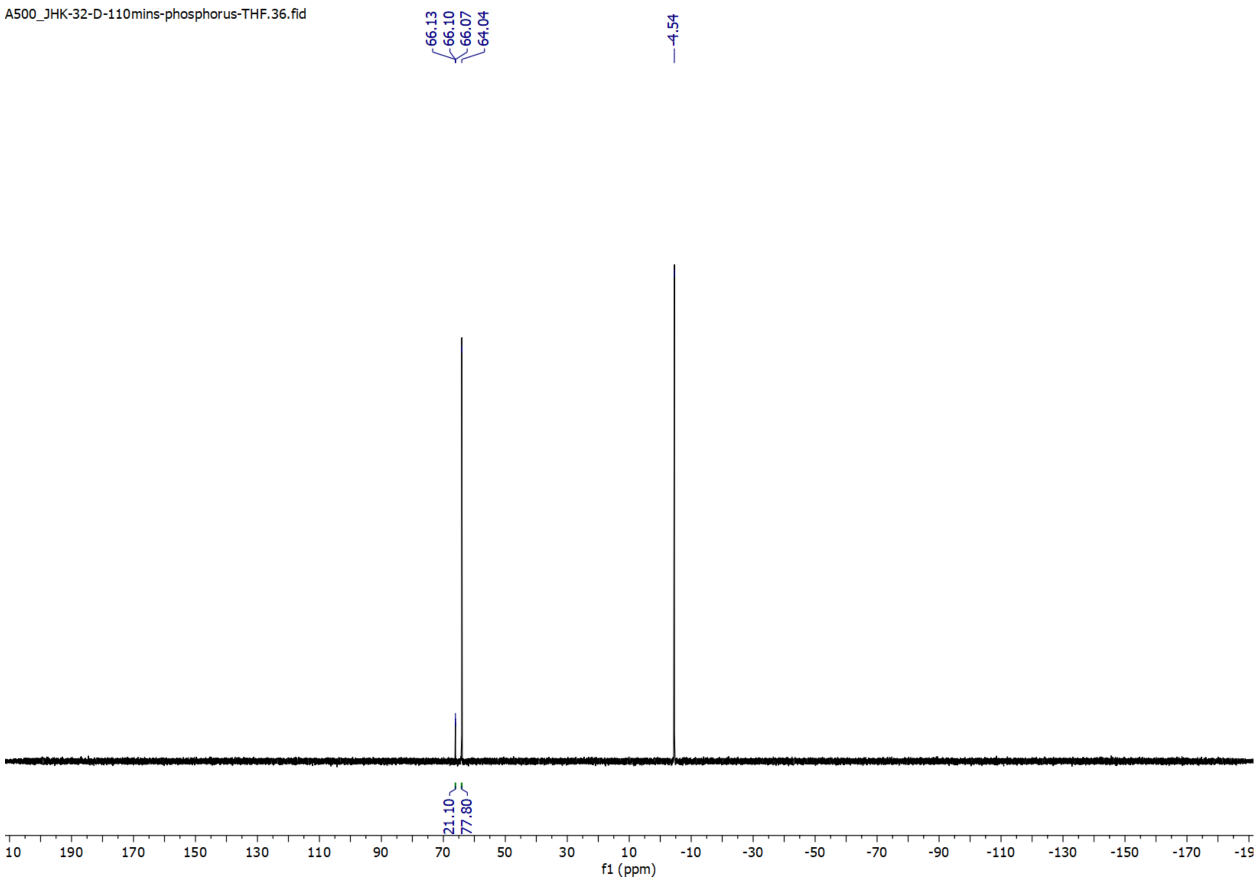

Figure S34. $^{31}P\{^1H\}$ NMR spectrum of a mixture of **Mo1** and 15 equiv Ph_2SiD_2 at 60 °C for 110 min (KIE experiment, run 1) in THF with a capillary filled with benzene-d_6.

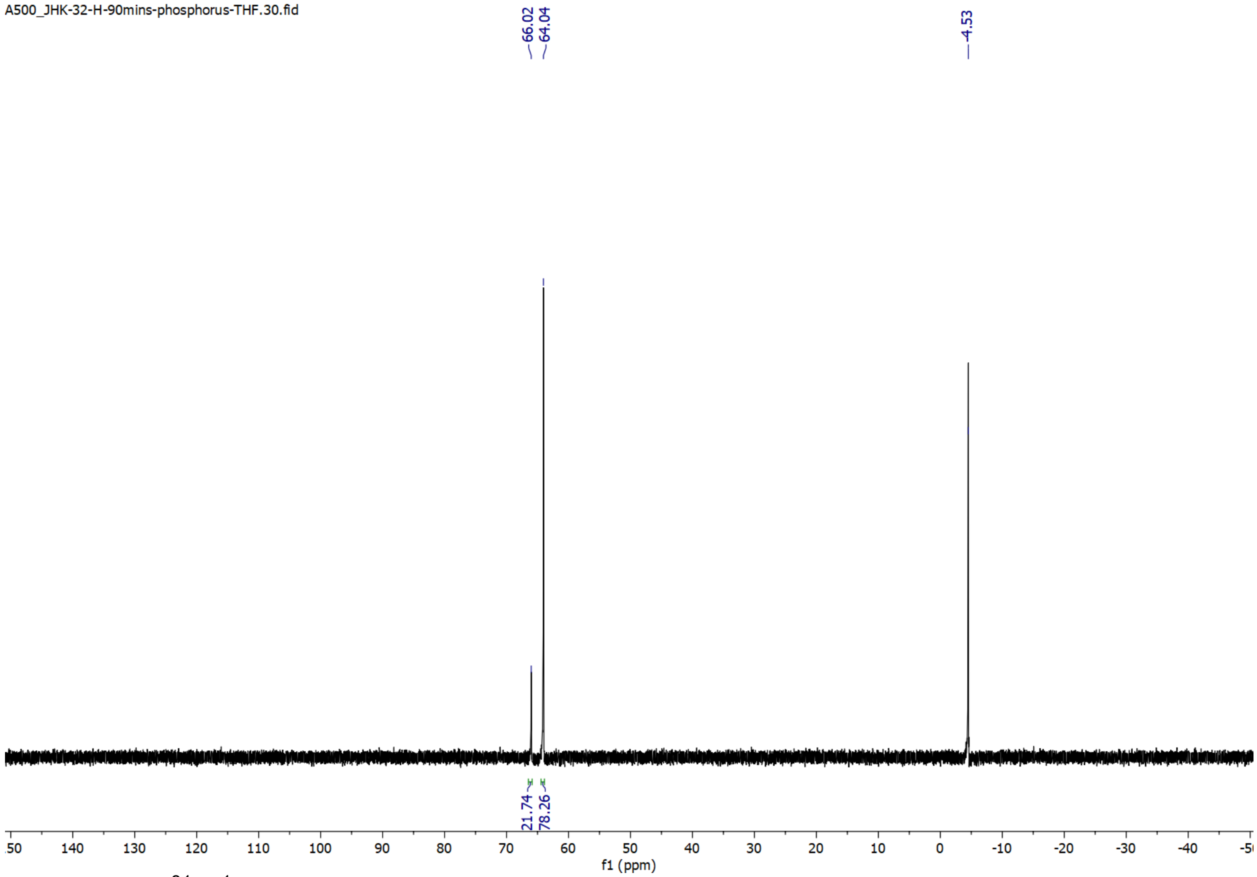

Figure S35. $^{31}P\{^1H\}$ NMR spectrum of a mixture of **Mo1** and 15 equiv Ph_2SiH_2 at 60 °C for 90 min (KIE experiment, run 2) in THF with a capillary filled with benzene-d_6.

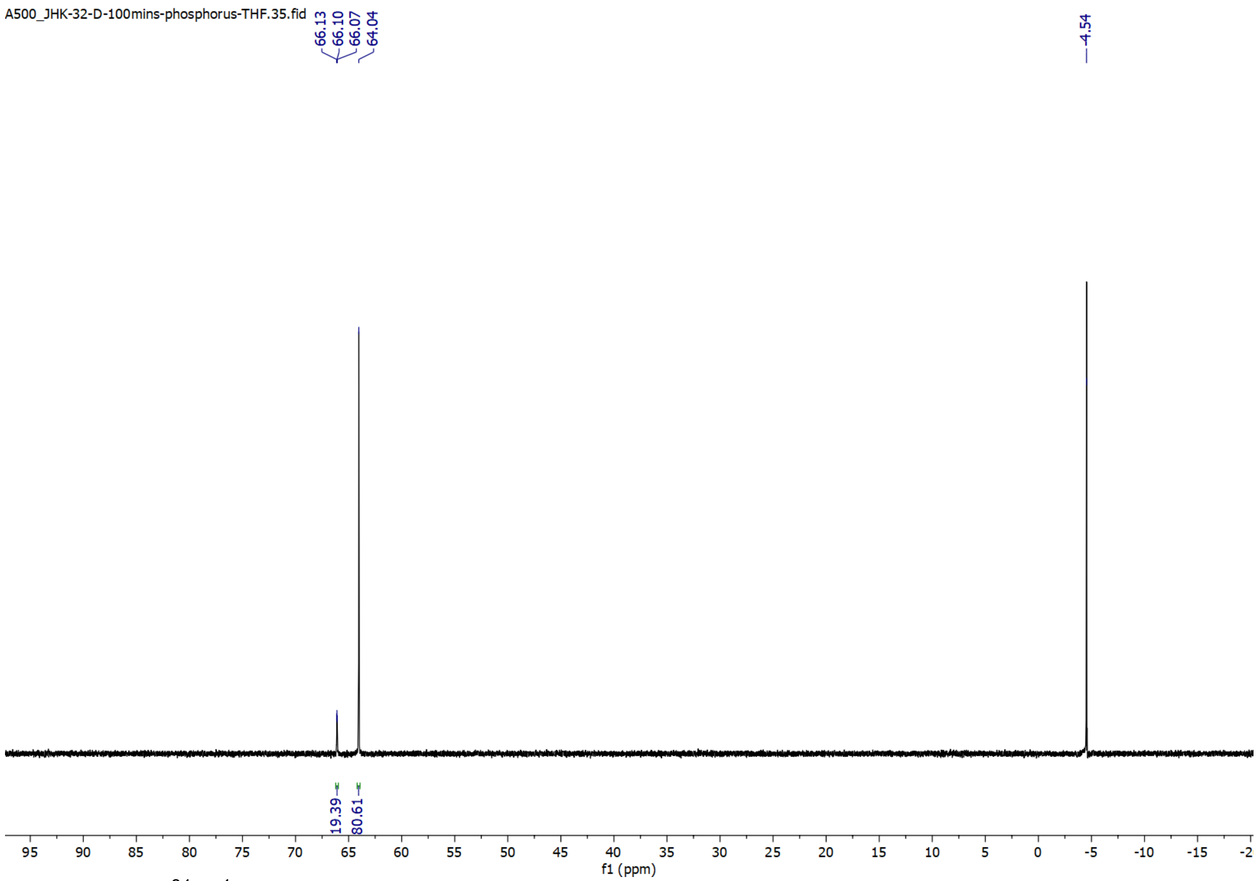

Figure S36. $^{31}P\{^1H\}$ NMR spectrum of a mixture of **Mo1** and 15 equiv Ph_2SiD_2 at 60 °C for 100 min (KIE experiment, run 2) in THF with a capillary filled with benzene-d_6.

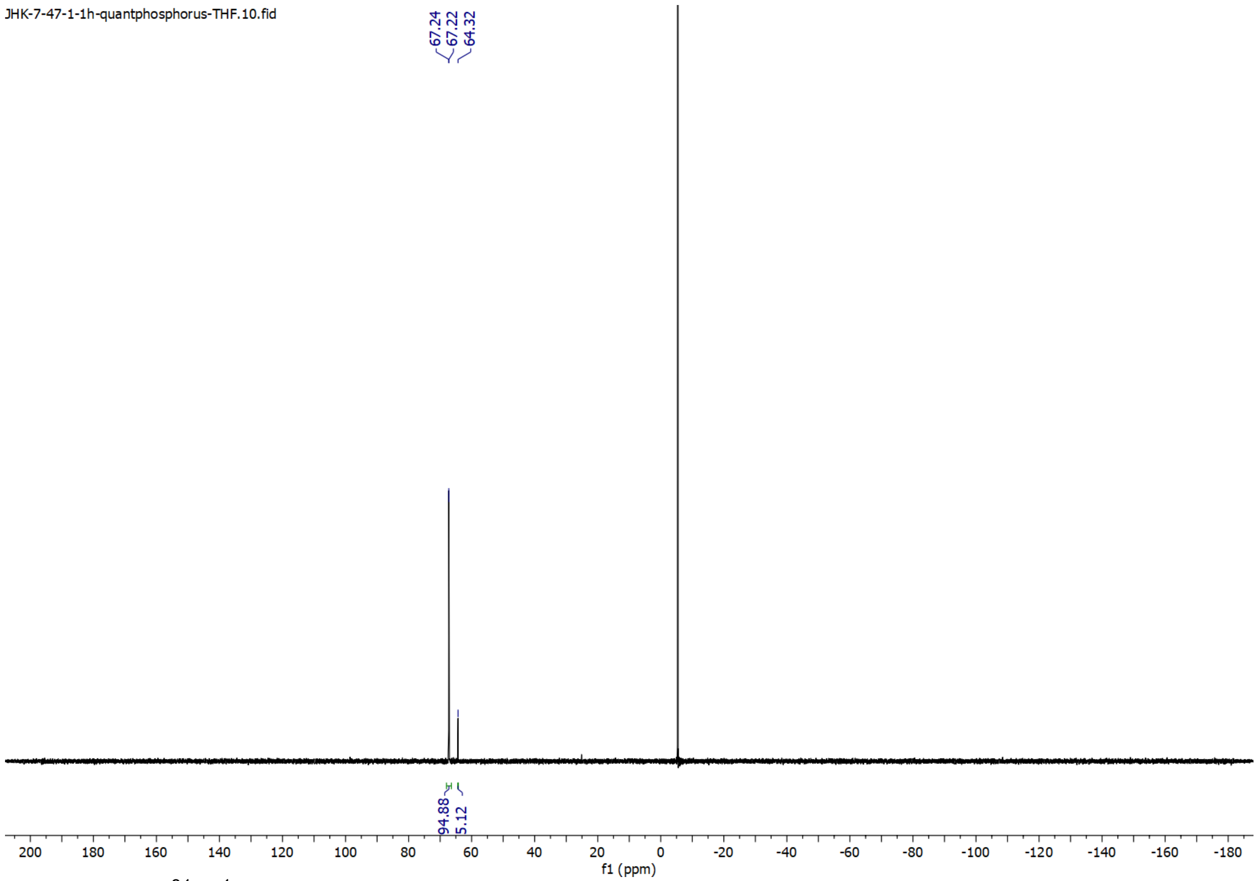

Figure S37. $^{31}P\{^1H\}$ NMR spectrum of a mixture of **Mo1** and 10 equiv $PhSiH_3$ at ambient temperature for 18 h in THF with a capillary filled with benzene-d_6.

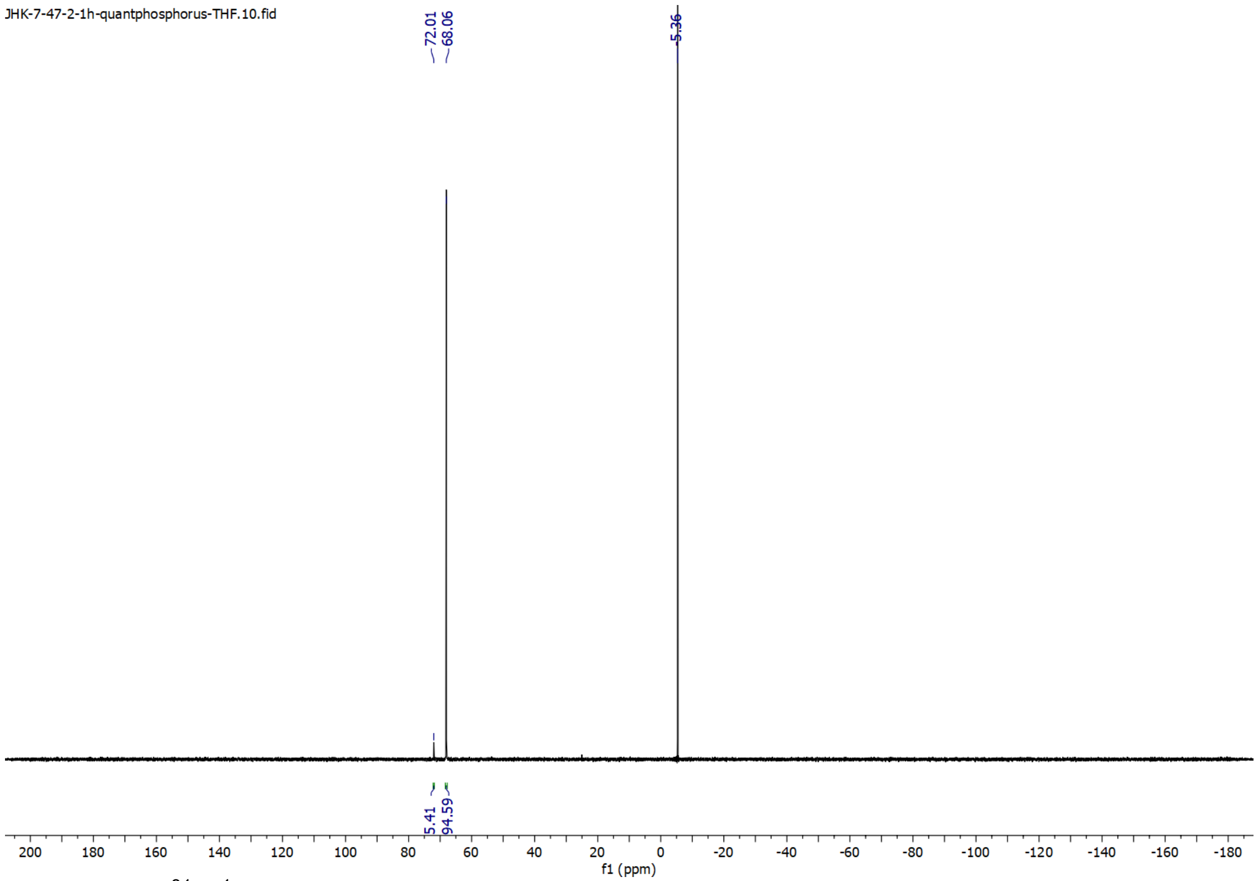

Figure S38. $^{31}P\{^1H\}$ NMR spectrum of a mixture of **Mo8** and 20 equiv $PhSiH_3$ at 60 °C for 18 h in THF with a capillary filled with benzene-d_6.

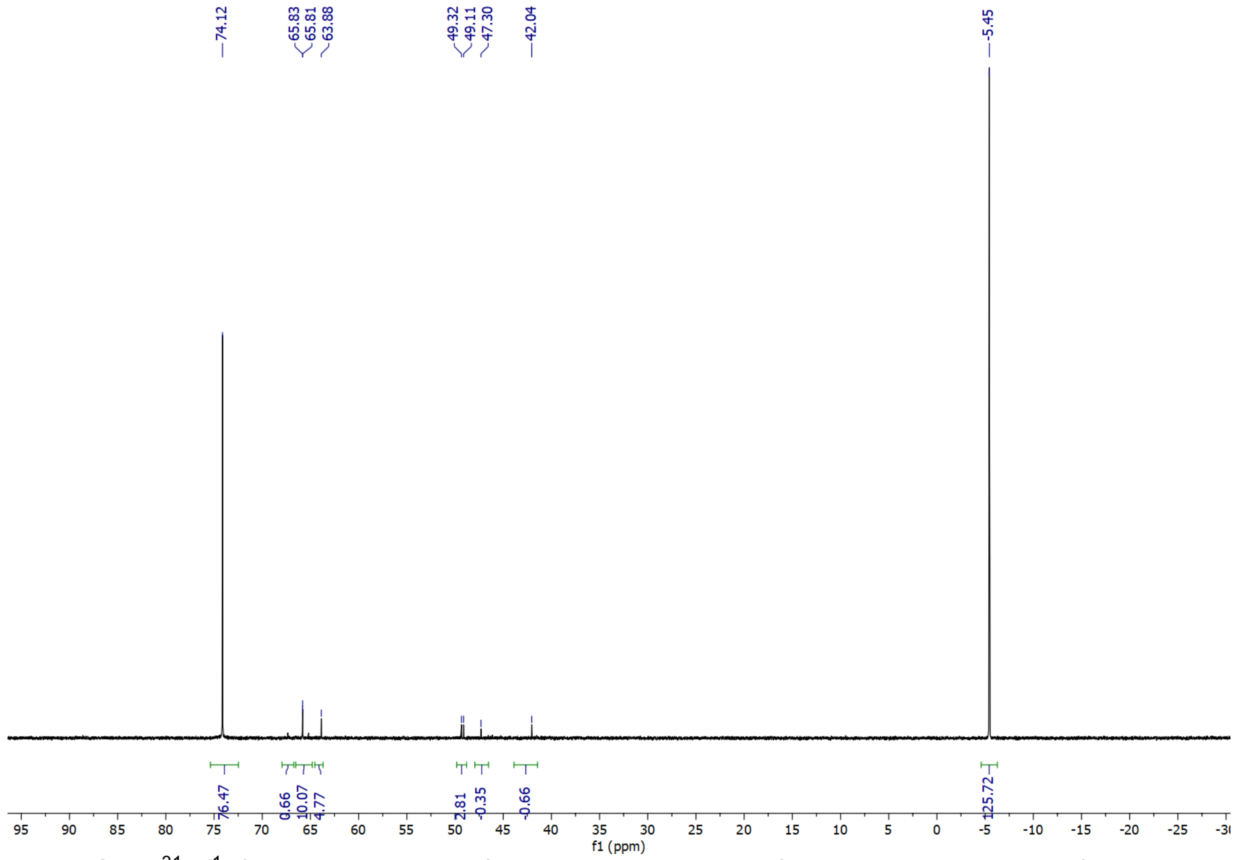

Figure S39. $^{31}P\{^{1}H\}$ NMR spectrum of a nonvolatile residue from a hydrogenation of **Mo4** in THF-d_8.

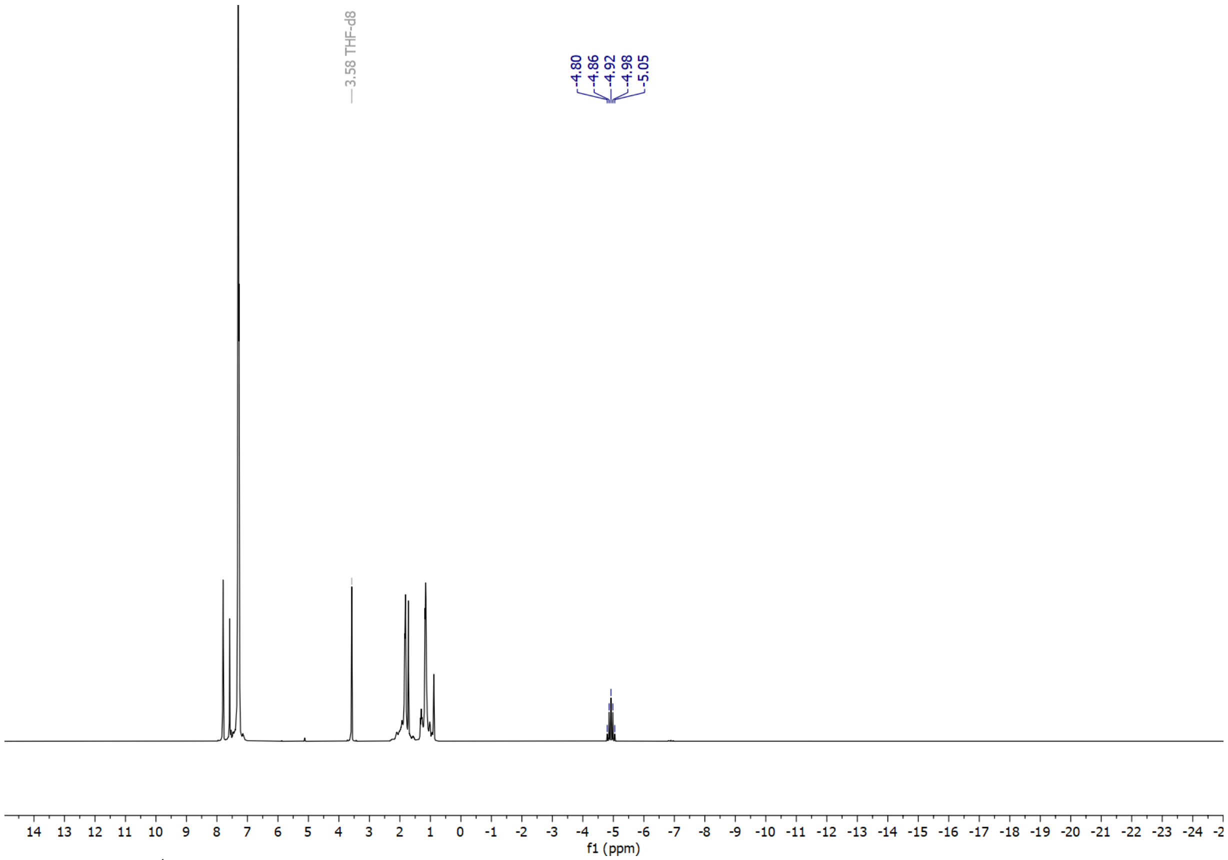

Figure S40. ^{1}H NMR spectrum of a nonvolatile residue from a hydrogenation of **Mo4** in THF-d_8.

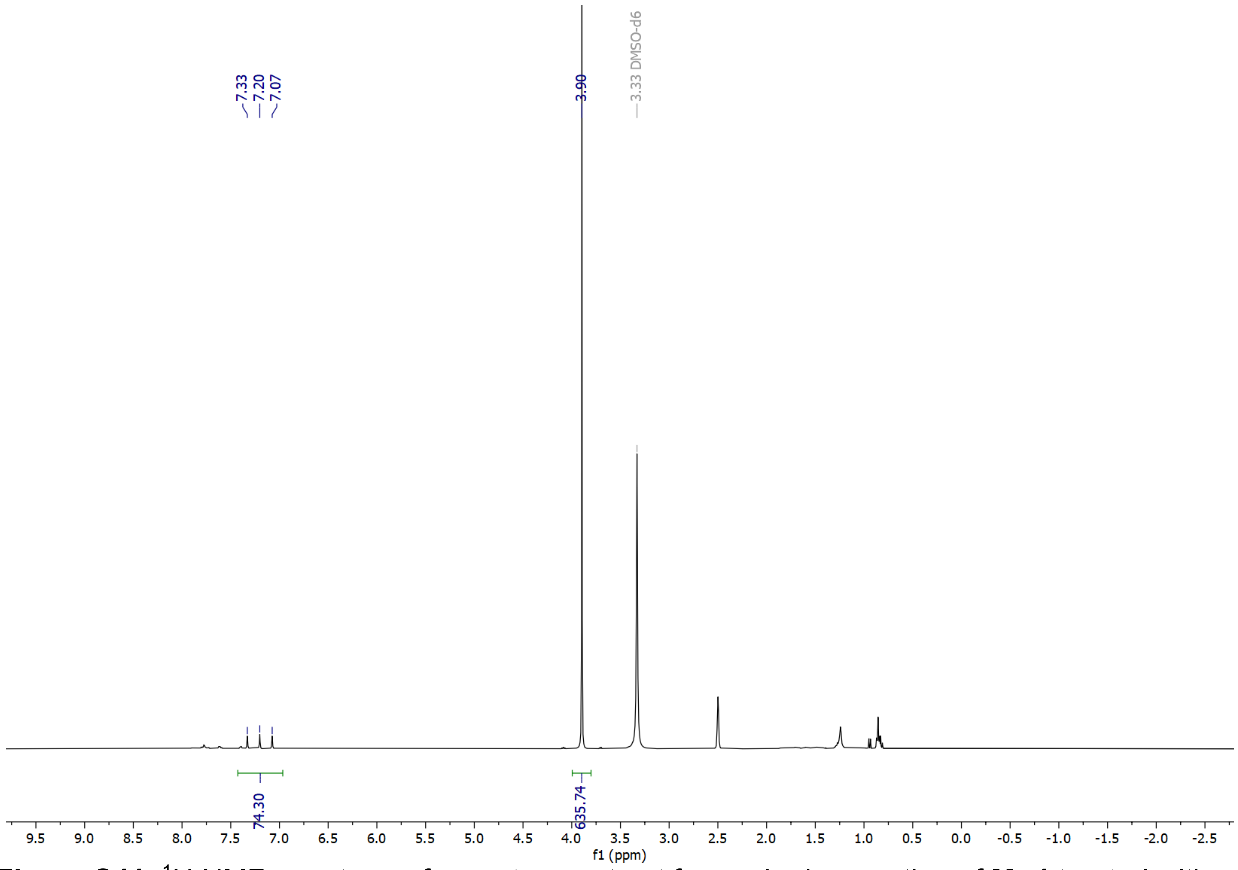

Figure S41. ^{1}H NMR spectrum of a pentane extract from a hydrogenation of **Mo4** treated with HCl and water in DMSO-d_8.

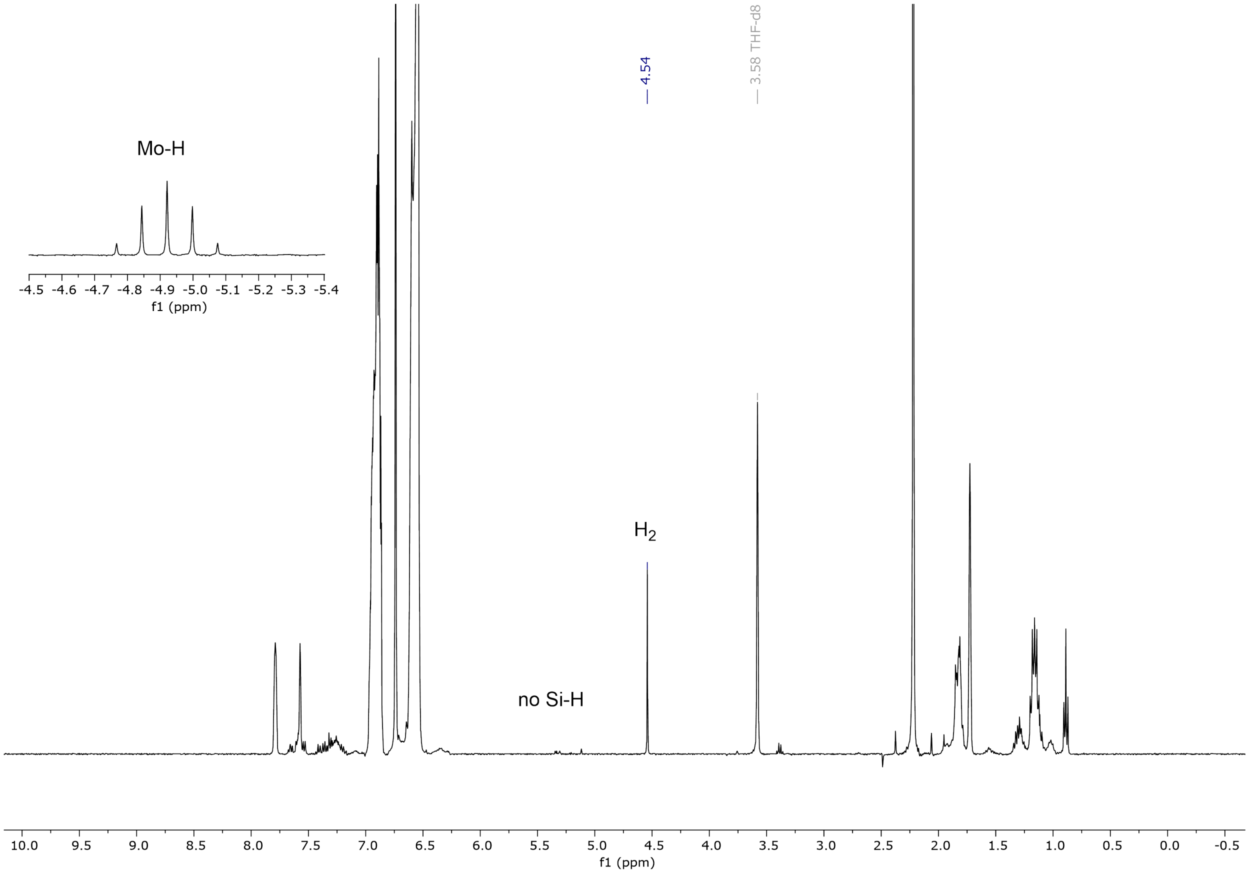

Figure S42. ^{1}H NMR spectrum of a crude mixture of hydrogenation of **Mo4** in THF-d_8 monitored in J. Young tube after the completion of the reaction (72 h).

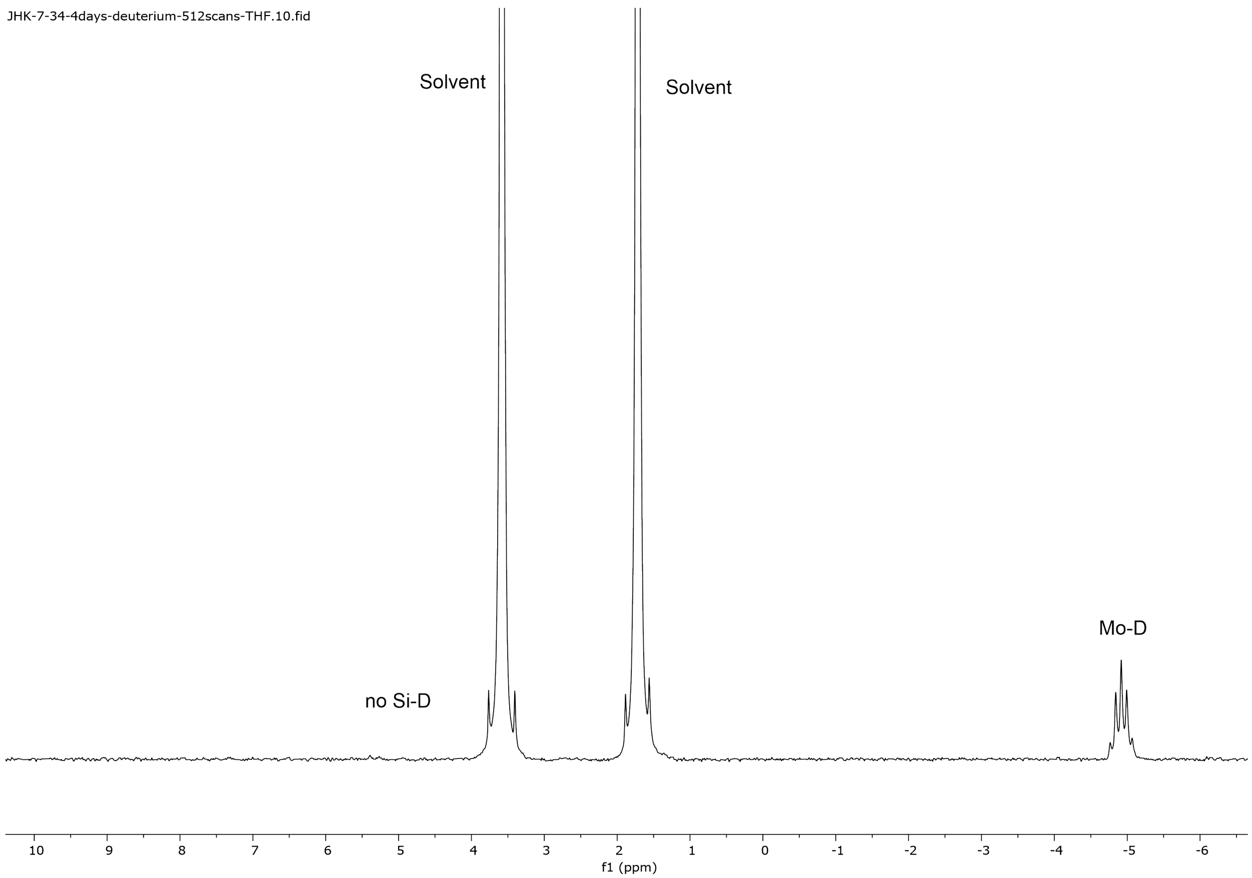

Figure S43. ^{2}H NMR spectrum of a crude mixture of hydrogenation of **Mo4** in THF monitored in J. Young tube after the completion of the reaction (72 h).

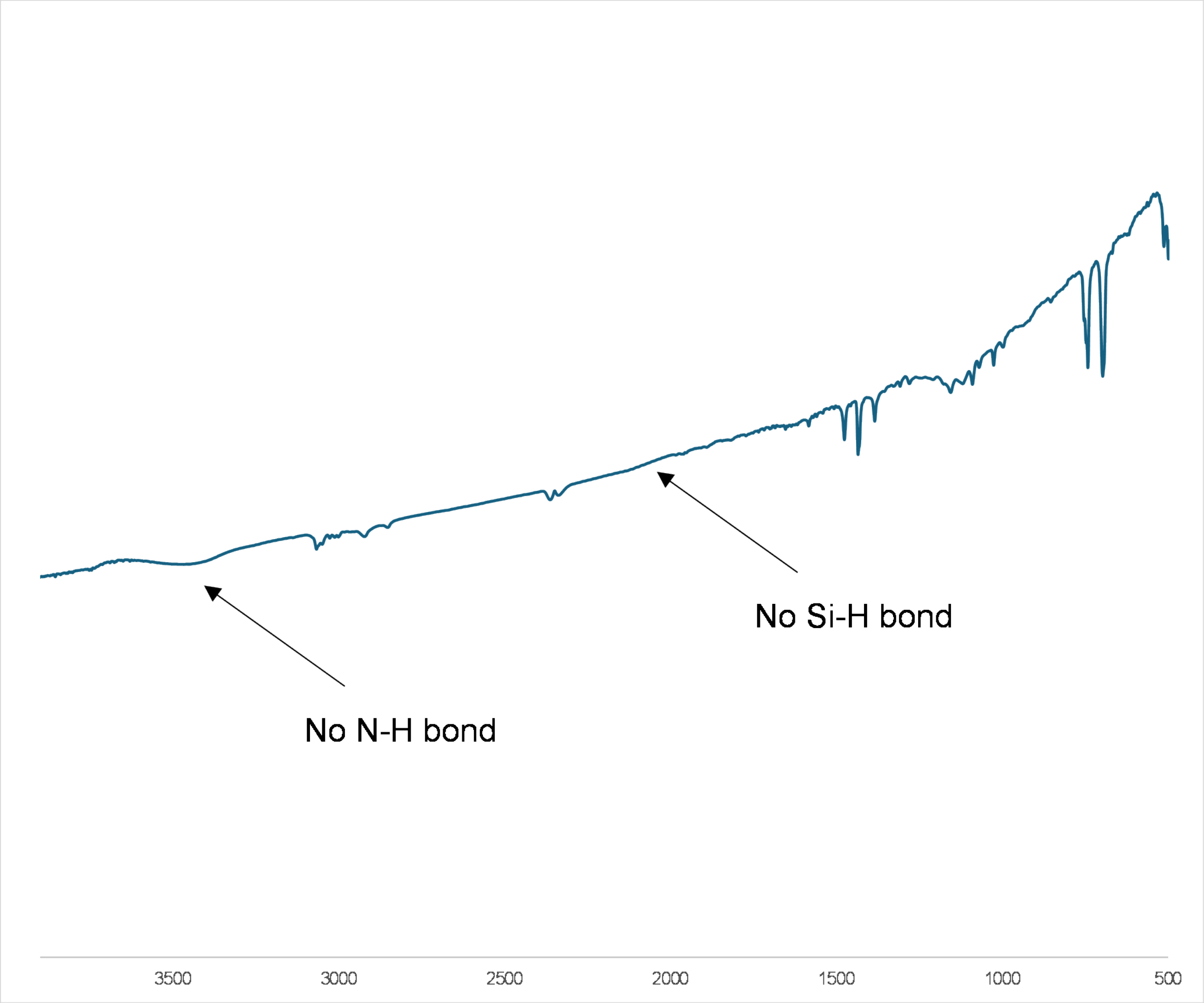

Figure S44. Solid-state FT-IR spectrum of a pentane extract from a hydrogenation of **Mo4** in KBr.

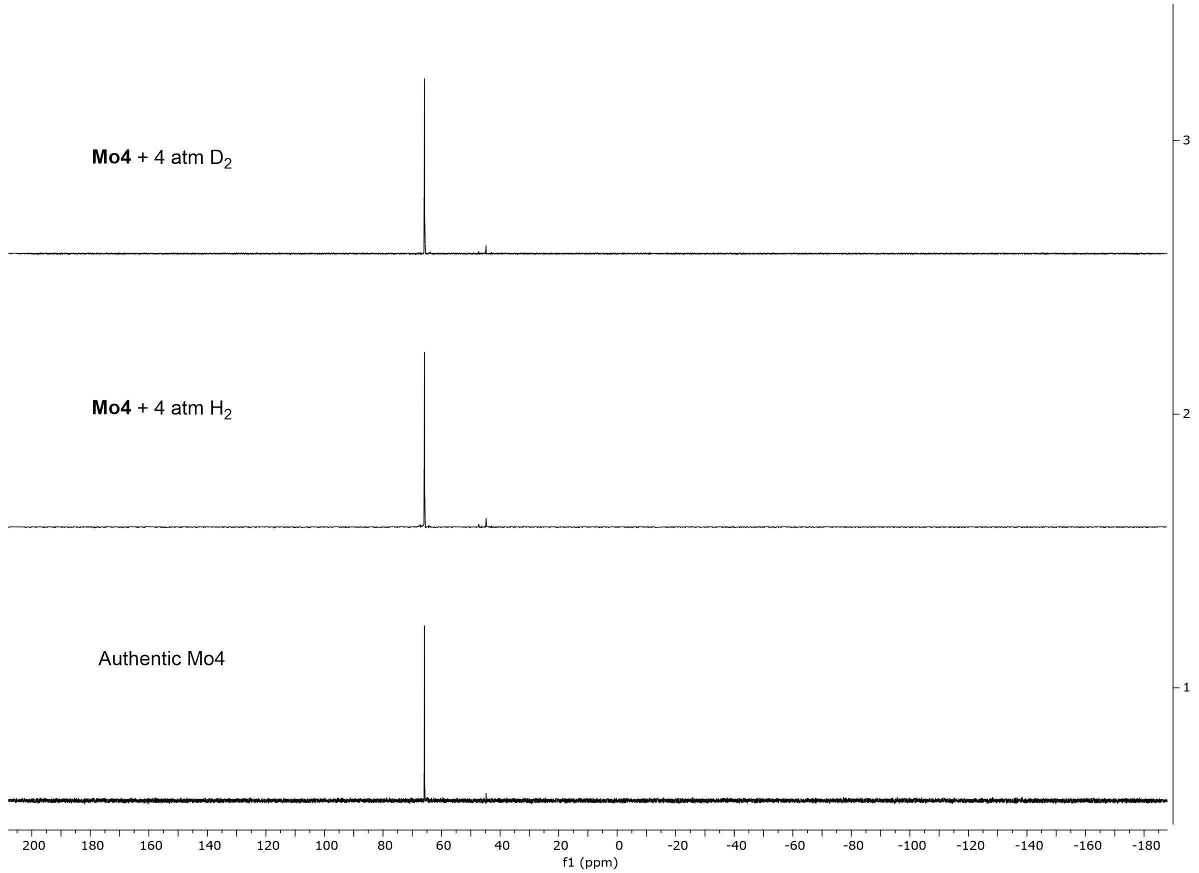

Figure S45. A stack of $^{31}P\{^{1}H\}$ NMR spectra of **Mo4**, **Mo4** with 4 atm H_2 and **Mo4** with 4 atm D_2 in THF-d_8.

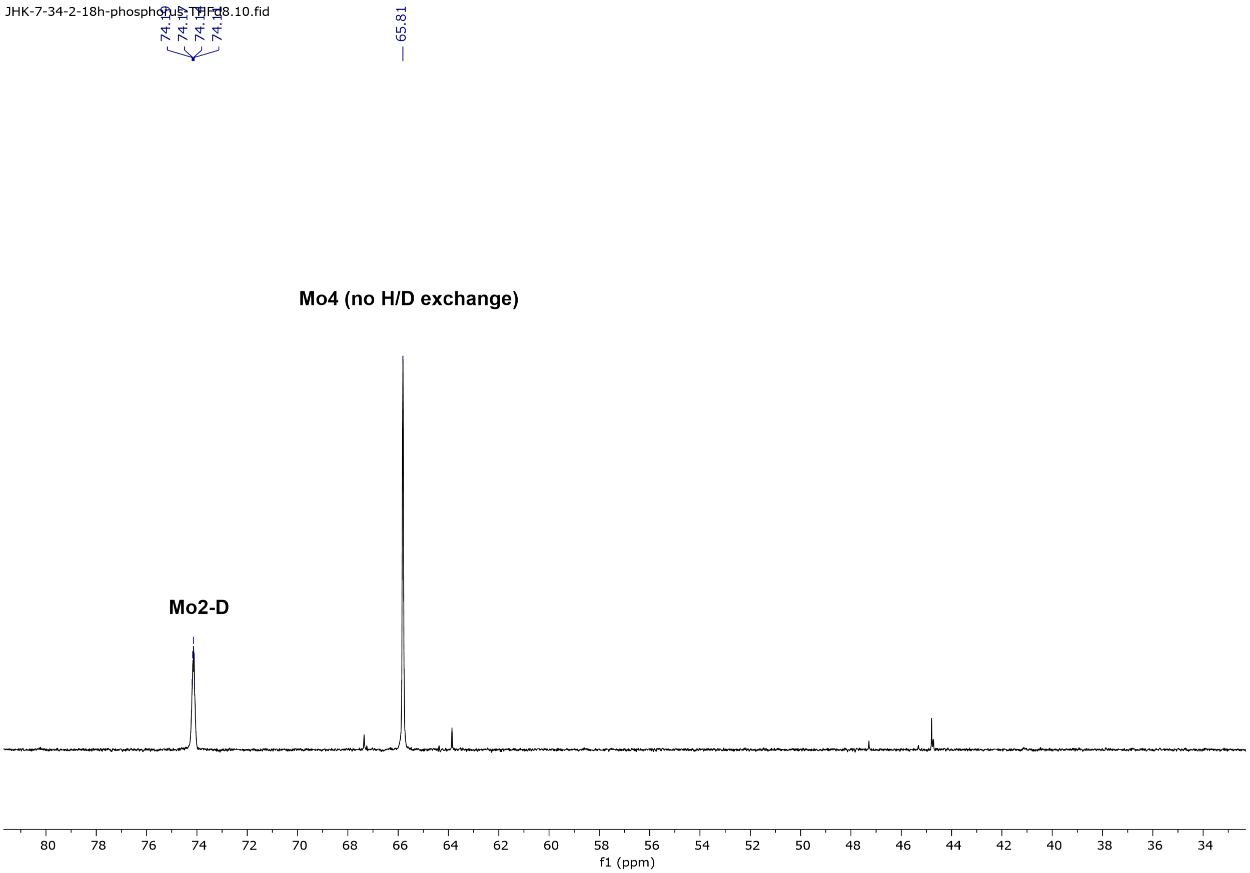

Figure S46. $^{31}P\{^{1}H\}$ spectrum of **Mo4** with 4 atm D_2 irradiated with blue LED for 18 h in THF-d_8.

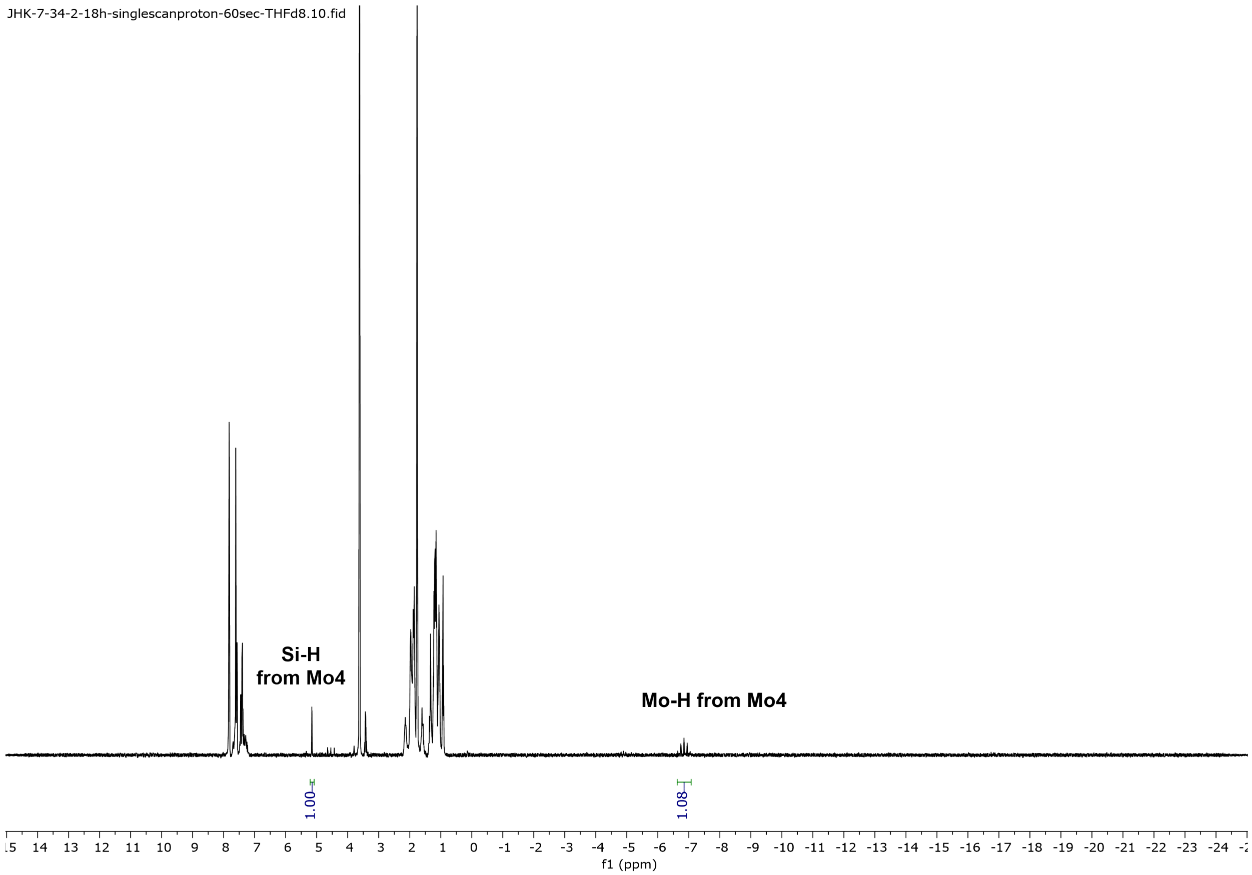

Figure S47. ^{1}H NMR spectrum of **Mo4** with 4 atm D_2 irradiated with blue LEDs for 18 h in THF-d_8.

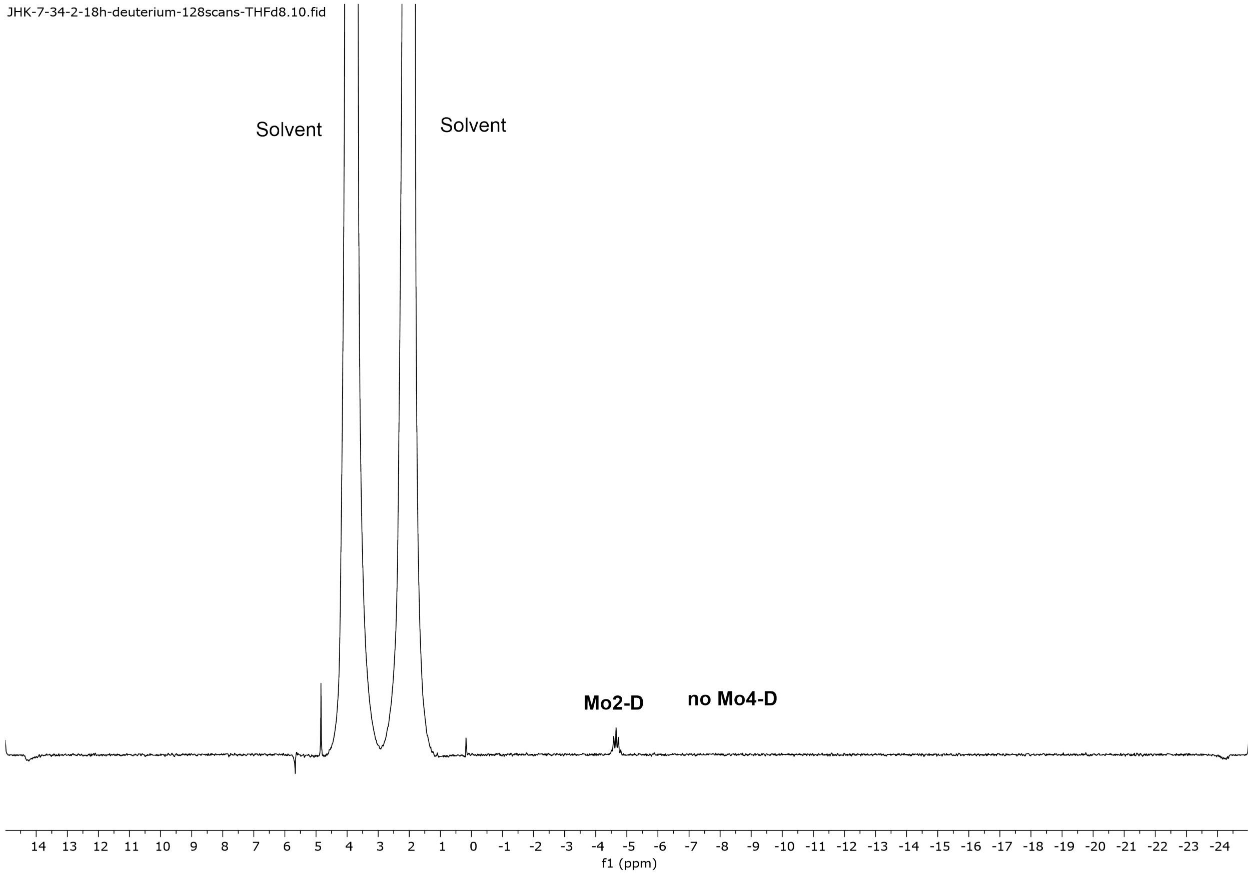

Figure S48. ^{2}H NMR spectrum of **Mo4** with 4 atm D_2 irradiated with blue LEDs for 18 h in THF-d_8.

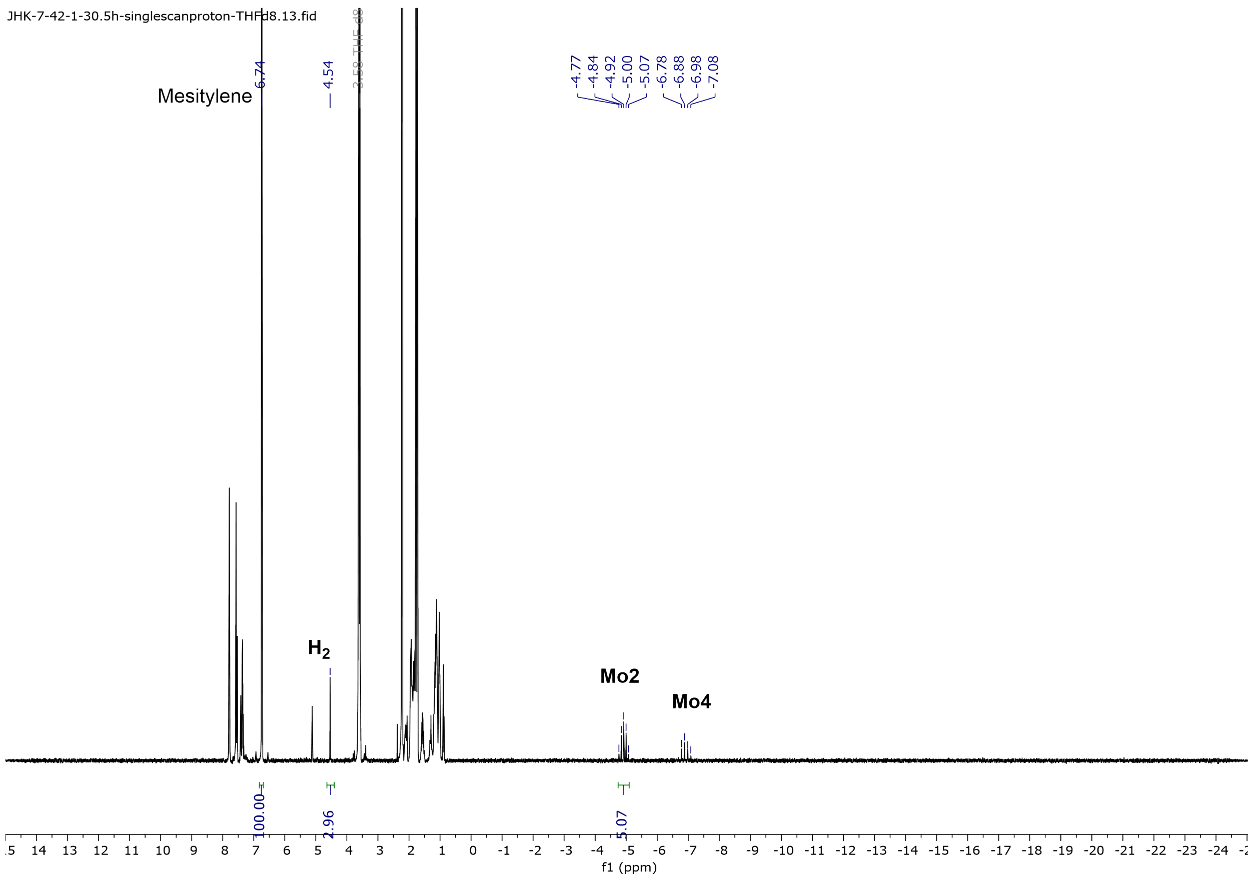

Figure S49. $^{31}P\{^{1}H\}$ NMR spectrum of hydrogenation of **Mo4** with 0.5 atm H_2 for 30.5 h monitored using J. Young tube in THF-d_8. Mesitylene was used as an internal standard.

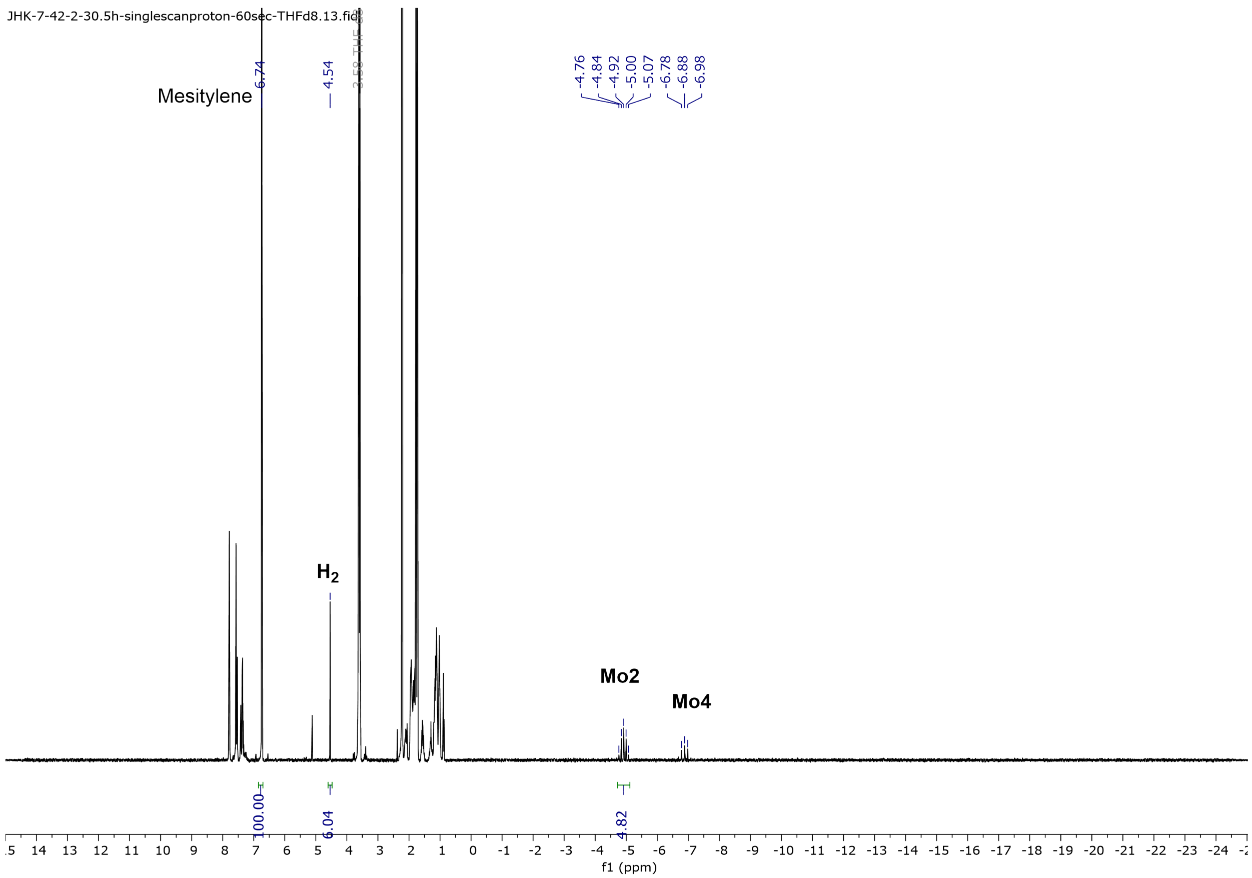

Figure S50. $^{31}P\{^1H\}$ NMR spectrum of hydrogenation of **Mo4** with 1 atm H_2 for 30.5 h monitored using J. Young tube in THF-d_8. Mesitylene was used as an internal standard.

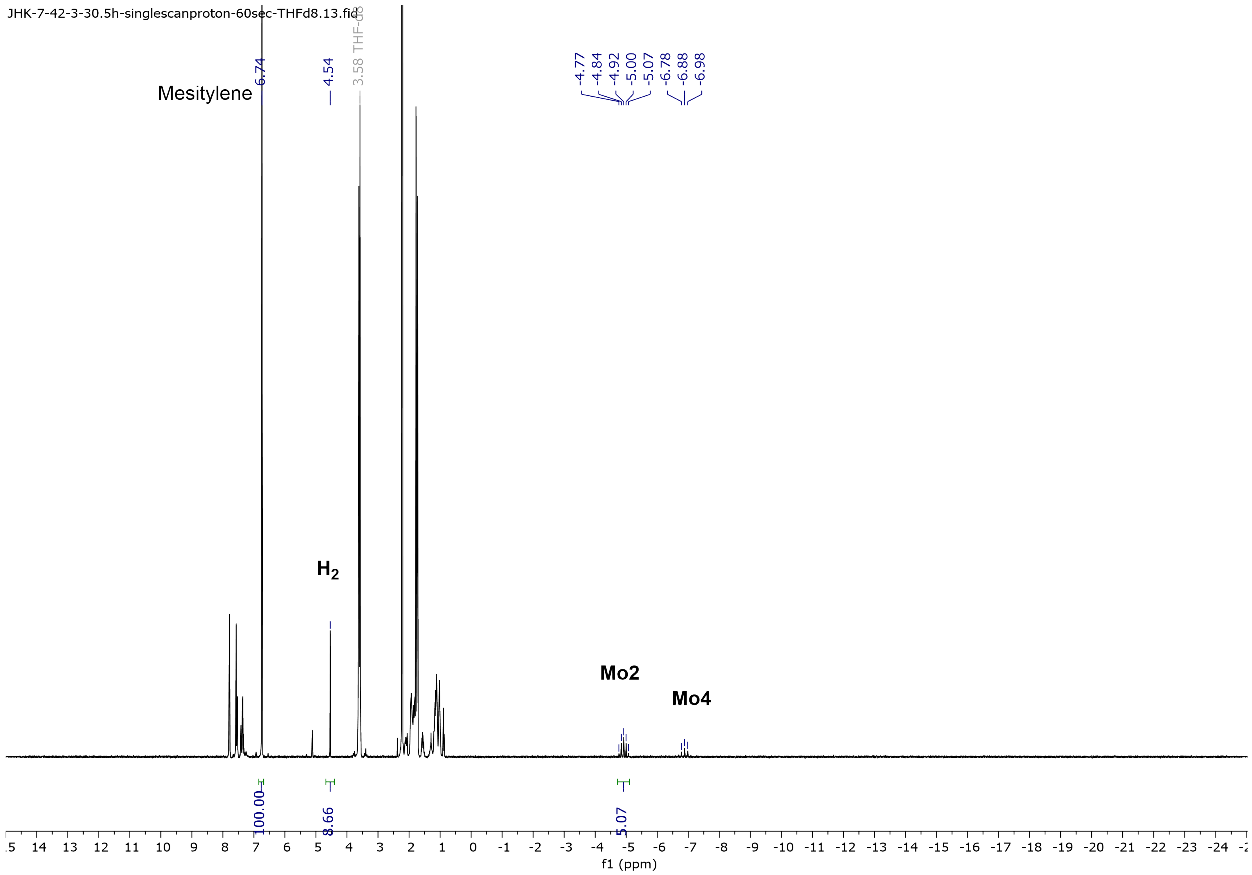

Figure S51. $^{31}P\{^{1}H\}$ NMR spectrum of hydrogenation of **Mo4** with 2 atm H_2 for 30.5 h monitored using J. Young tube in THF-d_8. Mesitylene was used as an internal standard.

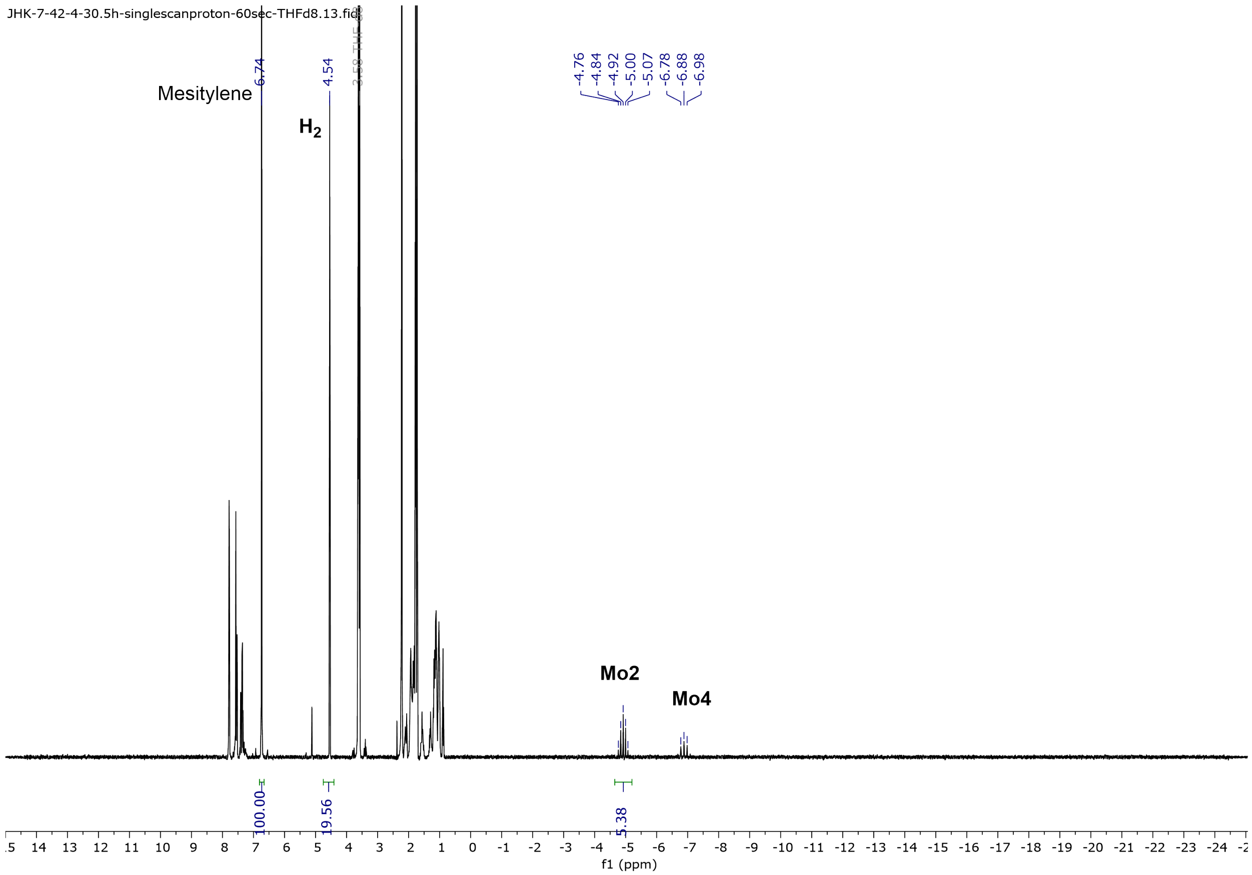

Figure S52. $^{31}P\{^1H\}$ NMR spectrum of hydrogenation of **Mo4** with 4 atm H_2 for 30.5 h monitored using J. Young tube in THF-d_8. Mesitylene was used as an internal standard.

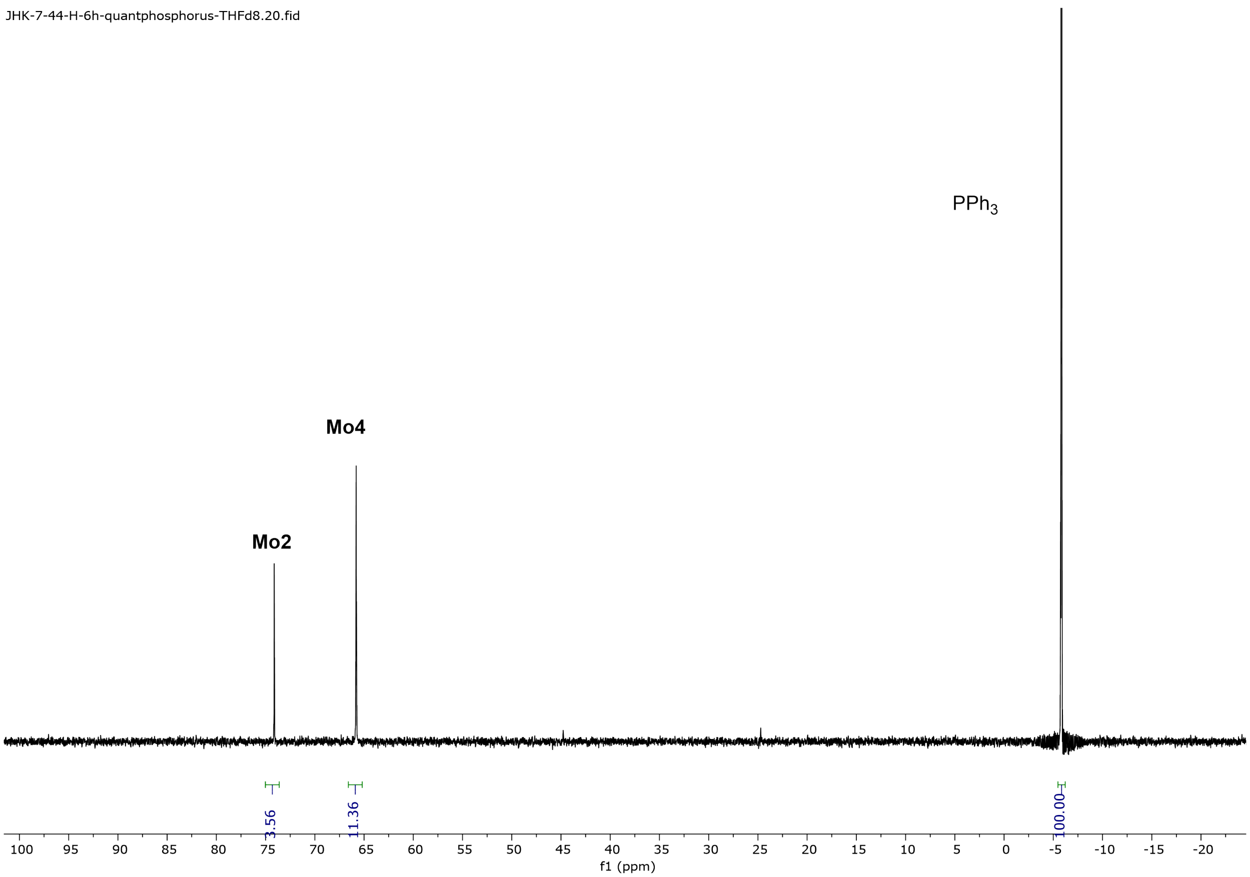

Figure S53. $^{31}P\{^{1}H\}$ NMR spectrum of hydrogenation of **Mo4** with 4 atm H_2 for 6 h monitored using J. Young tube in THF-d_8. PPh_3 in benzene-d_6 capillary was used as an external standard (KIE experiment).

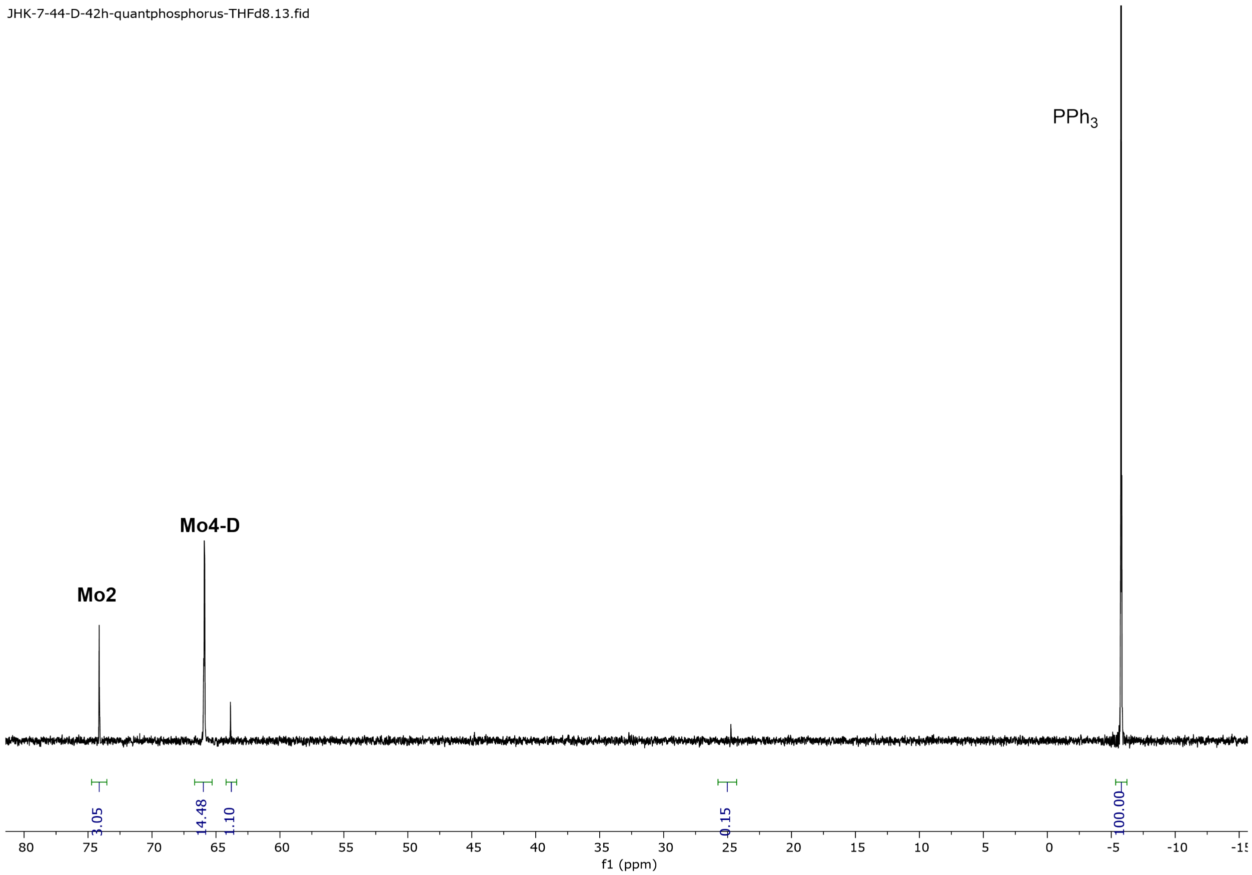

Figure S54. $^{31}P\{^{1}H\}$ NMR spectrum of hydrogenation of **Mo4-D** with 4 atm H_2 for 42 h monitored using J. Young tube in THF-d_8. PPh_3 in a benzene-d_6 capillary was used as an external standard (KIE experiment).

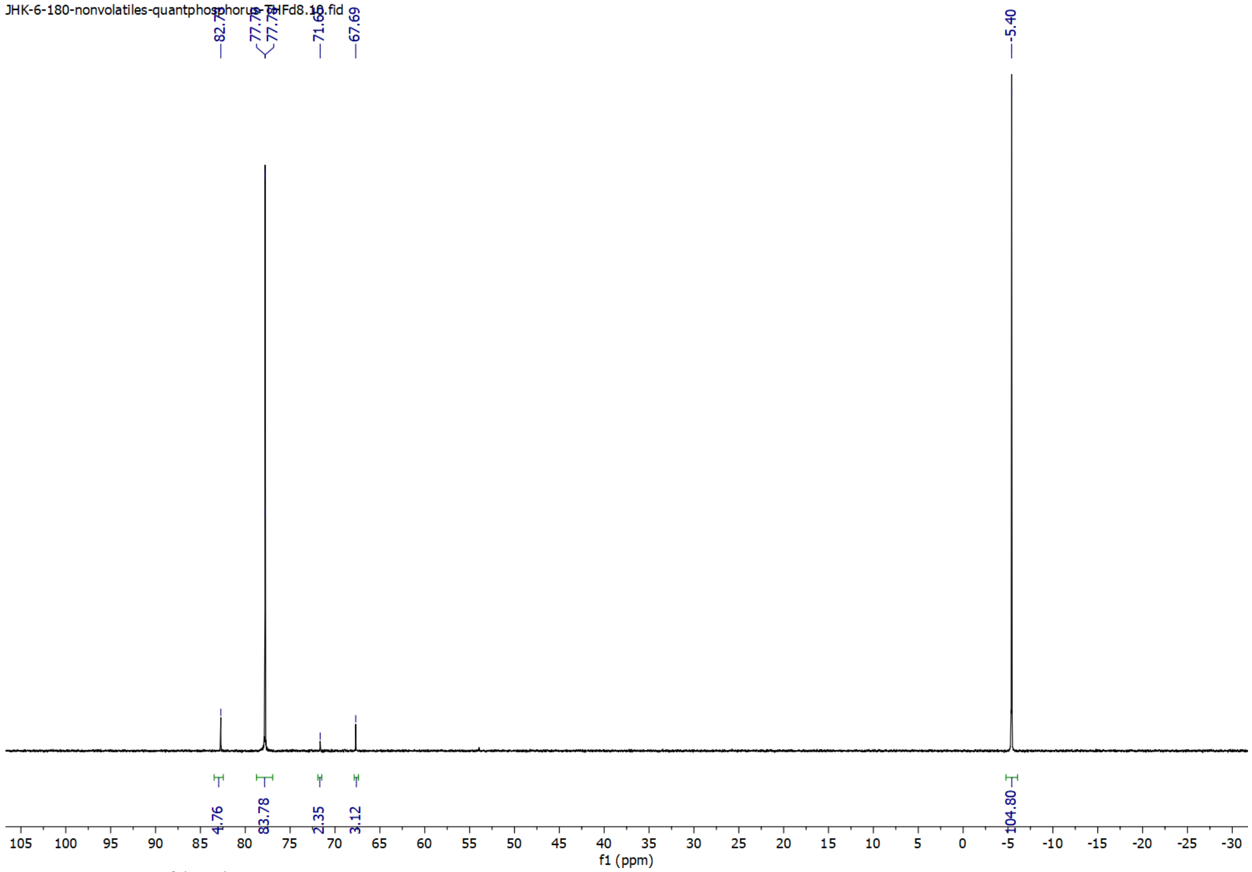

Figure S55. $^{31}P\{^{1}H\}$ NMR spectrum of a nonvolatile residue from a hydrogenation of **Mo9** in THF-d_8.

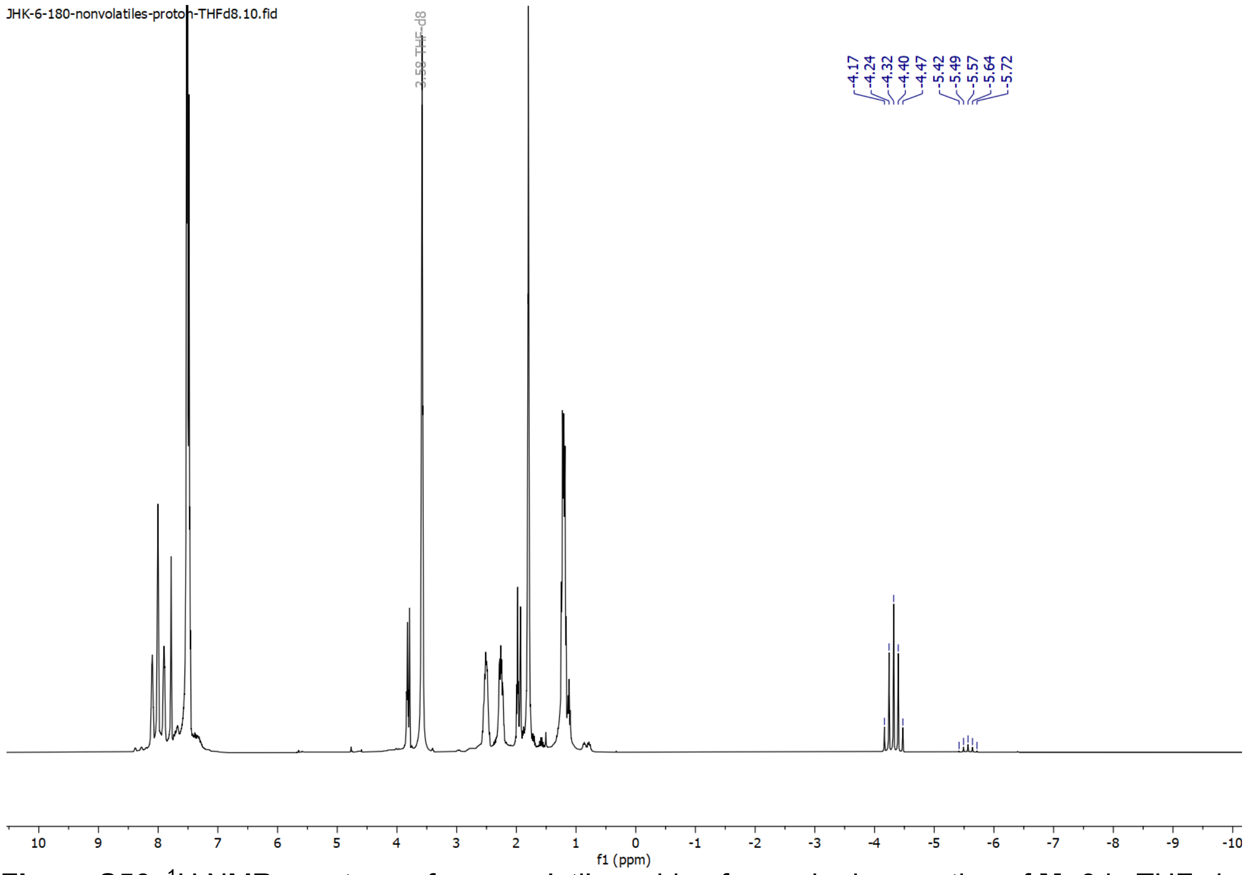

Figure S56. ^{1}H NMR spectrum of a nonvolatile residue from a hydrogenation of **Mo9** in THF-d_8.

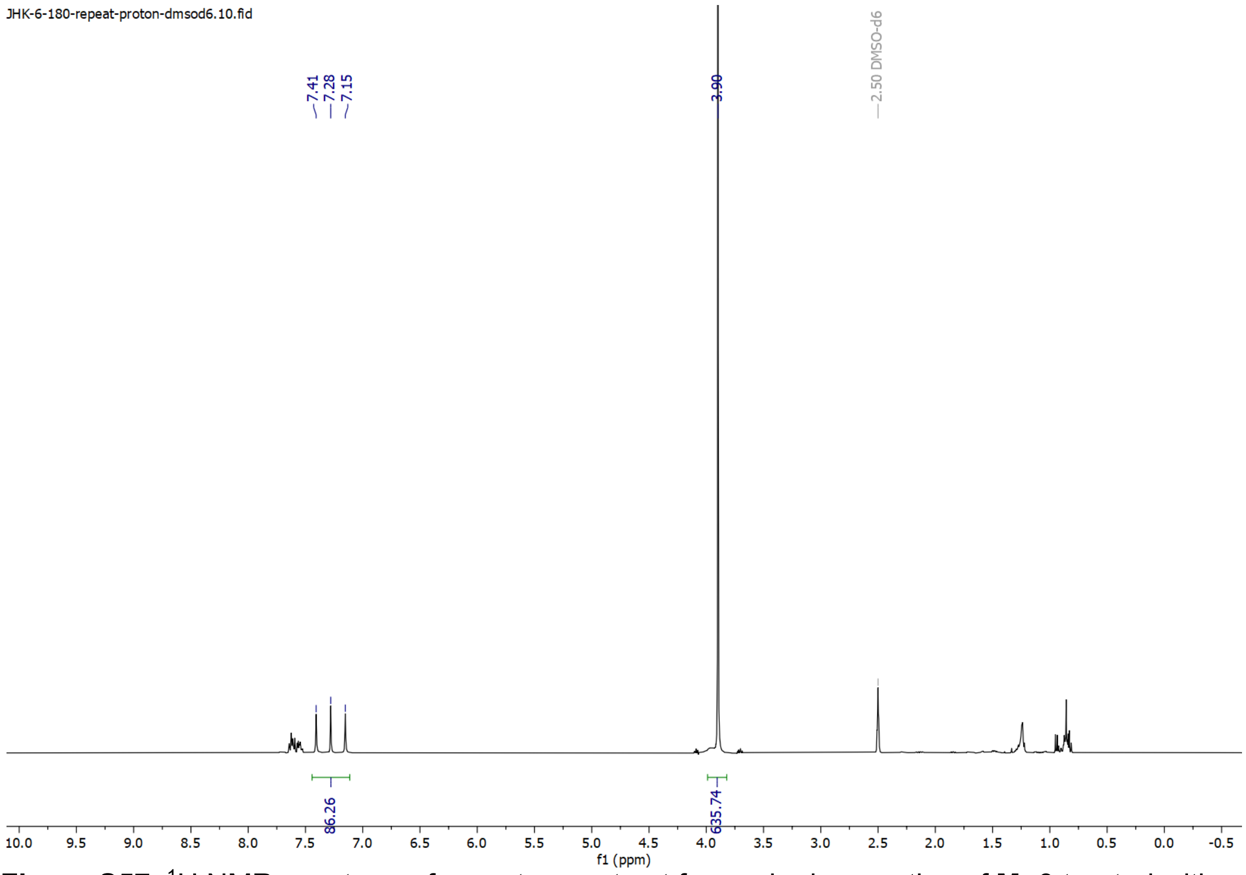

Figure S57. ^{1}H NMR spectrum of a pentane extract from a hydrogenation of **Mo9** treated with HCl and water in DMSO-d_6.

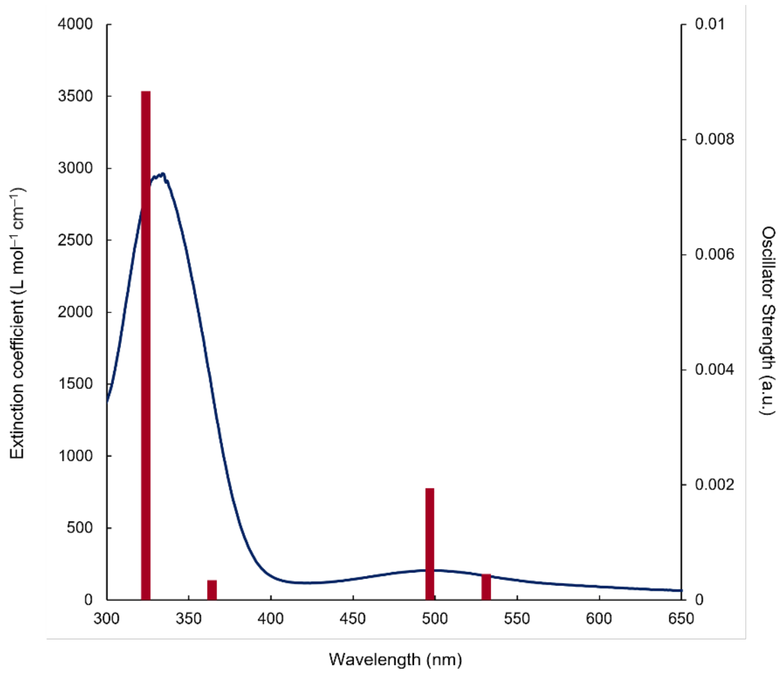

Figure S58. Electronic absorption spectrum (blue line) and TD-DFT simulated vertical transitions (maroon sticks) of **Mo4** in THF at room temperature.

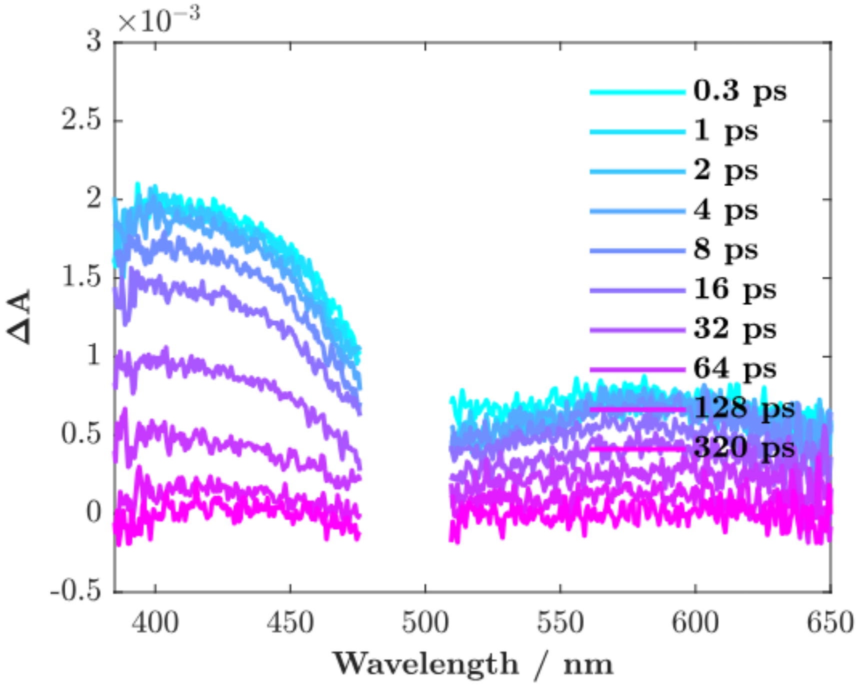

Figure S59. TA spectrum of **Mo4** in THF (10 mM) under room temperature. λ_{pump} = 490 nm

VI. Computational Data

Coordinates of optimized geometries.

Mo1

6	-3.140140000	1.102810000	-3.977349000
6	-2.167907000	-2.255789000	-3.913312000
6	-0.020373000	-0.017935000	-3.390507000
6	1.120452000	-0.986115000	-3.119254000
6	-2.534809000	1.142370000	-2.587017000
6	-2.181816000	-1.768183000	-2.476613000
6	2.778321000	1.541282000	-1.618399000
6	3.111492000	0.089769000	-1.329774000
6	2.367338000	-2.663290000	-1.129480000
6	1.362926000	-3.797564000	-1.113648000
6	-0.910994000	3.504450000	0.159317000
6	-2.156569000	2.843944000	0.718169000
6	-3.484771000	0.551615000	1.828789000
6	-3.477112000	-0.694281000	2.691383000
6	2.199103000	1.686377000	2.120459000
6	2.139523000	-1.164045000	2.850820000
6	-1.092444000	1.611668000	3.085984000
6	2.429550000	2.461723000	3.405495000
6	-0.080946000	0.574228000	3.547629000
6	2.848813000	-0.964893000	4.176299000
1	-2.380399000	1.080409000	-4.757859000
1	-3.755072000	1.984434000	-4.152911000
1	-3.782305000	0.234071000	-4.110466000
1	-0.431816000	-0.169421000	-4.390065000
1	-2.606867000	-1.544373000	-4.609409000
1	-1.156186000	-2.467780000	-4.259088000
1	1.980067000	-0.780923000	-3.761194000
1	0.328868000	1.017852000	-3.353103000
1	-2.731398000	-3.183952000	-3.998934000
1	0.801772000	-2.005242000	-3.351042000
1	2.472275000	1.684385000	-2.654511000
1	-2.012476000	2.089399000	-2.421585000
1	3.845161000	-0.292120000	-2.044107000
1	3.633559000	2.192432000	-1.444586000
1	-3.321603000	1.106479000	-1.828708000
1	-1.729808000	-2.507235000	-1.814382000
1	-3.204952000	-1.645530000	-2.111242000
1	3.103384000	-2.795191000	-1.927680000
1	0.816972000	-3.870658000	-2.055361000
1	1.962432000	1.899504000	-0.983368000
1	3.559456000	-0.015790000	-0.338357000
1	1.865345000	-4.751496000	-0.964143000
1	2.929638000	-2.635358000	-0.193627000
1	-0.430063000	2.869174000	-0.591468000
1	-2.902151000	2.705447000	-0.066950000
1	0.632343000	-3.669367000	-0.315621000
1	-1.138107000	4.456424000	-0.317856000
1	-3.975246000	0.358233000	0.871618000
1	-0.173462000	3.705593000	0.938137000
1	-2.625524000	3.468884000	1.482224000
1	-2.888265000	-1.490010000	2.235344000
1	3.147033000	1.336488000	1.702515000
1	-4.040392000	1.361562000	2.309866000
1	-4.489350000	-1.065932000	2.839058000
1	1.780974000	2.349540000	1.359308000
1	2.856858000	-1.396422000	2.059333000
1	1.463575000	-2.022513000	2.886109000
1	3.134153000	3.275017000	3.237518000
1	-0.606919000	2.585994000	2.980195000
1	-3.064142000	-0.492835000	3.680205000
1	-1.891021000	1.747157000	3.818370000
1	1.508580000	2.910096000	3.777156000
1	-0.575286000	-0.373506000	3.777135000
1	2.832368000	1.840476000	4.202247000
1	3.332702000	-1.888217000	4.491447000
1	3.625171000	-0.204294000	4.110943000
1	0.430680000	0.897719000	4.455893000
1	2.161850000	-0.675657000	4.970887000
42	-0.269770000	-0.350884000	0.175591000
7	-0.811260000	-1.800066000	0.710710000
15	-1.339934000	-0.168630000	-2.107157000
15	1.634266000	-0.997495000	-1.337785000
15	-1.815159000	1.188473000	1.427689000
15	1.108667000	0.198257000	2.191805000

Final Gibbs free energy (Hartree/particle) = -6304.694909

Mo3

6	-0.881329000	-1.599169000	-1.771621000
6	-1.037547000	-2.070333000	-0.338822000
6	1.771574000	-1.514456000	-0.011849000
6	2.271828000	-2.948348000	-0.018423000
6	3.899269000	-5.716761000	0.646608000
6	-0.193899000	0.369525000	0.950239000
6	2.672172000	-5.382062000	1.473788000
6	-3.267723000	-2.116784000	2.480137000
6	0.807997000	1.179143000	1.749945000
6	3.872044000	-2.894969000	2.349097000
6	-4.691623000	-2.078414000	3.002112000
6	4.259563000	-3.412565000	3.719180000
6	-0.167398000	-6.278492000	3.595211000
6	-2.022040000	0.298430000	4.462167000
6	-2.527109000	-3.931186000	4.574700000
6	-0.225016000	-7.528787000	4.450767000
6	-2.245279000	-1.107834000	4.982104000
6	-1.423366000	-4.502464000	5.445043000
6	1.373299000	-5.095418000	5.744457000
6	1.704012000	-3.951516000	6.682258000
1	0.027705000	-1.983828000	-2.232039000
1	-1.715420000	-1.948761000	-2.378131000
1	-0.859941000	-0.512856000	-1.854520000
1	1.683293000	-1.107180000	-1.020454000
1	-0.995309000	-3.158572000	-0.270306000
1	1.609928000	-3.590146000	-0.606606000
1	3.264409000	-3.023940000	-0.465608000
1	3.826346000	-5.326503000	-0.367481000
1	2.474433000	-0.872995000	0.524589000
1	-2.019618000	-1.797961000	0.050936000

1	-0.268203000	0.744765000	-0.073331000
1	4.022970000	-6.795542000	0.566268000
1	4.812449000	-5.321985000	1.090093000
1	1.774284000	-5.819138000	1.030090000
1	-1.192818000	0.456948000	1.385400000
1	-3.143990000	-2.903765000	1.732659000
1	4.656794000	-3.089235000	1.613694000
1	-3.019550000	-1.180233000	1.973823000
1	-5.394074000	-1.911228000	2.187013000
1	1.774041000	1.234072000	1.249511000
1	3.732148000	-1.812608000	2.380217000
1	2.750398000	-5.815628000	2.474255000
1	0.460013000	2.201634000	1.885987000
1	0.972501000	0.745867000	2.737668000
1	-1.099327000	-6.126269000	3.046508000
1	-4.974887000	-3.014428000	3.481440000
1	-4.843271000	-1.276887000	3.724077000
1	-2.744274000	0.562421000	3.688524000
1	0.596756000	-6.370896000	2.824866000
1	4.434910000	-4.489079000	3.713306000
1	-1.023479000	0.405584000	4.035765000
1	-2.815887000	-4.644307000	3.798075000
1	5.175798000	-2.940102000	4.068901000
1	-0.529307000	-8.385905000	3.851973000
1	3.477832000	-3.200888000	4.449284000
1	-2.123538000	1.034546000	5.257836000
1	-0.941422000	-7.441328000	5.267664000
1	-3.256115000	-1.224573000	5.380002000
1	-3.424004000	-3.717865000	5.158480000
1	0.743216000	-7.770201000	4.886725000
1	2.267866000	-5.425793000	5.208993000
1	-1.561067000	-1.323130000	5.805978000
1	-1.714759000	-5.454449000	5.893630000
1	-1.202265000	-3.822818000	6.271226000
1	2.018820000	-3.063111000	6.134907000
1	1.012736000	-5.960140000	6.306586000
1	0.847160000	-3.671350000	7.294812000
1	2.506638000	-4.228206000	7.363718000
42	0.412265000	-2.648531000	3.070140000
7	1.072174000	-1.485326000	4.227138000
15	0.168570000	-1.425726000	0.887663000
15	2.301429000	-3.602023000	1.706621000
15	-1.948181000	-2.406703000	3.717077000
15	0.124567000	-4.688784000	4.464002000
1	1.235722000	-0.829230000	4.987554000
1	-0.664560000	-3.843733000	2.119227000

Final Gibbs free energy (Hartree/particle) = -6305.862228

Mo$_{int}$7

42	3.004336877	18.533644156	24.013958442
15	1.320041549	17.605488353	25.729166650
15	0.849002570	18.517518698	22.677586163
15	4.937533539	18.324236717	25.605640738
15	3.852958114	16.304891531	23.383456616
6	0.397345950	18.873615218	26.731130923
1	-0.055590854	19.555922445	25.994913520
1	-0.431182975	18.384395785	27.269213122
6	0.098221387	20.143568863	22.180025511
1	0.778644131	20.598750114	21.446010647
1	-0.840722956	19.914409022	21.651693453
6	-0.060768347	16.829066894	24.749419284
1	0.334293068	15.893323545	24.326872478
1	-0.885918978	16.559630833	25.425992523
6	6.111686998	19.756178868	25.526291053
1	7.076787417	19.447317368	25.959908195
1	6.277489840	19.941503007	24.453892100
6	1.275422096	19.669988953	27.695239350
1	2.100129872	20.168981847	27.161920666
1	1.711827398	19.030006555	28.478405005
1	0.686852880	20.449185890	28.203539915
6	0.842504887	17.554992384	21.087517994
1	1.775984512	17.812450063	20.570199267
1	0.952909553	16.503544421	21.398389966
6	-0.551444898	17.760611046	23.645941901
1	-1.126379188	18.590587413	24.081961973
1	-1.233899912	17.238355594	22.957285665
6	1.679968811	16.280827682	26.992632282
1	2.352235364	15.558658860	26.501121853
1	2.279941567	16.768028032	27.774804173
6	6.006609314	18.081682600	28.277339240
1	6.463864763	19.081619009	28.280588516
1	6.767448889	17.365993277	27.929807246
1	5.773611726	17.825179421	29.322676614
6	-0.161888135	21.129700662	23.314461841
1	-0.634676020	22.039981127	22.918096294
1	0.768182563	21.441249063	23.807871287
1	-0.838186158	20.716153984	24.079256865
6	5.581676923	21.014160179	26.213887284
1	4.571090867	21.266239878	25.859625998
1	6.235341998	21.875584497	26.008456312
1	5.526723356	20.894292241	27.306505820
6	4.735750075	18.029887260	27.428004052
1	3.992571456	18.756843980	27.787838408
1	4.264598689	17.040093469	27.513251925
6	-0.349925427	17.721375512	20.147516420
1	-0.267596044	17.017002736	19.304825408
1	-0.394712776	18.734840707	19.721707017
1	-1.312438814	17.524280473	20.646413638
6	5.989529015	16.881428425	25.082853897
1	6.614000241	17.223441463	24.240914833
1	6.679998311	16.591563993	25.890066045
6	4.921510732	16.184445022	21.855550444
1	5.752629445	16.895853123	22.002567195
1	5.387063430	15.186425147	21.850772692
6	0.485592994	15.563546400	27.623029815
1	-0.071225022	14.963769849	26.887956697
1	-0.221089238	16.267658029	28.089350286
1	0.826717554	14.876073162	28.413048685
6	5.093430745	15.720304890	24.656805788
1	4.526043540	15.332833223	25.519135970
1	5.687804168	14.883681828	24.258439376
6	2.744986157	14.816898168	23.234436942
1	2.261319040	14.725914701	24.221264468
1	1.943409527	15.092262969	22.531141922
6	4.218131790	16.464558458	20.529870831
1	4.919254080	16.360851241	19.687692921
1	3.817886667	17.486096725	20.496552056
1	3.385886511	15.764746846	20.356163275
6	3.381426939	13.489037140	22.824655046
1	3.797732152	13.531086139	21.806966067

1	2.630452929	12.683290416	22.836208440
1	4.190962809	13.190040019	23.508083804
7	4.200566708	19.333137296	22.642081259
1	4.961880559	18.722493507	22.326270630
14	4.372588343	20.790268854	21.642103329
1	5.790709918	21.242528613	21.794858627
6	3.222380888	22.183015326	22.143466912
6	2.329350920	22.787066240	21.239487495
6	3.306450976	22.720760205	23.442882150
6	1.526650816	23.864366723	21.628273731
6	2.507398598	23.795565671	23.837445029
6	1.609563068	24.366681833	22.929352576
1	2.266347266	22.426345061	20.210372852
1	4.016776499	22.300079614	24.157625022
1	0.841197821	24.319162938	20.908734457

Deprot-Mo$_{int}$7

42	2.447272000	18.517226000	24.116122000
15	1.305342000	17.067682000	25.764214000
15	0.224298000	18.251289000	23.117403000
15	4.549399000	18.947284000	25.252305000
15	3.716931000	16.545373000	23.499182000
6	0.231553000	17.887906000	27.060825000
1	-0.485360000	18.501696000	26.495515000
1	-0.352225000	17.102518000	27.571018000
6	-0.848658000	19.783151000	23.040412000
1	-0.415732000	20.408207000	22.238809000
1	-1.868771000	19.518237000	22.715620000
6	-0.050972000	16.093955000	24.902073000
1	0.443291000	15.334950000	24.277782000
1	-0.690843000	15.565435000	25.626532000
6	5.366047000	20.622481000	25.197057000
1	6.372005000	20.570143000	25.646397000
1	5.509500000	20.869124000	24.136011000
6	0.951842000	18.766602000	28.077797000
1	1.499445000	19.572338000	27.566949000
1	1.673329000	18.195036000	28.683692000
1	0.231994000	19.228543000	28.773370000
6	0.079683000	17.658055000	21.349139000
1	0.785700000	18.273585000	20.767555000
1	0.512892000	16.645000000	21.359561000
6	-0.880273000	17.041029000	24.033094000
1	-1.568211000	17.630539000	24.659063000
1	-1.508471000	16.486512000	23.31838600
6	2.116176000	15.763352000	26.855029000
1	2.986734000	15.377010000	26.305755000
1	2.536444000	16.334660000	27.698230000
6	5.856772000	18.921003000	27.872867000
1	6.089070000	19.997028000	27.872082000
1	6.732213000	18.393716000	27.460760000
1	5.757516000	18.609842000	28.926412000
6	-0.879767000	20.566113000	24.351612000
1	-1.422012000	21.518194000	24.233032000
1	0.140038000	20.779863000	24.707130000
1	-1.385689000	19.997333000	25.148489000
6	4.512358000	21.695614000	25.874251000
1	3.482078000	21.673199000	25.488933000
1	4.920621000	22.701917000	25.690666000
1	4.458625000	21.546146000	26.964783000
6	4.578807000	18.612017000	27.094464000

1	2.590858178	24.196329144	24.850884004
1	0.985128807	25.210691399	23.232519616
6	4.158343537	20.225070475	19.861233765
6	5.297906834	19.951528072	19.082082366
6	2.898728794	19.916064495	19.312016585
6	5.185836300	19.381546582	17.809839386
6	2.778638153	19.350251986	18.041159849
6	3.925579272	19.076503243	17.288516727
1	6.292038780	20.185937206	19.474361231
1	1.990902963	20.113200129	19.884672369
1	6.084524631	19.178150045	17.222109568
1	1.789376790	19.122468970	17.635774940
1	3.835881050	18.632709856	16.294002719
1	2.327707518	20.212461767	24.108040679
1	2.766939791	20.135218802	24.856707149

Final Gibbs free energy (Hartree/particle) = -7061.881774

1	3.725436000	19.167769000	27.508195000
1	4.314145000	17.548289000	27.194115000
6	-1.296048000	17.655672000	20.683390000
1	-1.244985000	17.194332000	19.683014000
1	-1.690370000	18.674948000	20.552436000
1	-2.037480000	17.086314000	21.267390000
6	5.910757000	17.797395000	24.681535000
1	6.292881000	18.201048000	23.730489000
1	6.756444000	17.773556000	25.387386000
6	4.441368000	16.660905000	21.779313000
1	4.983087000	17.616213000	21.749632000
1	5.201351000	15.867267000	21.687865000
6	1.256413000	14.610521000	27.372178000
1	0.912823000	13.960133000	26.552521000
1	0.362694000	14.971586000	27.905248000
1	1.824123000	13.979678000	28.076592000
6	5.314373000	16.403577000	24.474678000
1	5.069101000	15.940107000	25.444866000
1	6.019185000	15.730288000	23.961234000
6	3.094118000	14.780127000	23.482376000
1	2.658078000	14.586330000	24.472532000
1	2.242904000	14.790269000	22.781436000
6	3.445550000	16.591400000	20.628836000
1	3.966246000	16.659378000	19.661211000
1	2.742136000	17.429710000	20.679780000
1	2.868520000	15.652236000	20.637611000
6	4.080654000	13.670872000	23.117620000
1	4.508755000	13.812608000	22.113371000
1	3.581163000	12.687787000	23.123426000
1	4.916734000	13.615869000	23.832307000
7	2.836377000	19.776394000	22.524030000
1	2.005443000	20.016663000	21.977684000
14	4.220195000	20.382507000	21.684536000
1	5.482580000	19.808256000	22.233677000
6	4.306545000	22.268036000	21.759996000
6	5.153097000	23.004112000	20.910504000
6	3.508868000	22.975595000	22.676068000
6	5.209224000	24.398191000	20.979469000
6	3.558991000	24.371388000	22.749300000
6	4.411231000	25.084842000	21.901607000
1	5.772215000	22.479835000	20.175293000
1	2.839422000	22.416762000	23.335237000
1	5.872042000	24.953415000	20.309788000
1	2.930469000	24.903797000	23.468702000

1	4.451725000	26.176277000	21.955130000
6	4.205893000	19.874566000	19.861153000
6	5.325366000	19.278976000	19.255674000
6	3.047218000	20.035411000	19.078359000
6	5.291516000	18.850321000	17.924199000
6	3.001624000	19.606560000	17.750152000
6	4.126065000	19.009295000	17.169725000
1	6.240073000	19.133641000	19.839130000

Mo_{int}8

42	3.050258000	18.484896000	24.023870000
15	1.196564000	17.655755000	25.310374000
15	1.119038000	18.538967000	22.382707000
15	5.071577000	18.430083000	25.442917000
15	3.769298000	16.197397000	23.778714000
6	0.127941000	18.955610000	26.120881000
1	-0.081288000	19.694991000	25.333819000
1	-0.837276000	18.499591000	26.394202000
6	0.339868000	20.125080000	21.790248000
1	1.154461000	20.706802000	21.343059000
1	-0.356072000	19.877406000	20.974139000
6	-0.087826000	16.790414000	24.264116000
1	0.326713000	15.813317000	23.986563000
1	-1.003368000	16.602896000	24.845923000
6	6.619409000	19.342086000	24.921843000
1	7.438422000	19.024961000	25.587640000
1	6.900422000	18.964567000	23.921654000
6	0.756456000	19.641852000	27.332072000
1	1.754223000	20.040144000	27.093064000
1	0.875204000	18.950681000	28.180852000
1	0.130955000	20.480456000	27.676887000
6	1.470053000	17.666242000	20.770076000
1	2.411718000	18.083142000	20.383999000
1	1.686979000	16.626462000	21.062116000
6	-0.393020000	17.625609000	23.022493000
1	-1.147003000	18.388518000	23.262253000
1	-0.819414000	17.013045000	22.213661000
6	1.441825000	16.458570000	26.716303000
1	1.931648000	15.574348000	26.281887000
1	2.194336000	16.921999000	27.375146000
6	6.109931000	19.028980000	28.096806000
1	6.846423000	19.769843000	27.750652000
1	6.615982000	18.052071000	28.145522000
1	5.833710000	19.302301000	29.127470000
6	-0.373223000	20.957035000	22.853239000
1	-0.684439000	21.925331000	22.431952000
1	0.279630000	21.167438000	23.708690000
1	-1.282062000	20.461183000	23.227187000
6	6.496413000	20.864484000	24.909499000
1	5.712812000	21.214516000	24.222851000
1	7.442936000	21.332472000	24.597508000
1	6.247092000	21.257509000	25.907106000
6	4.870032000	18.993232000	27.206386000
1	4.395199000	19.986480000	27.152571000
1	4.100805000	18.323736000	27.625296000
6	0.415975000	17.712130000	19.664739000
1	0.708286000	17.047707000	18.836218000
1	0.314921000	18.723410000	19.246100000
1	-0.575401000	17.384051000	20.015589000
6	5.787708000	16.702726000	25.622734000
1	6.646006000	16.630778000	24.935574000
1	6.188711000	16.563198000	26.637696000

6	5.091173000	15.904602000	22.470502000
1	5.845240000	16.698149000	22.598228000
1	2.157598000	20.503380000	19.513150000
1	6.174514000	18.386182000	17.476024000
1	2.087910000	19.736926000	17.163636000
1	4.092785000	18.670373000	16.130832000
1	2.051894000	19.357721000	25.566430000

Final Gibbs free energy (Hartree/particle) = -7061.410067

1	5.605632000	14.965190000	22.728057000
6	0.206800000	16.032665000	27.511088000
1	-0.542277000	15.541536000	26.871247000
1	-0.282794000	16.884197000	28.006283000
1	0.485473000	15.313335000	28.297580000
6	4.743745000	15.647368000	25.278823000
1	4.025130000	15.526966000	26.102175000
1	5.212006000	14.666899000	25.102280000
6	2.671481000	14.715299000	23.478582000
1	1.977817000	14.680982000	24.331474000
1	2.052619000	14.964877000	22.600977000
6	4.622060000	15.843549000	21.017500000
1	5.473961000	15.671447000	20.340474000
1	4.122523000	16.764572000	20.684951000
1	3.904903000	15.025173000	20.853731000
6	3.335034000	13.348404000	23.302744000
1	3.992685000	13.309976000	22.421851000
1	2.568823000	12.568269000	23.169306000
1	3.935013000	13.066078000	24.180997000
7	4.481143000	19.134773000	22.284189000
1	4.262675000	18.440580000	21.567920000
14	4.373498000	20.786649000	21.557546000
1	5.772920000	21.294089000	21.481293000
6	3.340830000	21.856350000	22.691912000
6	2.932092000	23.147608000	22.307230000
6	2.987794000	21.412096000	23.979117000
6	2.194385000	23.959314000	23.174404000
6	2.265501000	22.223611000	24.856384000
6	1.862257000	23.499226000	24.452067000
1	3.202938000	23.539236000	21.322498000
1	3.421396000	20.478616000	24.410122000
1	1.888576000	24.958572000	22.855661000
1	2.017870000	21.855437000	25.853197000
1	1.294057000	24.136086000	25.134070000
6	3.665398000	20.543230000	19.837477000
6	4.236981000	19.546433000	19.019913000
6	2.580064000	21.274262000	19.323140000
6	3.724491000	19.268297000	17.751249000
6	2.066644000	21.004345000	18.051155000
6	2.631732000	19.995166000	17.267275000
1	5.100240000	18.971900000	19.372252000
1	2.117257000	22.062297000	19.920304000
1	4.180088000	18.488375000	17.136409000
1	1.220634000	21.582928000	17.672508000
1	2.227119000	19.780254000	16.275356000
1	2.759390000	18.952696000	25.610910000
1	5.434249000	18.924962000	22.577736000

Final Gibbs free energy (Hartree/particle) = -7061.894058

Pyridine

6	-2.734379000	0.000884000	-4.029025000
6	-2.734379000	1.224905000	-3.356778000
6	-2.734379000	-1.174222000	-3.274580000
1	-2.734379000	2.171492000	-3.902417000
1	-2.734379000	-2.155894000	-3.754223000
6	-2.734379000	1.215786000	-1.957675000
6	-2.734379000	-1.069372000	-1.879324000
1	-2.734379000	2.161493000	-1.402064000
1	-2.734379000	-1.974900000	-1.260378000
7	-2.734380000	0.096863000	-1.228372000
1	-2.734380000	-0.036479000	-5.121696000

Final Gibbs free energy (Hartree/particle) = -248.2472324

H_2

1	0.201774000	0.000000000	-0.324840000
1	0.201774000	0.000000000	-1.069185000

Final Gibbs free energy (Hartree/particle) = -1.17352228

Pyridinium

6	-2.734379000	0.001572000	-4.009840000
6	-2.734379000	1.237900000	-3.351472000
6	-2.734379000	-1.186786000	-3.268373000
1	-2.734379000	2.178282000	-3.905106000
1	-2.734379000	-2.162787000	-3.756468000
6	-2.734379000	1.261618000	-1.965141000
6	-2.734379000	-1.115652000	-1.883705000
1	-2.734379000	2.179814000	-1.374936000
1	-2.734380000	-1.991316000	-1.232063000
7	-2.734379000	0.094954000	-1.283641000
1	-2.734379000	-0.035909000	-5.102084000
1	-2.734380000	0.129992000	-0.262662000

Final Gibbs free energy (Hartree/particle) = -248.6916142

Time-dependent DFT (TDDFT) Calculations of Mo4.

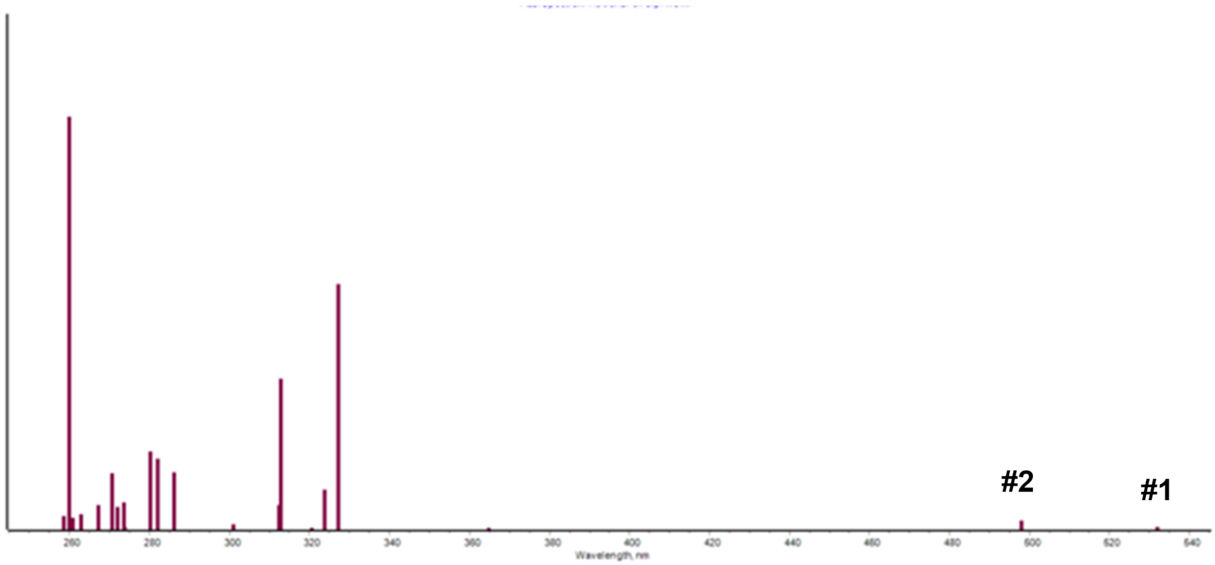

Figure S60. Full data of the TDDFT calculations of **Mo4**.

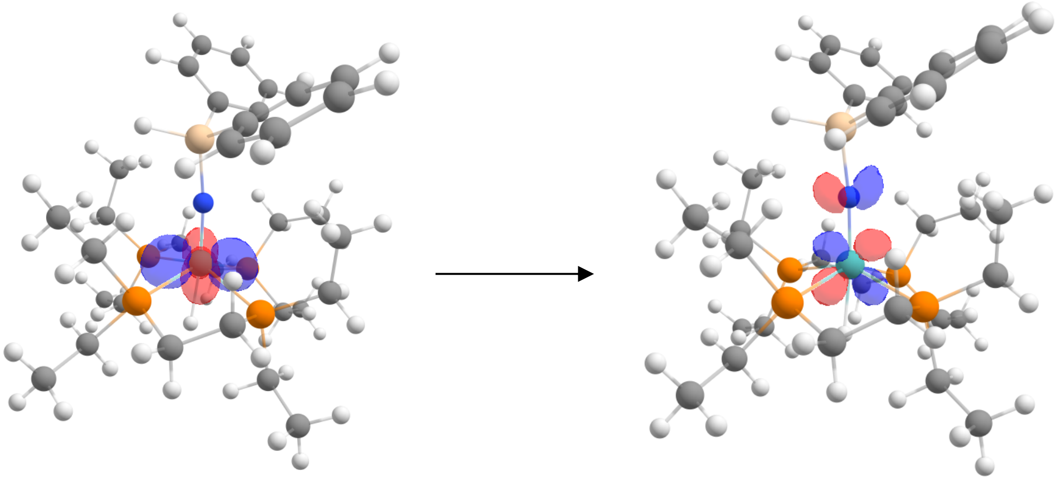

Figure S61. Natural transition orbital plots of the dominant transitions from transition #1 (98.9%) and transition #2 (98.2%).

pK_a Calculation of $Mo_{int}7$.

$Mo_{int}7$ + pyridine ⟶ **Deprot-$Mo_{int}7$** + pyridinium ΔG = 17.1 kcal/mol (Eq. 1)

pyridinium ⟶ pyridine + H^+ ΔG = 7.5 kcal/mol (Eq. 2)

$Mo_{int}7$ ⟶ **Deprot-$Mo_{int}7$** + H^+ ΔG = 24.6 kcal/mol (Eq. 3)

pK_a = 18.0

VII. X-Ray Crystallographic Data

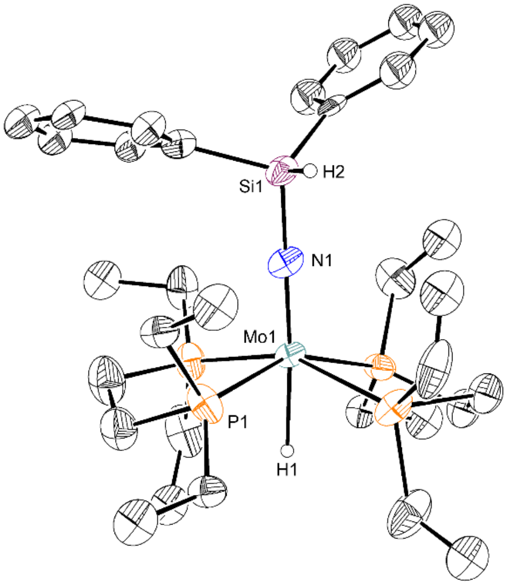

Figure S62. Representations of the solid-state structure of **Mo4** at 30% probability ellipsoids. [BAr^F] anion and hydrogen atoms except H1 and H2 are omitted for clarity.

Table S7. Crystal data for **Mo4** (CCDC 2503648)

Crystal data

$C_{32}H_{58.282}MoNP_4Si \cdot C_{32}H_{12.298}BF_{23.702}$	*F*(000) = 3184
M_r = 1562.86	D_x = 1.419 Mg m^{-3}
Monoclinic, *P*2$_1$/*c*	Cu *K*a radiation, l = 1.54184 Å
a = 15.3512 (2) Å	Cell parameters from 26217 reflections
b = 17.1569 (2) Å	q = 2.9–74.9°
c = 28.4741 (3) Å	m = 3.33 mm^{-1}

b = 102.755 (1)°	T = 100 K
V = 7314.42 (15) Å^3	Block, dark pink
Z = 4	0.34 × 0.16 × 0.13 mm

Data collection

XtaLAB Synergy, Dualflex, HyPix-Arc 150 diffractometer	14441 independent reflections
Radiation source: micro-focus sealed X-ray tube, PhotonJet (Cu) X-ray Source	11139 reflections with I > 2s(I)
Mirror monochromator	R_{int} = 0.039
Detector resolution: 10.0000 pixels mm^{-1}	q_{max} = 75.4°, q_{min} = 3.0°
w scans	h = -19®17
Absorption correction: multi-scan *CrysAlis PRO* 1.171.43.131a (Rigaku Oxford Diffraction, 2024) Empirical absorption correction using spherical harmonics, implemented in SCALE3 ABSPACK scaling algorithm.	k = -21®17
T_{min} = 0.649, T_{max} = 1.000	l = -33®35
59126 measured reflections	

Refinement

Refinement on F^2	Primary atom site location: dual
Least-squares matrix: full	Hydrogen site location: mixed
$R[F^2 > 2s(F^2)]$ = 0.096	H atoms treated by a mixture of independent and constrained refinement
$wR(F^2)$ = 0.299	$w = 1/[s^2(F_o^2) + (0.1871P)^2 + 16.1466P]$ where $P = (F_o^2 + 2F_c^2)/3$
S = 1.06	$(D/s)_{max}$ = 0.002
14441 reflections	$D\rho_{max}$ = 2.07 e Å^{-3}
982 parameters	$D\rho_{min}$ = -1.40 e Å^{-3}
2406 restraints	

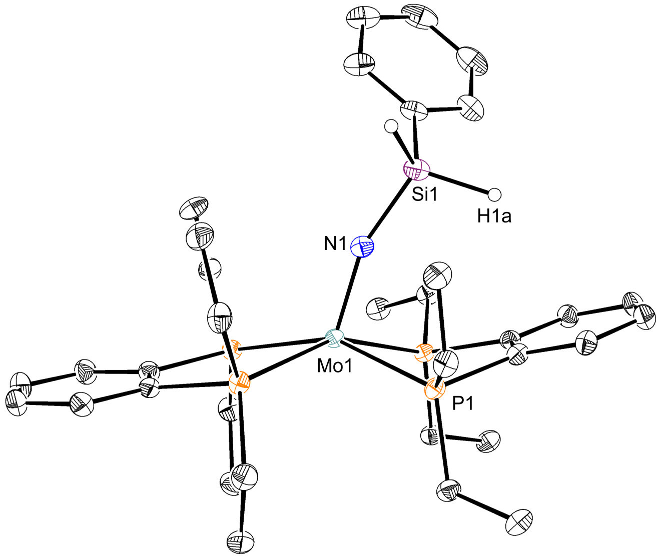

Figure S63. Representations of the solid-state structure of **Mo9** at 30% probability ellipsoids. [BAr^F] anion and hydrogen atoms except H1a are omitted for clarity. Mo-*H* was not able to be located.

Table S8. Crystal data for **Mo9** (CCDC 2503649)

Crystal data

0.131($C_{34}H_{55}MoNP_4Si$)·0.131($C_{32}H_{12}BF_{24}$)	*F*(000) = 6456
M_r = 208.38	D_x = 1.499 Mg m^{-3}
Monoclinic, *I*2/*a*	Cu *K*a radiation, l = 1.54184 Å
a = 25.2818 (2) Å	Cell parameters from 24374 reflections
b = 13.2257 (1) Å	q = 3.5–74.8°
c = 42.8129 (4) Å	m = 3.48 mm^{-1}
b = 100.422 (1)°	*T* = 100 K
V = 14079.2 (2) Å^3	Plate, clear pinkish red
Z = 61	0.58 × 0.43 × 0.03 mm

Data collection

XtaLAB Synergy, Dualflex, HyPix-Arc 150 diffractometer	13970 independent reflections

Radiation source: micro-focus sealed X-ray tube, PhotonJet (Cu) X-ray Source	11499 reflections with I > 2s(I)
Mirror monochromator	R_{int} = 0.038
Detector resolution: 10.0000 pixels mm^{-1}	q_{max} = 75.8°, q_{min} = 3.5°
w scans	h = -31®24
Absorption correction: multi-scan *CrysAlis PRO* 1.171.43.143a (Rigaku Oxford Diffraction, 2024) Empirical absorption correction using spherical harmonics, implemented in SCALE3 ABSPACK scaling algorithm.	k = -15®15
T_{min} = 0.684, T_{max} = 1.000	l = -52®53
62421 measured reflections	

Refinement

Refinement on F^2	Primary atom site location: dual
Least-squares matrix: full	Hydrogen site location: mixed
$R[F^2 > 2s(F^2)]$ = 0.061	H atoms treated by a mixture of independent and constrained refinement
$wR(F^2)$ = 0.168	$w = 1/[s^2(F_o^2) + (0.0849P)^2 + 91.7603P]$ where $P = (F_o^2 + 2F_c^2)/3$
S = 1.04	$(D/s)_{max}$ = 0.003
13970 reflections	$Dñ_{max}$ = 1.58 e Å^{-3}
895 parameters	$Dñ_{min}$ = -1.03 e Å^{-3}
0 restraints	

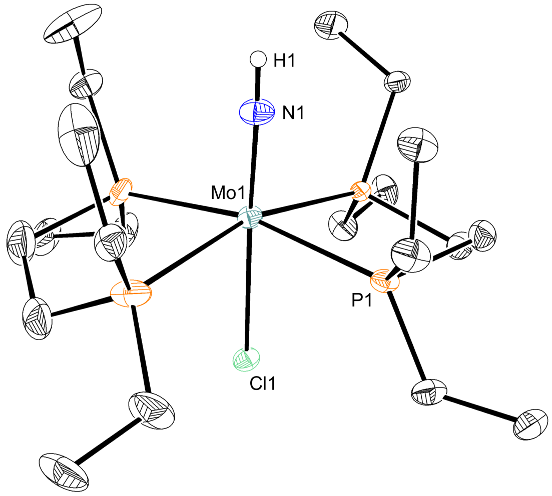

Figure S64. Representations of the solid-state structure of **Mo13** at 30% probability ellipsoids. $[BAr^F]$ anion and hydrogen atoms except H1 are omitted for clarity.

Table S9. Crystal data for **Mo13** (CCDC 2503651)

Crystal data

$0.5(C_{20}H_{49}ClMoNP_4)·0.5(C_{32}H_{12}BF_{24})$	*F*(000) = 2880
M_r = 711.05	D_x = 1.550 Mg m^{-3}
Monoclinic, *P*2_1/*c*	Cu *K*a radiation, l = 1.54184 Å
a = 14.6604 (1) Å	Cell parameters from 52937 reflections
b = 14.6008 (1) Å	q = 3.0–75.8°
c = 29.3443 (3) Å	m = 4.15 mm^{-1}
b = 104.081 (1)°	*T* = 100 K
V = 6092.52 (9) $Å^3$	Block, clear pinkish red
Z = 8	0.32 × 0.25 × 0.18 mm

Data collection

XtaLAB Synergy, Dualflex, HyPix-Arc 150 diffractometer	12306 independent reflections

Radiation source: micro-focus sealed X-ray tube, PhotonJet (Cu) X-ray Source	11196 reflections with I > 2s(I)
Mirror monochromator	R_{int} = 0.052
Detector resolution: 10.0000 pixels mm^{-1}	q_{max} = 75.9°, q_{min} = 3.1°
w scans	h = -17®18
Absorption correction: multi-scan *CrysAlis PRO* 1.171.43.143a (Rigaku Oxford Diffraction, 2024) Empirical absorption correction using spherical harmonics, implemented in SCALE3 ABSPACK scaling algorithm.	k = -18®16
T_{min} = 0.629, T_{max} = 1.000	l = -35®36
90095 measured reflections	

Refinement

Refinement on F^2	Primary atom site location: dual
Least-squares matrix: full	Hydrogen site location: mixed
$R[F^2 > 2s(F^2)]$ = 0.064	H atoms treated by a mixture of independent and constrained refinement
$wR(F^2)$ = 0.171	$w = 1/[s^2(F_o^2) + (0.0891P)^2 + 19.7732P]$ where $P = (F_o^2 + 2F_c^2)/3$
S = 1.07	$(D/s)_{max}$ = 0.001
12306 reflections	$Dñ_{max}$ = 1.91 e Å^{-3}
814 parameters	$Dñ_{min}$ = -1.51 e Å^{-3}
79 restraints	

VII. References

1. Pangborn, A. B.; Giardello, M. A.; Grubbs, R. H.; Rosen, R. K.; Timmers, F. J. Safe and Convenient Procedure for Solvent Purification. *Organometallics* **1996**, *15*, 1518–1520.

2. Inoue, R.; Yamaguchi, M.; Murakami, Y.; Okano, K.; Mori, A. Revisiting of Benzophenone Ketyl Still: Use of a Sodium Dispersion for the Preparation of Anhydrous Solvents. *ACS Omega* **2018**, *3*, 12703–12706.

3. Katayama, A.; Ohta, T.; Wasada-Tsutsui, Y.; Inomata, T.; Ozawa, T.; Ogura, T.; Masuda, H. Dinitrogen-Molybdenum Complex Induces Dinitrogen Cleavage by One-Electron Oxidation. *Angew. Chem. Int. Ed.* **2019**, *131*, 11401–11406.

4. Kim, J.; Panetti, G. B.; Kaul, N.; Kim, S.; Chirik, P. J. Photodriven Ammonia Synthesis from N_2 and H_2: Recycling of a Molecular Molybdenum Nitride. *J. Am. Chem. Soc.* **2025**, *147*, 8215–8226.

5. Cugny, J.; Schmalle, H. W.; Fox, T.; Blacque, O.; Alfonso, M.; Berke, H. Hydride Transfer Reactivity of Mo(L)(H)(depe)$_2$ (L = N, NBEt$_3$). *Eur. J. Inorg. Chem.* **2006**, 540−552.

6. Prince, P. D.; Bearpark, M. J.; McGrady, G. S.; Steed, J. W. Hypervalent hydridosilicates: synthesis, structure and hydride bridging. *Dalton Trans.* **2008**, 271–282.

7. Fulmer, G. R.; Miller, A. J. M.; Sherden, N. H.; Gottlieb, H. E.; Nudelman, A.; Stoltz, B. M.; Bercaw, J. E.; Goldberg, K. I. NMR Chemical Shifts of Trace Impurities: Common Laboratory Solvents, Organics, and Gases in Deuterated Solvents Relevant to the Organometallic Chemist. *Organometallics* **2010**, *29*, 2176–2179.

8. Shields, B. J.; Kudisch, B.; Scholes, G. D.; Doyle, A. G. Long-Lived Charge-Transfer States of Nickel(II) Aryl Halide Complexes Facilitate Bimolecular Photoinduced Electron Transfer. *J. Am. Chem. Soc.* **2018**, *140*, 3035–3039.

9. Bydder, M.; Rahal, A.; Fullerton, G. D.; Bydder, G. M. The magic angle effect: A source of artifact, determinant of image contrast, and technique for imaging. *J. Magn. Reson. Imaging*. **2007**, *25*, 290–300.

10. Neese, F. The ORCA program system. *WIREs Comput. Mol. Sci.* **2012**, *2*, 73–78.

11. Zhao, Y.; Truhlar, D. G. The M06 suite of density functionals for main group thermochemistry, thermochemical kinetics, noncovalent interactions, excited states, and transition elements: two new functionals and systematic testing of four M06-class functionals and 12 other functionals, *Theor. Chem. Acc.* **2008**, *120*, 215–241.

12. Staroverov, V. N.; Scuseria, G. E.; Tao, J.; Perdew, J. P. Comparative assessment of a new nonempirical density functional: Molecules and hydrogen-bonded complexes, *J. Chem. Phys.* **2003**, *119*, 12129.

13. Schäfer, A.; Horn, H.; Ahlrichs, R. Fully optimized contracted Gaussian basis sets for atoms Li to Kr. *J. Chem. Phys.* **1992**, *97*, 2571–2577.

14. Schäfer, A.; Huber, C.; Ahlrichs, R. Fully optimized contracted Gaussian basis sets of triple zeta valence quality for atoms Li to Kr. *J. Chem. Phys.* **1994**, *100*, 5829–5835.

15. Weigend, F.; Ahlrichs, R. Balanced basis sets of split valence, triple zeta valence and quadruple zeta valence quality for H to Rn: Design and assessment of accuracy. *Phys. Chem. Chem. Phys.* **2005**, *7*, 3297–3305.

16. Rolfes, J. D.; Neese, F.; Pantazis, D. A. All-electron scalar relativistic basis sets for the elements Rb–Xe. *J. Comput. Chem*. **2020**, *41*, 1842–1849.

17. Weigend, F. Accurate Coulomb-fitting basis sets for H to Rn. *Phys. Chem. Chem. Phys.* **2006**, *8*, 1057–1065.

18. Pantazis, D. A.; Neese, F. All-Electron Scalar Relativistic Basis Sets for the Lanthanides, *J. Chem. Theory Comput.* **2009**, *5*, 2229–2238.

19. Neese, F.; Wennmohs, F.; Hansen, A.; Becker, U. Efficient, Approximate and parallel Hartree–Fock and hybrid DFT calculations. A 'chain-of-spheres' algorithm for the Hartree–Fock exchange. *Chem. Phys.* **2009**, *356*, 98–109.

20. Kossmann, S.; Neese, F. Comparison of two efficient approximate Hartee–Fock approaches. *Chem. Phys. Lett.* **2009**, *481*, 240–243.

21. Neese, F. An improvement of the resolution of the identity approximation for the formation of the Coulomb matrix. *J. Comput. Chem.* **2003**, *24*, 1740–1747.

22. Weigend, F.; Ahlrichs, R. Balanced basis sets of split valence, triple zeta valence and quadruple zeta valence quality for H to Rn: Design and assessment of accuracy. *Phys. Chem. Chem. Phys.* **2005**, *7*, 3297–3305.

23. Barone, V.; Cossi, M. Quantum Calculation of Molecular Energies and Energy Gradients in Solution by a Conductor Solvent Model. *J. Phys. Chem.* **1998***, 102*, 1995–2001.
